# Diagnostic performance of artificial intelligence in early diagnosis of mosquito-borne viral infections: Systematic review and meta-analysis

**DOI:** 10.1371/journal.pdig.0001796

**Published:** 2026-10-27

**Authors:** Flavia Pennisi, Antonio Pinto, Claudia Cozzolino Cangiano, Giovanni Rezza, Carlo Signorelli, Vincenzo Baldo, Vincenza Gianfredi

**Affiliations:** 1 Faculty of Medicine, University Vita-Salute San Raffaele, Milan, Italy; 2 PhD National Program in One Health Approaches to Infectious Diseases and Life Science Research, Department of Public Health, Experimental and Forensic Medicine, University of Pavia, Pavia, Italy; 3 Department of Cardiac, Thoracic, Vascular Sciences, and Public Health, University of Padua, Padua, Italy; Xinjiang Medical University Affiliated First Hospital, CHINA

## Abstract

Early diagnosis of mosquito-borne viral infections is challenging because initial symptoms are often nonspecific and confirmatory testing may be delayed or unavailable. We synthesised evidence on artificial intelligence and machine learning models for the early diagnosis of dengue, chikungunya, and Zika. We conducted a PRISMA 2020-compliant systematic review and meta-analysis (PROSPERO CRD420251129670). PubMed/MEDLINE, Embase, and Scopus were searched from inception to 11 August 2025 for model-development or validation studies reporting diagnostic performance. Area under the receiver operating characteristic curve (AUC), accuracy, positive predictive value (PPV), and negative predictive value (NPV) were pooled using random-effects models, while sensitivity and specificity were jointly synthesised using a bivariate random-effects model. Risk of bias was assessed using the Prediction model Risk Of Bias Assessment Tool (PROBAST). Database searches identified 4,531 records, of which 43 studies met the inclusion criteria. The evidence was predominantly from Asia and overwhelmingly focused on dengue; no eligible studies evaluated West Nile virus, yellow fever, or Rift Valley fever. Pooled estimates were 0.954 (95% CI 0.936–0.968) for AUC, 0.904 (95% CI 0.888–0.918) for accuracy, 0.903 (95% CI 0.876–0.925) for PPV, and 0.882 (95% CI 0.859–0.902) for NPV. The bivariate pooled sensitivity was 0.861 (95% CI 0.809–0.900) and specificity was 0.902 (95% CI 0.871–0.927). Heterogeneity was substantial for the univariate outcomes (I^2^ 95.8%–97.7%). Most studies were at high overall risk of bias, while calibration and external validation were uncommon. Artificial intelligence models showed encouraging diagnostic performance in the published literature, but the pooled estimates should be interpreted as descriptive summaries of a highly heterogeneous evidence base rather than as definitive estimates of real-world performance. Prospective, externally validated, well-calibrated, and geographically diverse studies are needed before routine clinical implementation can be recommended.

## Author summary

Mosquito-borne viral infections such as dengue, chikungunya, and Zika can be difficult to distinguish early because their initial symptoms often overlap and laboratory confirmation may not be immediately available. We reviewed 43 studies that tested artificial intelligence and machine-learning approaches for supporting early diagnosis. We found that published models generally reported high diagnostic performance, but the evidence was highly heterogeneous and was dominated by dengue studies conducted in Asia. Many studies were retrospective, most had important risks of bias, and external validation and calibration were uncommon. We also found that no single type of artificial intelligence model consistently performed better than the others. These findings suggest that artificial intelligence could eventually support earlier diagnosis and triage, but current performance estimates should not be interpreted as evidence that the models will work equally well in routine care or across different populations. We believe future research should prioritize prospective testing, external validation in diverse countries and healthcare settings, transparent reporting, calibration, and evaluation of how these tools perform when integrated into real clinical workflows.

## 1 Introduction

Mosquito-borne viral infections, such as dengue, chikungunya, and Zika, remain a major cause of sporadic cases, small clusters or large-scale outbreaks of acute febrile illness, with a disproportionate impact in tropical and subtropical regions, as well as in health systems where rapid confirmatory testing is not largely available [1]. Early case identification is pivotal: it supports timely clinical triage and supportive management, enables appropriate use of diagnostic resources, and strengthens surveillance and outbreak response [2,3]. However, early clinical presentations are frequently non-specific and overlap with other endemic febrile conditions, limiting the discriminatory value of symptoms and routine bedside assessments alone [4].

Laboratory reference standards, such as nucleic acid amplification testing (e.g., Reverse Transcription Polymerase Chain Reaction, RT-PCR), antigen detection (e.g., Non-Structural Protein 1 (NS1) antigen for dengue), and serological assays, provide diagnostic certainty; however, they can be resource-intensive, variably accessible, and not always available at the point of care or within clinically actionable timeframes [5,6]. As a result, there is a strong interest in approaches that can leverage information already available at, or near, the first presentation, to support earlier and more accurate decision-making [7].

In parallel, artificial intelligence (AI) and machine learning (ML) methods have advanced rapidly and are increasingly applied to diagnostic problems in infectious diseases [8–11]. For arboviral infections, AI/ML models have been trained on heterogeneous data modalities, ranging from structured clinical and laboratory variables to biomarkers and, in a minority of studies, microscopy-based image inputs and other high-dimensional data [12,13]. Yet published reports vary widely in reference standards, modelling pipelines, validation strategies, and performance reporting; these factors can inflate apparent accuracy and hinder comparability across studies. Moreover, the extent to which reported performance translates across populations, settings, and pathogens remains uncertain, particularly when external validation and calibration are absent or infrequently reported [14,15].

To address these gaps, we conducted a systematic review and meta-analysis to synthesise the diagnostic accuracy of AI/ML models for early diagnosis of major mosquito-borne viral infections, and to explore how differences in populations, data sources, modelling choices, and validation strategies may reshape performance and generalizability in future real-world use.

## 2 Materials and methods

### 2.1 Search strategy

This systematic review was conducted following the Preferred Reporting Items for Systematic Reviews and Meta-Analyses (PRISMA 2020) guidelines. In addition, the reporting was informed, where applicable, by emerging AI-specific recommendations, including the principles outlined in the PRISMA-AI framework, to improve transparency in the reporting of AI-based evidence synthesis [16,17]. The protocol was prospectively registered in PROSPERO (CRD420251129670). A comprehensive literature search was conducted in PubMed/MEDLINE, Embase, and Scopus up to August 11, 2025. The search strategy combined controlled vocabulary (MeSH terms) with free-text keywords covering AI, ML, deep learning, and mosquito-borne diseases (including dengue, Zika, chikungunya, West Nile, yellow fever, and Rift Valley fever). We manually screened the reference lists of included studies and relevant reviews and contacted field experts to locate additional publications or unpublished data. The complete search string for each database is provided in S1 Appendix.

This review was conducted in accordance with the PRISMA 2020 statement [18] and, where applicable to reviews of diagnostic accuracy studies, the PRISMA-DTA [19] reporting recommendations.

### 2.2 Eligibility criteria

We included studies enrolling individuals of any age evaluated for suspected dengue, Zika, chikungunya, West Nile virus, yellow fever, or Rift Valley fever in any clinical or public health setting, provided that the study addressed early diagnosis (i.e., at or near first presentation or before confirmatory testing). Eligible studies developed, validated, or applied AI/ML-based diagnostic models using clinical features, demographics, epidemiologic information, routine laboratory data, biomarkers, imaging, or other readily available inputs.

To be eligible, studies reported at least one quantitative diagnostic accuracy metric, such as Area Under the Receiver Operating Characteristic Curve (AUC), Sensitivity/Recall, Specificity, Positive Predictive Value (PPV)/Precision, Negative Predictive Value (NPV), Accuracy, F1-score; calibration measures (e.g., Brier score, calibration slope/intercept) were also extracted when available. We included prospective or retrospective diagnostic accuracy studies and AI/ML model development/validation studies (including external validation), whether randomized or nonrandomized, as long as extractable performance data were provided. We retained only original research articles published in peer-reviewed journals and written in English, with no date restrictions, focusing on published literature.

We excluded studies relying exclusively on non-human or vector-only data, laboratory research without human diagnostic outcomes, or analyses based solely on environmental/climatic data without a direct link to human case identification. We also excluded reviews (including systematic reviews and meta-analyses), editorials, letters, commentaries, case reports, guidelines, and trial protocols, as well as conference abstracts/proceedings or other records for which the full text could not be retrieved.

### 2.3 Study selection

All retrieved records were imported into Rayyan (Rayyan Systems Inc., Qatar), a web-based tool designed for systematic review screening, and duplicate records were removed. Two reviewers independently screened titles and abstracts to identify potentially eligible studies. Disagreements were resolved by consensus or, when necessary, by consultation with a third reviewer. Full texts of the selected studies were subsequently reviewed independently for inclusion according to the predefined criteria.

### 2.4 Data extraction

Data were extracted using a pilot-tested Microsoft Excel extraction sheet. We collected bibliographic information (first author and year of publication, continent, country) and study characteristics (study design, study period, setting, population). We also recorded disease-related fields (disease, case definition, disease definition, level of diagnosis, temporal stage of data). Methodological items captured included missing/imbalanced data handling, calibration, data-split, and implementation readiness.

For model inputs, we coded whether each study used clinical data, laboratory data, epidemiological/surveillance data, demographic/socio-economic data, entomological data, environmental data, genomic/virological data, biomarker data, or other sources, recording the specific data source when reported. For each AI/ML model, we extracted the AI/ML Model, the number of variables included versus considered, and the sample size for the training, testing, and validation datasets. When available, classification and regression (Root Mean Square Error (RMSE), Mean Squared Error (MSE), Mean Absolute Error (MAE), R^2^, correlation coefficient r) metrics were recorded. We additionally captured the type of validation (internal/external, specifying the method), and whether model interpretability/explainability was reported. Finally, where available, we extracted the AI model confusion matrix elements.

To ensure comparability across studies, individual AI/ML models were classified into four methodological families for quantitative analyses according to their underlying modelling approach:

(i) Classical ML: Decision Tree (DT/J48), Support Vector Machine (SVM), Bayesian Network (BN), k-Nearest Neighbors (KNN), Linear Discriminant Analysis (LDA), Naive Bayes (NB), Logistic/Multinomial Logistic Regression (LR/MLR), Stacking, Elastic Net, Multivariate Adaptive Regression Splines (MARS), Principal Component Analysis–Discriminant Analysis (PCA-DA), PCA-SVM, PCA-LDA, Partial Least Squares-DA (PLS-DA), Partial Least Squares Regression (PLSR), and related conventional machine-learning approaches;
(ii) Tree-ensemble methods: Random Forest (RF), Extra Trees Classifier (ETC), Adaptive Boosting (AdaBoost), Gradient Boosting Machine/Classifier (GBM/GBC), LightGBM, Extreme Gradient Boosting (XGB), CatBoost, and Classification and Regression Tree (CART);
(iii) Deep learning (DL): Artificial Neural Network (ANN) and ANN-based variants, Multilayer Perceptron (MLP), Extreme Learning Machine (ELM), Bidirectional Long Short-Term Memory (BiLSTM), Convolutional Neural Network (CNN), Gated Recurrent Unit (GRU), Time-Delay/Deep Neural Networks (TLDNN/DNN), Multi-Feedforward Neural Network (MFNN), GPT-3.5, MobileNetV2-based architectures, Generative Adversarial Network (GAN), TabPFN, TabTransformer, and related neural-network architectures; and
(iv) Heuristic approaches: Belief Rule-Based Expert System (BRBES).

At the descriptive study level, studies evaluating multiple standalone algorithms belonging to more than one methodological family were labelled as “Multiple AI/ML approaches” in Table 2. For quantitative analyses, however, each individual model was assigned separately to its corresponding methodological family. No separate “Hybrid” family was used in the quantitative analyses; integrated or optimised pipelines were classified according to the underlying predictive architecture used in the model-level dataset.

### 2.5 Data synthesis and statistical analysis

Results have been reported in accordance with PRISMA 2020, including a PRISMA flow diagram and detailed tables summarizing study characteristics, methodological quality, and performance metrics. A narrative synthesis was first conducted to summarize study characteristics, AI/ML model types, and outcomes. Descriptive performance results were summarised at the individual model level according to the same methodological families used for quantitative analyses; detailed model-level estimates are reported in Table 2. Studies evaluating models from more than one family could therefore contribute models to more than one descriptive family.

Quantitative analyses were performed on performance metrics related to model testing, including AUC, accuracy, sensitivity, specificity, PPV, and NPV. For each metric, values reported by the original studies were prioritized. When not directly reported, sensitivity, specificity, accuracy, PPV, and NPV were derived from the study’s confusion matrix (true positives, true negatives, false positives, and false negatives), when available, allowing inclusion of a larger number of eligible studies than would have been possible using explicitly reported metrics alone. When confusion matrix values were not directly reported, they were reconstructed from the reported performance metrics together with the number of positive and negative cases, where available.

Accuracy, PPV, and NPV were pooled on the logit scale using exact binomial variances. Univariate logit-transformed analyses of sensitivity and specificity were retained only to describe heterogeneity. AUC was pooled on the logit scale, with the standard error estimated using the method of Hanley and McNeil [20] based on the numbers of positive and negative observations when a standard error was not reported. Random-effects models were used for the univariate analyses, and pooled estimates were back-transformed to the original 0–1 scale for reporting. Because sensitivity and specificity are correlated, primary pooled estimates for these metrics were obtained jointly using a Reitsma bivariate random-effects model, which models logit-transformed sensitivity and false-positive rate (1 − specificity) and their correlation across studies [21]. Summary receiver operating characteristic (sROC) curves and the corresponding summary area under the curve (sAUC) were derived from the bivariate model. Model-level AUC values of 1.00 yielding a zero estimated standard error were retained in the descriptive synthesis but were not included in inverse-variance AUC pooling.

Statistical heterogeneity among included studies was assessed for each synthesis using the I^2^ statistic and τ^2^ with exact values reported for each outcome and subgroup, together with the p-value from Cochran’s Q-test. I^2^ values >75% were considered indicative of high heterogeneity, 50–75% moderate, 25–50% low, and <25% negligible [22]. Potential small-study effects and publication bias were assessed through visual inspection of funnel plots and metric-appropriate tests: Kendall’s rank correlation test for accuracy, PPV, NPV, and AUC [23], and Deeks’ funnel plot asymmetry test for sensitivity and specificity [24].

Subgroup analyses were conducted by model type (classes defined above), pathogen, geographic region (continent and country), and study design, and were restricted to strata with a sufficient number of studies to yield a meaningful pooled estimate.

A two-sided p-value <0.05 was considered statistically significant.

Meta-analysis was performed in R version 4.2.2 (R Core Team, Vienna, Austria), using the *metafor* [25], *mada* [26], and *meta* [27] packages. Additional data manipulation and analyses were performed in Python version 3.8.18 (Python Software Foundation, Wilmington, DE, USA). Visualizations were generated using R, Python, Microsoft Excel (Microsoft Corporation, Redmond, WA, USA), and BioRender (BioRender, Toronto, ON, Canada).

### 2.6 Risk of bias assessment

The methodological quality and risk of bias of the included studies were assessed using the Prediction model Risk Of Bias Assessment Tool (PROBAST) [28]. PROBAST was selected because the included evidence primarily consisted of multivariable diagnostic prediction model development and validation studies rather than evaluations of a single conventional index test. Its domain structure therefore allowed assessment of prediction-model-specific sources of bias, particularly those related to predictor handling, model development, overfitting, and validation. Each study was appraised across four domains: participants, predictors, outcome, and analysis. Two reviewers conducted the evaluations independently, and any discrepancies were resolved by consensus.

## 3 Results

### 3.1 Literature search

The search strategy yielded a total of 4,531 records across three databases: PubMed/MEDLINE (n = 801), Scopus (n = 1,768), and Embase (n = 1,962). Following deduplication (n = 2,562), 1,969 unique records underwent title and abstract screening. Of these, 1,861 records were excluded because they were not original studies or were not relevant to the review topic, leaving 108 reports for retrieval. Full texts could not be retrieved for seven reports. Consequently, 101 full-text articles were assessed for eligibility, of which 58 were excluded, resulting in 43 studies included in the systematic review. The complete study selection process, including reasons for full-text exclusion, is presented in Fig 1.

**Fig 1:**
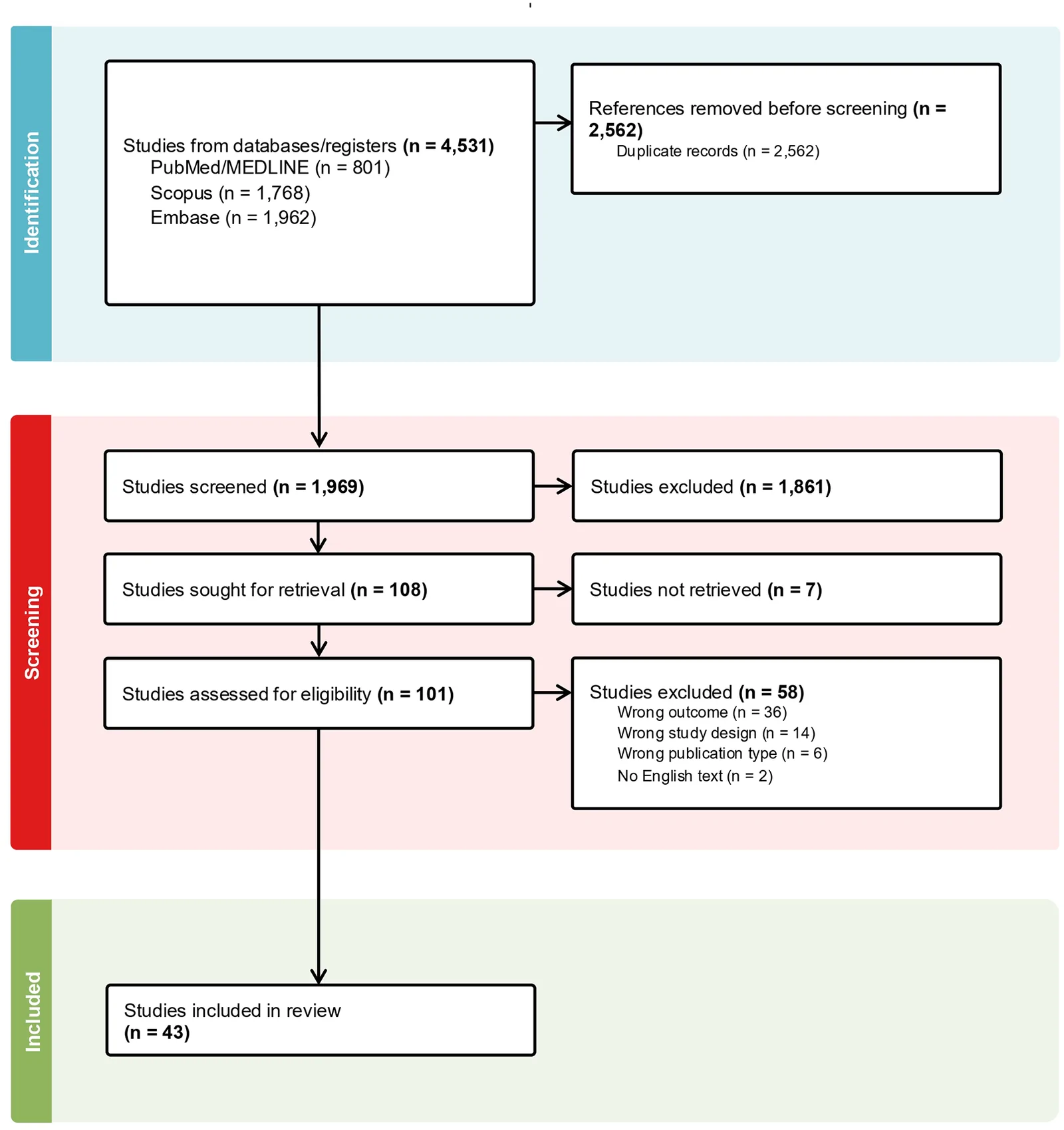
PRISMA flow diagram of study selection. Diagram showing the identification, screening, eligibility assessment, and inclusion of studies included in the systematic review and meta-analysis.

### 3.2 Temporal trends and geographic distribution of studies

The geographical distribution, shown in Fig 2, was highly concentrated in Asia. Overall, 35 of the 43 included studies (81.4%) involved Asian settings. Among single-country studies, India contributed the largest number (n = 12) [29–40], followed by Pakistan (n = 5) [41–45], Indonesia [46–48] with 4 studies, Thailand [49–51] with 3 studies, Bangladesh [52,53] and Yemen [54,55] with 2 studies, while Vietnam [56], Malaysia [57], Singapore [58], and Taiwan [59] with only 1 study. Three [60–62] studies covered multiple Asian countries within the same analysis.

**Fig 2:**
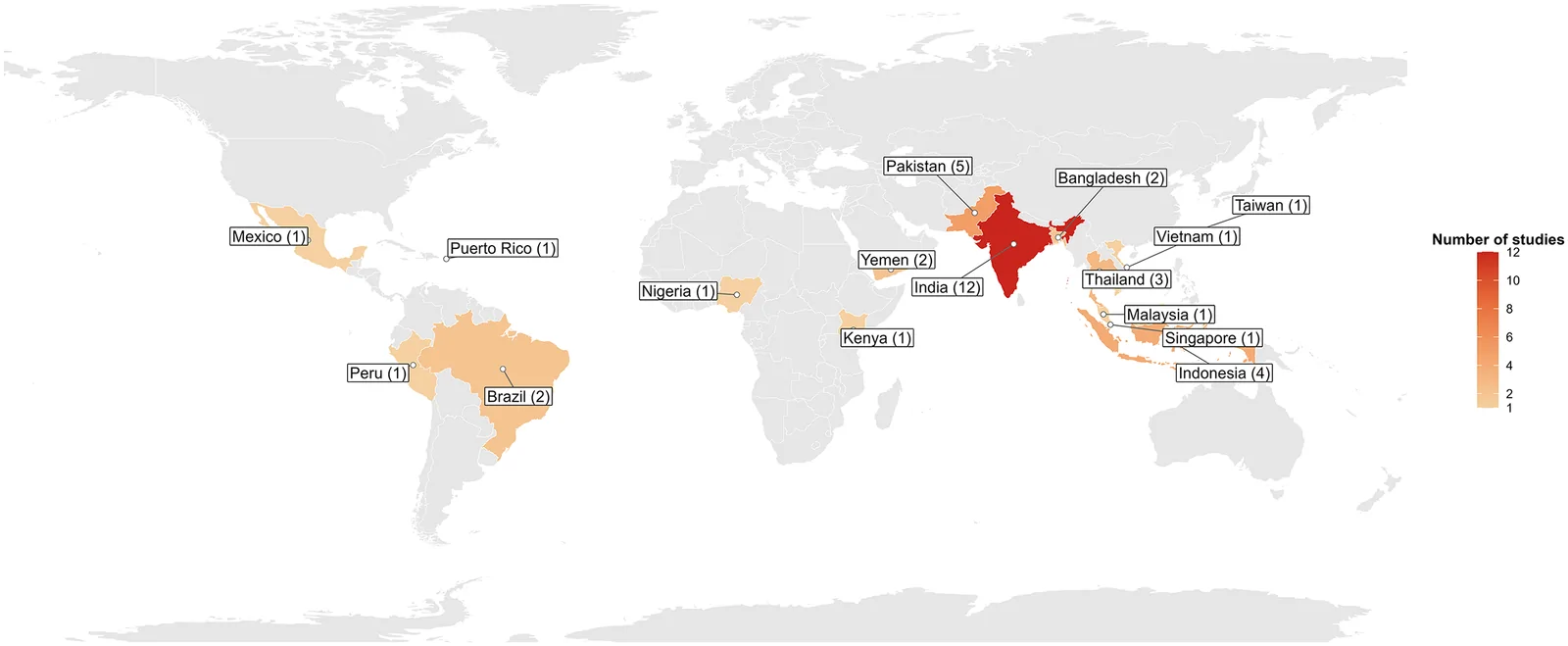
Geographical distribution of included studies by country. Countries are shaded according to the number of included single-country studies, and labels indicate the corresponding country-level study count. Four multi-country studies are not displayed as single-country counts. Base map: Natural Earth Admin 0 – Countries, 1:10m (public domain). Source: https://www.naturalearthdata.com/downloads/10m-cultural-vectors/10m-admin-0-countries/.

South America accounted for 3 studies (Brazil n = 2 [63,64]; Peru [65] n = 1), while North America contributed 2 studies (Mexico [66] and Puerto Rico [67] n = 1). Two studies were conducted in Africa (Nigeria [68] and Kenya [69] n = 1). Only 1 [70] study included a European setting, which involved only returning travellers and control samples, as part of a multi-country study combining Peru and Belgium. Overall, the country-level distribution shows a strong predominance of studies from South and Southeast Asia, with comparatively few studies from Africa, the Americas, or Europe.

The included studies were published between 2008 and 2025 (Table 1).

**Table 1:** Summary characteristics of the included studies.

First author (Year)	Continent	Country	Study design	Setting	Population	Disease	Case definition	Disease confirmation method
Abdualgalil, B. (2022)	Asia	Yemen	rDTA	Community-based (field / surveillance in population)	Pts (hospital/clinical cases)	Dengue	Pts with suspected infection	ELISA
Balamurugan, S.A.A. (2020)	Asia	India	MD	Hospital-based / Clinical	Pts (hospital/clinical cases)	Dengue	Pts at health centres with suspected DF, blood testing performed	Clinical + RT-PCR/ELISA
Balasaravanan, K. (2018)	Asia	India	MD	Hospital-based / Clinical	Pts (hospital/clinical cases)	Dengue	Pts with dengue-compatible symptoms (clinical documents)	Clinical + RT-PCR/ELISA
Bohm, B.C. (2024)	South America	Brazil	rDTA	National	Pts (hospital/clinical cases)	Dengue	Pts with suspected DF and acute fever + ≥2 symptoms (headache, retro-orbital pain, myalgia, arthralgia, rash)	Clinical + RT-PCR/ELISA
Bria, Y.P. (2024)	Asia	Indonesia	rDTA	Multicentre	Pts (hospital/clinical cases)	Dengue	Pts with DF or other febrile diseases (typhoid, malaria, COVID-19, etc.)	NA
Bria, Y.P. (2025)	Asia	Indonesia	rDTA	Multicentre	Pts (hospital/clinical cases)	Dengue	Pts with DF or other febrile diseases (typhoid, malaria, COVID-19, etc.)	NA
Chatterjee, S. (2018)	Asia	India	rDTA	Hospital-based / Clinical	Pts (hospital/clinical cases)	Dengue	Lab-confirmed DF	Lab-confirmed (RT-PCR/ELISA)
Daniels, B.C. (2024)	Asia	Thailand, Vietnam, Sri Lanka, Bangladesh	MD	Community-based (field / surveillance in population)	Children, adolescents	Dengue	DF suspected or identified by seroconversion / known infection	Lab-confirmed (RT-PCR/ELISA)
Das, S. (2025)	Asia	India	rDTA	Hospital-based / Clinical	Pts (hospital/clinical cases)	Dengue, Chikungunya	Pts with AFI	Clinical + RT-PCR/ELISA
Dasgupta, S. (2025)	Asia	India	rDTA	Hospital-based / Clinical	Pts (hospital/clinical cases)	Dengue	Pts with fever, headache, retro-orbital pain, malaise, or similar symptoms	Clinical + ELISA
Deepika, R. (2025)	Asia	India	MD	Hospital-based / Clinical	Pts (hospital/clinical cases)	Dengue	Pts with dengue symptoms (dataset)	Clinical + RT-PCR/ELISA
Falconi-Agapito, F. (2022)	South America, Europe	Peru, Belgium	DTA	Hospital-based / Clinical	Pts (hospital/clinical cases)	Dengue	AFI ≤ 7d with ≥1 dengue-suggestive symptom (Peru); travellers with suspected dengue (Belgium)	Lab-confirmed (RT-PCR/ELISA)
Goh, B. (2025)	Asia	Singapore	rDTA	Hospital-based / Clinical	Pts (hospital/clinical cases)	Dengue	Suspected DF (AFI)	Lab-confirmed (RT-PCR/ELISA)
Hasanah, I. (2020)	Asia	Indonesia	MD	Hospital-based / Clinical	Pts (hospital/clinical cases)	Dengue	Pts with dengue-like symptoms in medical records	Clinical + RT-PCR/ELISA
Hassan, M. (2022)	Asia	Pakistan	rDTA	Hospital-based / Clinical	Pts (hospital/clinical cases)	Dengue	Clinical DF diagnosis during outbreak (hospital-based)	Lab-confirmed (RT-PCR/ELISA)
Ho, T.S. (2020)	Asia	Taiwan	rDTA	Hospital-based / Clinical	GP	Dengue	Febrile pts with clinical suspicion of DF at ED	Lab-confirmed (RT-PCR/ELISA)
Hossain, M.S. (2019)	Asia	Bangladesh	MD	Hospital-based / Clinical	Pts (hospital/clinical cases)	Chikungunya	Pts with fever, arthralgia, headache, myalgia, or joint swelling	Lab-confirmed (RT-PCR/ELISA)
Husin, N.A. (2020)	Asia	Yemen	MD	Hospital-based / Clinical	Pts (hospital/clinical cases)	Dengue	Pts with dengue-like symptoms at hospital	Clinical (physician-based)
Ibrahim, F. (2010)	Asia	Malaysia	pMD	Hospital-based / Clinical	Pts (hospital/clinical cases)	Dengue	Clinical suspicion of DF with confirmatory IgM/IgG at admission	Lab-confirmed (RT-PCR/ELISA)
Jáuregui-Velarde, R. (2024)	South America	Peru	DTA	NA	Community (surveillance cohort / household level)	Dengue	NA	NA
Kaur, S. (2022)	Asia	India	rDTA	Hospital-based / Clinical	Pts (hospital/clinical cases)	Dengue	Pts with suspected DF (WHO dengue classification)	Clinical + ELISA
Khan, S. (2016)	Asia	Pakistan	DTA	Hospital-based / Clinical	Pts (hospital/clinical cases)	Dengue	Clinically suspected DF at admission (fever + typical symptoms)	Lab-confirmed (RT-PCR/ELISA)
Khan, S. (2017)	Asia	Pakistan	DTA	Hospital-based / Clinical	Pts (hospital/clinical cases)	Dengue	Clinical suspicion of DF requiring lab testing	Lab-confirmed (RT-PCR/ELISA)
Lu, B. (2025)	Asia	Vietnam, Indonesia, Philippines, Thailand, Malaysia	DTA	Hospital-based / Clinical	Pts (hospital/clinical cases)	Dengue	Pts with dengue-like symptoms	Clinical + RT-PCR/ELISA
Madewell, Z.J. (2024)	North America	Puerto Rico	MD	National	GP	Dengue	Suspected DF cases from surveillance system	Lab-confirmed (RT-PCR/ELISA)
Mayrose, H. (2023)a	Asia	India	DTA	Hospital-based / Clinical	Pts (hospital/clinical cases)	Dengue	NA	NA
Mayrose, H. (2023)b	Asia	India	rDTA	Hospital-based / Clinical	Pts (hospital/clinical cases)	Dengue	Clinical DF diagnosis (ICD coded)	Lab-confirmed (RT-PCR/ELISA)
Melo, C.F.O.R. (2018)	South America	Brazil	MD	Hospital-based / Clinical	Pts (hospital/clinical cases)	Zika	Pts with suspected ZIKV, serum analysed by HRMS	Lab-confirmed (RT-PCR/ELISA)
Ming, D.K. (2022)	Asia	Vietnam	pCoh	Community-based (field / surveillance in population)	Children	Dengue	AFI < 72h with clinical suspicion of DF	Lab-confirmed (RT-PCR/ELISA)
Obot, O. (2023)	Africa	Nigeria	rDTA	Hospital-based / Clinical	Pts (hospital/clinical cases)	Dengue	Clinically suspected DF	Lab-confirmed (RT-PCR/ELISA)
Puthenkaleekkal Thankappan, R. (2025)	Asia	India	rDTA	Hospital-based / Clinical	Pts (hospital/clinical cases)	Dengue	Pts with respiratory symptoms	Clinical + RT-PCR/ELISA
Qaiser, A. (2024)	Asia	Pakistan	rDTA	Hospital-based / Clinical	Pts (hospital/clinical cases)	Dengue	Pts with suspected DF, tested by NS1, IgM, IgG, RT-PCR	Clinical + RT-PCR/ELISA
Ramakrishna, M.M. (2023)	Asia	India	rDTA	Multicentre	Pts (hospital/clinical cases)	Dengue	NA	ELISA
Rao, V.S. (2012)	Asia	India	rDTA	Hospital-based / Clinical	Pts (hospital/clinical cases)	Dengue	Pts with suspected DF	Clinical + ELISA
Riya, N.J. (2024)	Asia	Bangladesh	rDTA	Multicentre	Pts (hospital/clinical cases)	Dengue	NA	NA
Ruiz Valdez, C.A. (2024)	North America	Mexico	DTA	Hospital-based / Clinical	GP	Dengue	Clinical suspicion of DF at first ED visit	Lab-confirmed (RT-PCR/ELISA)
Saleem, M. (2022)	Asia	Pakistan	DTA	Hospital-based / Clinical	Pts (hospital/clinical cases)	Dengue	Clinical DF diagnosis (NS1 and/or IgM–IgG positive)	Lab-confirmed (RT-PCR/ELISA)
Sa-ngamuang, C. (2018)	Asia	Thailand	pDTA	Hospital-based / Clinical	Adults, adolescents	Dengue	AFI < 14d with clinical suspicion of DF	Lab-confirmed (RT-PCR/ELISA)
Suhaeri, S. (2018)	Asia	Indonesia	MD	Community-based (field / surveillance in population)	Pts (hospital/clinical cases)	Dengue	Pts with suspected DF, haematological parameters available	Clinical + RT-PCR/ELISA
Tanner, L. (2008)	Asia	Vietnam, Singapore	pDTA	Multicentre	Pts (hospital/clinical cases)	Dengue	Pts with ≤72h onset of AFI, no alternative diagnosis	Clinical + RT-PCR/ELISA
Vu, D.M. (2023)	Africa	Kenya	rDTA	Multicentre	Children	Dengue	Pts aged 1–17y with AFI (T ≥ 38°C)	Clinical + RT-PCR/ELISA
Waiwijit, U. (2025)	Asia	Thailand	rDTA	Multicentre	Children	Dengue	Pts with AFI	Clinical + RT-PCR/ELISA
Williams, R.J. (2024)	Asia	Thailand	pDTA	Hospital-based / Clinical	GP	Dengue	AFI (T ≥ 38°C, ≤ 7d) with clinical suspicion of DF	Lab-confirmed (RT-PCR/ELISA)

AFI = Acute Febrile Illness; CHIKV = Chikungunya Virus; DENV = Dengue Virus; DF = Dengue Fever; DTA = Diagnostic Test Accuracy; ED = Emergency Department; ELISA = Enzyme-Linked Immunosorbent Assay; GP = General Population; HRMS = High-Resolution Mass Spectrometry; ICD = International Classification of Diseases; IgG = Immunoglobulin G; IgM = Immunoglobulin M; JEV = Japanese Encephalitis Virus; MD = Model Development; NA = Not available; NS1 = Non-structural Protein 1 antigen; pCoh = Prospective Cohort; pDTA = prospective Diagnostic Test Accuracy study; pMD = Prospective Model Development; PRNT = Plaque Reduction Neutralization Test; Pts = Patients; rDTA = retrospective Diagnostic Test Accuracy study; RT-PCR = Reverse Transcription Polymerase Chain Reaction; WHO = World Health Organization; ZIKV = Zika Virus; ZVD = Zika Virus Disease

### 3.3 Characteristics of the included studies

An overview of the general characteristics of the 43 included studies is presented in Table 1. Further methodological details are provided in S1 Appendix. The main patterns emerging from the extracted data are outlined in the following subsections.

#### 3.3.1 Study design and settings.

Most models were developed using retrospective diagnostic test accuracy (rDTA) designs (n = 20) [31–33,35,36,38–41,44,46,50,53,54,58,59,63,68,69,71] or modelling studies based on existing datasets (MD, n = 10) [29,30,34,47,48,52,55,60,64,67], while DTA designs without explicit retrospective/prospective specification were less frequent (n = 8) [37,42,43,45,61,65,66,70]. Only a minority adopted clearly prospective designs, including 3 [49,51,62] prospective diagnostic accuracy studies (pDTA), only one [56] prospective cohort studies (pCoh), and another one prospective modelling study (pMD) [57]. The majority of studies regarded hospital or clinical settings (n = 29) [29–38,40–45,47,49,51,52,55,57–59,61,64,66,68,70], while additional data were derived from multicentre clinical networks (n = 7) [39,46,50,53,62,69,71] and community-based or field surveillance cohorts (n = 4) [48,54,56,60]. Two studies [63,67] used national-level surveillance data; one [65] study did not clearly report the setting.

#### 3.3.2 Study populations and diseases.

Most studies were based on individual patients presenting as hospital or clinical cases (n = 33) [29–48, 52–55,57,58,61–64,68,70,71], while 4 [51,59,66,67] studies concerned general population samples, and 5 [49,50,56,60,69] specifically targeted paediatric or adolescent populations (children or children/adolescents). One [65] study used a community household surveillance cohort.

Dengue overwhelmingly dominated the evidence base, being evaluated in 41 of the 43 included studies (93%) [29–31, 33–51, 53–63,65–71]. One [32] study jointly modelled dengue and chikungunya and/or zika virus infection, while one [64] focused exclusively on zika and one [52] exclusively on chikungunya.

#### 3.3.3 Case definition and disease confirmation method.

Case definitions were primarily based on acute febrile illness or clinically suspected dengue, defined as fever within a predefined time window accompanied by compatible clinical symptoms, with some studies applying WHO dengue classification criteria. In total, 37 studies [29–36,38–45,47–52,54,56–64,66–70] incorporated laboratory confirmation as part of the reference standard, most frequently RT-PCR and/or ELISA alone or combined with clinical criteria, whereas a small number relied mainly on clinical or physician-based diagnoses. All models were developed at the individual patient level; no study used aggregated (e.g., facility- or district-level) outcomes.

#### 3.3.4 Data structure, validation, calibration, and implementation readiness.

For most studies (n = 41) [30–33,35–71], predictors were restricted to information available at the time of clinical assessment or diagnosis, while two [29,34] models explicitly incorporated follow-up data. Handling of missing data and class imbalance was heterogeneous and often poorly reported: 22 studies [31–34,36–39,42,43,45,47,49,50,55,58,60,63–65,67,71] did not describe any specific strategy. Among those that did, approaches ranged from case-wise exclusion of incomplete records to single or multiple imputation, including median imputation for laboratory variables, belief-based updating schemes, and random forest-based internal imputation.

Internal validation was commonly performed using k-fold cross-validation (most frequently 10-fold) and/or random hold-out splits, typically 70/30 or 80/20 train-test partitions; some studies combined both approaches. Prospective or external validation was rare, being limited to a few studies [61,66] that used distinct temporal datasets or cross-dataset evaluation. Calibration was reported infrequently: only 4 [51,56,59,61] studies presented formal or semi-formal calibration assessments, using Hosmer–Lemeshow tests, Brier scores, or calibration plots. Finally, implementation status was uniformly early-stage: 40 studies [29–51,53–64,67–71] were classified as research-only prototypes, while the remaining 3 [52,65,66] explicitly classified their work as pilot or proof-of-concept models; no study reported routine clinical deployment.

### 3.4 Predictor variables

As shown in Fig 3, laboratory parameters (n = 32) [29–36,38–45,47–50,52,53,56,58–62,64,67,68,70] and clinical variables (n = 26) [29,30,34,35,39,40,46–49,51,52,54–59,62,63,65–69,71] were the most frequently used predictors across the included studies, followed by socio-demographic variables (n = 24) [29,33,35,39,44,46,47,49,51,54,56–64,66–69,71] and biomarker data (n = 13) [29–31,34,41–43,45,48,57,64,67,70]. Epidemiological or surveillance data (n = 5) [29,49,51,66,67], and genomic or virological markers (n = 3) [31,64,67] were rarely included. Environmental data (n = 1) [51] were only sporadically used. Notably, none of the included diagnostic-level ML studies incorporated entomological data.

**Fig 3:**
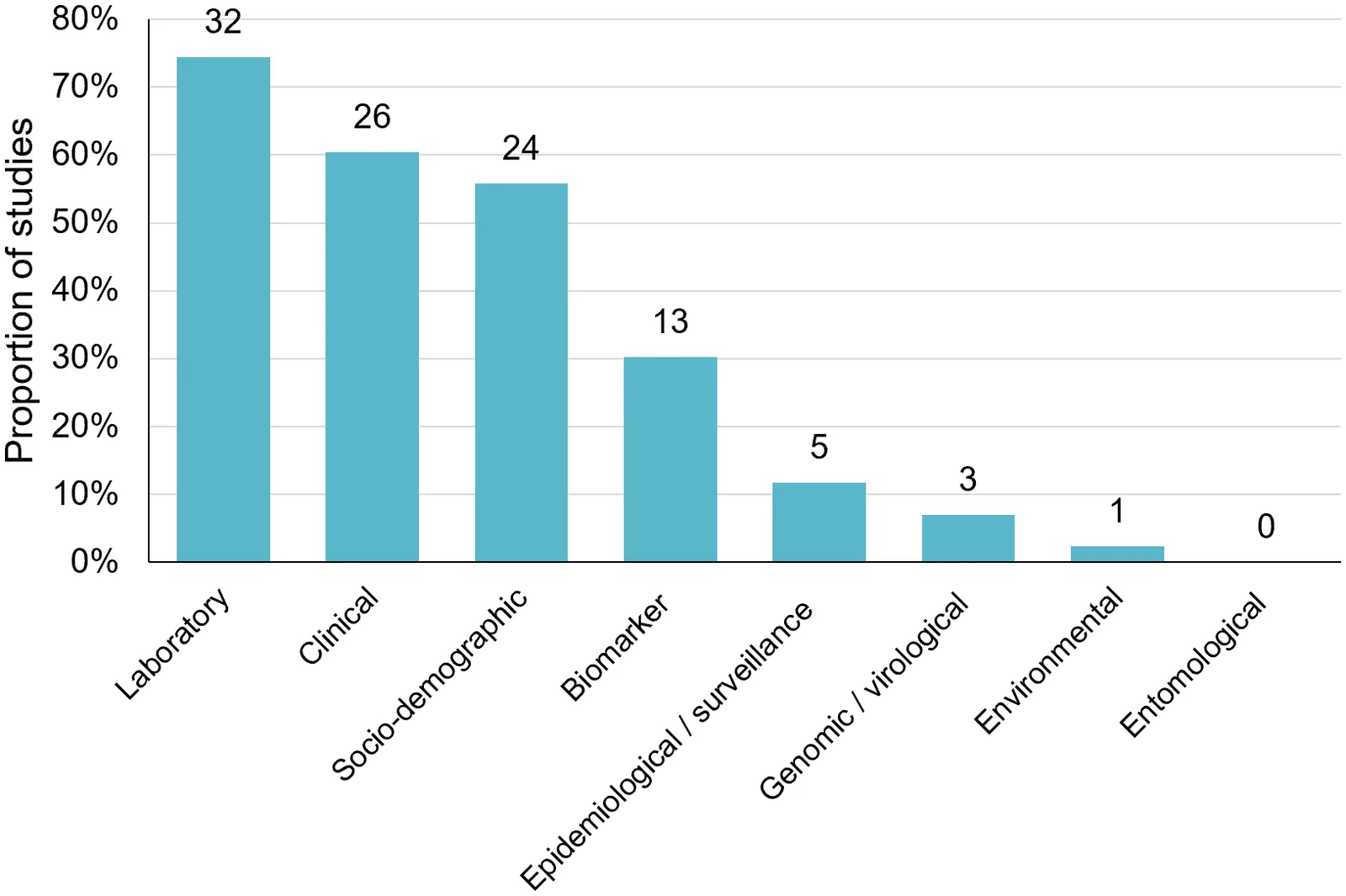
Distribution of predictor feature categories across included diagnostic machine-learning studies. Bar chart showing the frequency of predictor feature categories used in machine-learning models for the diagnosis of mosquito-borne viral infections across the included studies.

Fig 4 provides a conceptual synthesis of the main categories of input data used for AI/ML-based diagnosis, illustrating representative examples for each input type.

**Fig 4:**
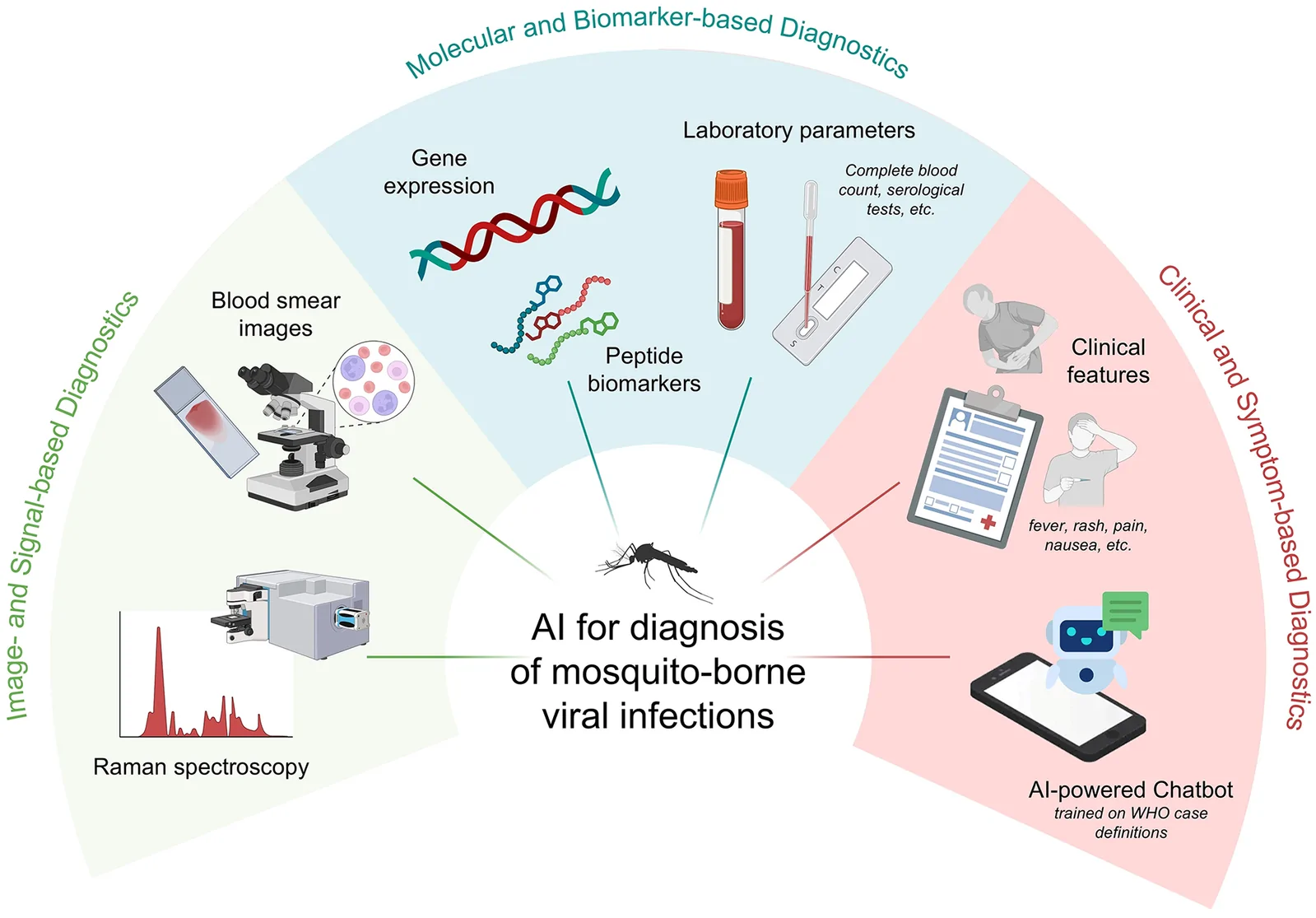
Conceptual overview of input data types used in AI/ML diagnostic models for mosquito-borne viral infections. Schematic representation of the main categories of input data used in artificial intelligence and machine-learning diagnostic models, based on representative examples from selected studies included in the systematic review [31,33,36,38,41,44,50,53,58,64,67,69,70] (figure created with BioRender.com).

### 3.5 Classification metrics results

Across the included studies, AI methods varied widely in complexity and included classical machine-learning algorithms, tree-ensemble methods, deep-learning architectures, and heuristic approaches. Several studies evaluated multiple standalone algorithms belonging to more than one methodological family and were therefore labelled as “Multiple AI/ML approaches” in Table 2. In the following descriptive subsections, however, performance is summarised at the individual model level according to the same methodological families used in the quantitative analyses. Consequently, models originating from studies comparing multiple AI/ML approaches may contribute to more than one family. Reported performance varied substantially across models and datasets (Table 2).

**Table 2:** Results of classification metrics for the AI models used in the included studies.

First author (Year)	AI Models	AI Category	Sample size train/test/validation	AUC	Sensitivity	Specificity	PPV / precision	NPV	Accuracy	F1-score
Abdualgalil, B. (2022)	KNN; GBC; ETC; XGB; LightGBM	Multiple AI/ML approaches	6694	KNN: 0.99; GBC: 0.98; XGB: 0.99; ETC: 0.99; LightGBM: 0.99	KNN: 0.99; GBC: 0.97; XGB: 0.98; ETC: 0.99; LightGBM: 0.99	NA	KNN: 0.98; GBC: 0.98; XGB: 0.99; ETC: 0.99; LightGBM: 0.99	NA	KNN: 0.99; GBC: 0.98; XGB: 0.99; ETC: 0.99; LightGBM: 0.99	KNN: 0.99; GBC: 0.98; XGB: 0.99; ETC: 0.99; LightGBM: 0.99
Balamurugan, S.A.A. (2020)	EWSORA + NB; J48; MLP; SVM	Multiple AI/ML approaches	480	NB: 0.89; J48: 0.89; MLP: 0.89; SVM: 0.89	NB: 0.95; J48: 0.95; MLP: 0.95; SVM: 0.95	NA	NB: 0.93; J48: 0.93; MLP: 0.93; SVM: 0.93	NA	NB: 0.96; J48: 0.99; MLP: 0.99; SVM: 0.95	NB: 0.94; J48: 0.94; MLP: 0.94; SVM: 0.94
Balasaravanan, K. (2018)	MLP	Deep Learning	4500	NA	0.89	NA	NA	NA	0.95	NA
Bohm, B.C. (2024)	DT; KNN; MLP	Multiple AI/ML approaches	20,000 (train: 14,000; test: 6,000)	DT: 0.99; KNN: 0.97; MLP: 0.99	NA	NA	NA	NA	DT: 0.93; KNN: 0.92; MLP: 0.93	DT: 0.93; KNN: 0.93; MLP: 0.93
Bria, Y.P. (2024)	LDA; KNN; CART; XGB; RF; NB; SVM	Multiple AI/ML approaches	439	NA	NA	NA	NA	NA	LDA: 0.92; KNN: 0.93; CART: 0.90; XGB: 0.93; RF: 0.95; NB: 0.95; SVM: 0.90	LDA: 0.75; KNN: 0.80; CART: 0.70; XGB: 0.79; RF: 0.86; NB: 0.86; SVM: 0.76
Bria, Y.P. (2025)	KNN; XGB; RF; NB; SVM	Multiple AI/ML approaches	561	NA	NA	NA	KNN: 0.67; XGB: 0.77; RF: 0.90; NB: 0.66; SVM: 0.66	NA	LR: 0.90; KNN: 0.89; XGB: 0.91; RF: 0.93; NB: 0.88; SVM: 0.89	NA
Chatterjee, S. (2018)	ANN	Deep Learning	56	NA	0.99	0.97	0.97	0.98	0.98	0.98
Daniels, B.C. (2024)	GBM; RF; ANN; SVM	Multiple AI/ML approaches	NA	MLR: 0.66–0.79; RF: 0.74–0.85; GBM: 0.76–0.85; ANN: 0.73–0.84; SVM: 0.72–0.83	MLR: 0.65 (95% CI: 0.60–0.70); RF: 0.72 (95% CI: 0.67–0.77); GBM: 0.71 (95% CI: 0.66–0.76); ANN: 0.71 (95% CI: 0.66–0.75); SVM: 0.70 (95% CI: 0.65–0.74)	MLR: 0.66 (95% CI: 0.62–0.70); RF: 0.74 (95% CI: 0.70–0.78); GBM: 0.75 (95% CI: 0.70–0.79); ANN: 0.73 (95% CI: 0.69–0.77); SVM: 0.72 (95% CI: 0.68–0.76)	Scenario A: MLR 0.69; RF 0.74; GBM 0.75; ANN 0.73; SVM 0.72; Scenario B: MLR 0.71; RF 0.76; GBM 0.76; ANN 0.74; SVM 0.73; Scenario C: MLR 0.68; RF 0.72; GBM 0.73; ANN 0.72; SVM 0.71; Scenario D: MLR 0.69; RF 0.75; GBM 0.75; ANN 0.73; SVM 0.72	Scenario A: MLR 0.66; RF 0.73; GBM 0.74; ANN 0.72; SVM 0.71; Scenario B: MLR 0.68; RF 0.74; GBM 0.75; ANN 0.73; SVM 0.72; Scenario C: MLR 0.65; RF 0.72; GBM 0.73; ANN 0.71; SVM 0.70; Scenario D: MLR 0.66; RF 0.73; GBM 0.74; ANN 0.72; SVM 0.71	Scenario A: MLR 0.66; RF 0.71; GBM 0.72; ANN 0.70; SVM 0.70; Scenario B: MLR 0.68; RF 0.73; GBM 0.72; ANN 0.71; SVM 0.70; Scenario C: MLR 0.65; RF 0.70; GBM 0.71; ANN 0.70; SVM 0.69; Scenario D: MLR 0.66; RF 0.72; GBM 0.72; ANN 0.71; SVM 0.70	NA
Das, S. (2025)	SVM; ANN; RF	Multiple AI/ML approaches	302 (train: 212; test: 90)	SVM: 1.00; ANN: 1.00; RF: 1.00	SVM: 0.99; ANN: 1.00; RF: 1.00	NA	SVM: 0.99; ANN: 1.00; RF: 1.00	NA	SVM: 0.99; ANN: 1.00; RF: 1.00	SVM: 0.99; ANN: 1.00; RF: 1.00
Dasgupta, S. (2025)	ANN; MARS	Multiple AI/ML approaches	120 (train: 96; test: 24)	NA	NA	NA	NA	NA	ANN: 0.96; MARS: 0.88	NA
Deepika, R. (2025)	NB	Classical ML	108	1.00	1.00	NA	1.00	NA	1.00	NA
Falconi-Agapito, F. (2022)	RF	Tree Ensemble	323	Single peptide (FPG-1): 0.81	RFM3: 0.72 (95% CI: 0.64–0.79); RFG1: 0.89 (95% CI: 0.78–0.95); RFG2: 0.89 (95% CI: 0.77–0.95); RFG3: 0.88 (95% CI: 0.82–0.93)	RF models: ≥ 0.80	RFM3: 0.40; RFG3: 0.44	RFM3: 0.95; RFG3: 0.98	NA	NA
Goh, B. (2025)	RF; GBM; AdaBoost; SVM; KNN; NB	Multiple AI/ML approaches	4,225 (train: 2,957; test: 1,268)	RF: 0.88; GB: 0.87; AdaBoost: 0.86; NB: 0.80; KNN: 0.82; SVM: 0.85	RF: 0.82; GBM: 0.80; AdaBoost: 0.78; NB: 0.71; KNN: 0.76; SVM: 0.78	RF: 0.84; GBM: 0.83; AdaBoost: 0.82; NB: 0.77; KNN: 0.80; SVM: 0.82	RF: 0.81; GBM: 0.79; AdaBoost: 0.77; NB: 0.69; KNN: 0.74; SVM: 0.77	RF: 0.86; GBM: 0.85; AdaBoost: 0.83; NB: 0.78; KNN: 0.81; SVM: 0.83	RF: 0.83; GBM: 0.82; AdaBoost: 0.80; NB: 0.75; KNN: 0.78; SVM: 0.80	RF: 0.81; GBM: 0.79; AdaBoost: 0.77; NB: 0.70; KNN: 0.75; SVM: 0.77
Hasanah, I. (2020)	DT	Classical ML	Train: 100; test: 20	NA	NA	NA	NA	NA	0.95	NA
Hassan, M. (2022)	DENV-TLDNN (CNN; ResNet101; transfer learning)	Deep Learning	100 (train: 67; test: 33)	0.96	0.97	0.95	0.96	NA	0.96	0.97
Ho, T.S. (2020)	DNN; DT	Multiple AI/ML approaches	4,894 (train: 3,425; test: 1,469)	DNN: 0.96 (95% CI: 0.95–0.97); DT: 0.95 (95% CI: 0.94–0.96)	DNN: 0.93; DT: 0.91	DNN: 0.89; DT: 0.87	DNN: 0.90; DT: 0.88	DNN: 0.92; DT: 0.90	DNN: 0.91; DT: 0.89	DNN: 0.91; DT: 0.89
Hossain, M.S. (2019)	BRBES (Evidential Reasoning)	Heuristic	250	0.92 (95% CI: 0.83–0.96)	NA	NA	NA	NA	NA	NA
Husin, N.A. (2020)	MLP	Deep Learning	1,054 (train: 736; val: 158; test: 160)	NA	NA	NA	NA	NA	1.00	NA
Ibrahim, F. (2010)	MFNN	Deep Learning	165 (train: 115; test: 50)	NA	0.96	0.90	0.92	0.95	0.93	0.94
Jáuregui-Velarde, R. (2024)	GPT-3.5	Deep Learning	30	NA	1.00	1.00	NA	NA	1.00	NA
Kaur, S. (2022)	RF	Tree Ensemble	9,000 (train: 2,000; test: 2,000)	NA	0.90	1.00	1.00	NA	0.94	0.94
Khan, S. (2016)	SVM (Poly, RBF, Linear)	Classical ML	84 sera (31 dengue + ; 53 negative)	NA	Poly1: 0.73; Poly2: 0.73; RBF σ1: 0.71; RBF σ2: 0.72; Linear: 0.71	Poly1: 0.93; Poly2: 0.86; RBF σ1: 0.87; RBF σ2: 0.89; Linear: 0.91	Poly1: 0.90; Poly2: 0.77; RBF σ1: 0.81; RBF σ2: 0.84; Linear: 0.87	NA	Poly2: 0.81; RBF σ1: 0.81; RBF σ2: 0.82; Linear: 0.82	Poly1: 0.81; Poly2: 0.75; RBF σ1: 0.76; RBF σ2: 0.78; Linear: 0.78
Khan, S. (2017)	RF	Tree Ensemble	100 sera (45 positive; 55 negative)	NA	RF (full dataset): 0.98; RF (test folds): 0.91	RF (full dataset): 0.97; RF (test folds): 0.91	NA	NA	RF (full dataset): 0.97; RF (test folds): 0.91	NA
Lu, B. (2025)	CART; SVM	Multiple AI/ML approaches	Dataset 1: 5,726; Dataset 2: 1,486; Dataset 3: 1,573; Dataset 4: 257; Dataset 5: 368	0.60–0.90	0.10–1.00	0.10–1.00	NA	NA	NA	NA
Madewell, Z.J. (2024)	SVM; RF; ANN; AdaBoost; LightGBM; XGB	Multiple AI/ML approaches	NA	SVM: 0.74–0.83; RF: 0.76–0.84; ANN: 0.76–0.86; AdaBoost: 0.75–0.84; LightGBM: 0.76–0.85; XGB: 0.77–0.85	SVM: 0.86; RF: 0.87; ANN: 0.89; AdaBoost: 0.86; LightGBM: 0.92; XGB: 0.87	SVM: 0.85; RF: 0.86; ANN: 0.82; AdaBoost: 0.89; LightGBM: 0.83; XGB: 0.90	SVM: 0.86; RF: 0.87; ANN: 0.88; AdaBoost: 0.86; LightGBM: 0.91; XGB: 0.87	SVM: 0.86; RF: 0.87; ANN: 0.86; AdaBoost: 0.87; LightGBM: 0.88; XGB: 0.88	SVM: 0.85; RF: 0.87; ANN: 0.85; AdaBoost: 0.87; LightGBM: 0.88; XGB: 0.88	SVM: 0.85; RF: 0.86; ANN: 0.83; AdaBoost: 0.88; LightGBM: 0.85; XGB: 0.89
Mayrose, H. (2023)a	MobileNetV2 (pre-trained) + SVM	Deep Learning	94	NA	0.98	0.93	NA	NA	0.96	NA
Mayrose, H. (2023)b	DT; LDA; NB; SVM; KNN; MLP	Multiple AI/ML approaches	94 (54 dengue; 40 controls)	Morphological/GLSDM: SVM 0.96; DT 0.96; LDA 0.96; NB 0.97; KNN 0.88; MLP 0.94; Deep/LBP: SVM 0.98; MLP 0.96; LDA 0.93; NB 0.94; KNN 0.92; DT 0.80	Morphological/GLSDM: SVM 0.93; DT 0.93; LDA 0.94; NB 0.78; KNN 0.85; MLP 0.96; Deep/LBP: SVM 0.98; MLP 0.94; LDA 0.96; NB 0.93; KNN 0.91; DT 0.78	Morphological/GLSDM: SVM 0.95; DT 0.95; LDA 0.90; NB 0.98; KNN 0.90; MLP 0.88; Deep/LBP: SVM 0.93; MLP 0.95; LDA 0.90; NB 0.83; KNN 0.93; DT 0.73	Morphological/GLSDM: SVM 0.96; DT 0.96; LDA 0.93; NB 0.98; KNN 0.92; MLP 0.91; Deep/LBP: SVM 0.95; MLP 0.96; LDA 0.93; NB 0.88; KNN 0.94; DT 0.79	NA	Morphological/GLSDM: SVM 0.94; DT 0.94; LDA 0.93; NB 0.86; KNN 0.87; MLP 0.93; Deep/LBP: SVM 0.96; MLP 0.95; LDA 0.94; NB 0.88; KNN 0.91; DT 0.76	Morphological/GLSDM: SVM 0.94; DT 0.94; LDA 0.94; NB 0.87; KNN 0.88; MLP 0.94; Deep/LBP: SVM 0.96; MLP 0.95; LDA 0.95; NB 0.90; KNN 0.92; DT 0.73
Melo, C.F.O.R. (2018)	RF	Tree Ensemble	585 (test: 39)	NA	0.93 (95% CI: 0.70–0.99)	0.96 (95% CI: 0.80–0.99)	0.93 (95% CI: 0.70–0.99)	0.96 (95% CI: 0.80–0.99)	0.95 (95% CI: 0.83–0.99)	0.93 (95% CI: 0.82–1.00)
Ming, D.K. (2022)	XGB	Tree Ensemble	6,480 (train: 4,688; hold-out: 1,792)	CV median AUC: 0.86; hold-out: 0.86	CV: 0.56; Hold-out: 0.79	CV: 0.92; Hold-out: 0.78	CV: 0.73; Hold-out: 0.58	CV: 0.84; Hold-out: 0.91	NA	NA
Obot, O. (2023)	RF; XGB; LightGBM; ANN	Multiple AI/ML approaches	820 (train: 656; test: 164)	RF: 0.92; LightGBM: 0.94; XGB: 0.95; ANN: 0.91	RF: 0.87; LightGBM: 0.89; XGB: 0.90; ANN: 0.85	RF: 0.88; LightGBM: 0.90; XGB: 0.91; ANN: 0.87	RF: 0.86; LightGBM: 0.89; XGB: 0.90; ANN: 0.84	RF: 0.89; LightGBM: 0.91; XGB: 0.92; ANN: 0.88	RF: 0.88; LightGBM: 0.90; XGB: 0.91; ANN: 0.87	RF: 0.86; LightGBM: 0.89; XGB: 0.90; ANN: 0.84
Puthenkaleekkal Thankappan, R. (2025)	GAN	Deep Learning	208 (train: 139; test: 69)	NA	1.00	NA	1.00	NA	NA	1.00
Qaiser, A. (2024)	LR; XGB; LightGBM; RF; SVM (RBF); CatBoost	Multiple AI/ML approaches	300 (train: 210; test: 90; synthetic external validation: 200)	LR: 0.58; XGB: 0.43; LightGBM: 0.54; RF: 0.50; SVM (RBF): 0.64; CatBoost: 0.51	LR: 0.97; XGB: 0.68; LightGBM: 0.86; RF: 0.68; SVM (RBF): 0.97; CatBoost: 0.88	NA	LR: 0.70; XGB: 0.63; LightGBM: 0.72; RF: 0.65; SVM (RBF): 0.72; CatBoost: 0.69	NA	LR: 0.70; XGB: 0.50; LightGBM: 0.66; RF: 0.52; SVM (RBF): 0.71; CatBoost: 0.64	NA
Ramakrishna, M.M. (2023)	ChOA-ANN; PSO-ANN; GA-ANN; ANN; UChOA-ANN	Deep Learning	110 (train: 77; test: 33)	NA	ChOA-ANN: 0.88; PSO-ANN: 0.88; GA-ANN: 0.80; ANN: 0.98; UChOA-ANN: 0.99	ChOA-ANN: 0.92; PSO-ANN: 0.88; GA-ANN: 0.88; ANN: 0.51; UChOA-ANN: 0.96	ChOA-ANN: 0.92; PSO-ANN: 0.88; GA-ANN: 0.87; ANN: 0.79; UChOA-ANN: 0.96	ChOA-ANN: 0.88; PSO-ANN: 0.88; GA-ANN: 0.81; ANN: 0.77; UChOA-ANN: 0.92	ChOA-ANN: 0.90; PSO-ANN: 0.88; GA-ANN: 0.84; ANN: 0.86; UChOA-ANN: 0.94	NA
Rao, V.S. (2012)	ADT; DT; SVM	Multiple AI/ML approaches	Dataset 1–2: 646; Dataset 3–4: 398	ADT: 0.93–1.00; DT: 0.84–1.00; SVM: 0.75–0.99	ADT: 0.89–1.00; DT: 0.87–0.96; SVM: 0.89–0.99	ADT: 0.83–1.00; C4.5: 0.75–0.97; SVM: 0.61–1.00	NA	NA	ADT: 0.87–1.00; C4.5: 0.82–0.96; SVM: 0.78–0.99	NA
Riya, N.J. (2024)	SVM; NB; RF; AdaBoost; XGB; MLP; LightGBM; ANN; CNN; GRU; Bi-LSTM; TabPFN; TabTransformer	Multiple AI/ML approaches	320	Stacking classifier: 0.99	SVM: 0.91; NB: 0.80; RF: 0.90; AdaBoost: 0.87; XGB: 0.93; MLP: 0.83; LightGBM: 0.91; Stacking: 0.92; ANN: 0.78; CNN: 0.79; GRU: 0.78; Bi-LSTM: 0.84; TabPFN: 0.95; TabTransformer: 0.88	NA	SVM: 0.89; NB: 0.79; RF: 0.89; AdaBoost: 0.87; XGB: 0.91; MLP: 0.86; LightGBM: 0.92; Stacking: 0.94; ANN: 0.77; CNN: 0.80; GRU: 0.80; Bi-LSTM: 0.81; TabPFN: 0.94; TabTransformer: 0.88	NA	SVM: 0.91; NB: 0.81; RF: 0.91; AdaBoost: 0.89; XGB: 0.93; MLP: 0.83; LightGBM: 0.92; Stacking: 0.94; ANN: 0.79; CNN: 0.81; GRU: 0.81; Bi-LSTM: 0.81; TabPFN: 0.95; TabTransformer: 0.90	SVM: 0.90; NB: 0.79; RF: 0.90; AdaBoost: 0.87; XGB: 0.92; MLP: 0.83; LightGBM: 0.91; Stacking: 0.93; ANN: 0.77; CNN: 0.79; GRU: 0.79; Bi-LSTM: 0.81; TabPFN: 0.94; TabTransformer: 0.88
Ruiz Valdez, C.A. (2024)	ANN	Deep Learning	466 (233 positive; 233 negative)	0.89 (95% CI: 0.86–0.92)	0.87	0.85	0.86	0.86	0.86	0.86
Saleem, M. (2022)	PCA-SVM (RBF, Linear, Poly); PCA-DA; PLS-DA; PLSR	Multiple AI/ML approaches	52 DENV (train/val) + 30 healthy (test); total 1,240 spectra	NA	PCA-SVMPoly-5 (4 PCs): 0.99; PCA-SVMRBF (7 PCs): 1.00; PLS-DA: 0.94; PLSR: 0.95; PCA-DA: 0.74	PCA-SVMPoly-5: 0.99; PCA-SVMRBF: 1.00; PLS-DA: 0.98; PLSR: 0.92; PCA-DA: 0.90	NA	NA	PCA-SVMPoly-5: 1.00; PCA-SVMRBF: 1.00; PLS-DA: 0.97; PLSR: 0.94; PCA-DA: 0.84	NA
Sa-ngamuang, C. (2018)	NB	Classical ML	397 (260 dengue; 137 non-dengue)	NB without NS1: 0.95 (95% CI: 0.92–0.97); NB with NS1: 0.97 (95% CI: 0.95–0.98)	NB without NS1: 0.89 (95% CI: 0.85–0.93); NB with NS1: 0.93 (95% CI: 0.89–0.96)	NB without NS1: 0.91 (95% CI: 0.85–0.95); NB with NS1: 0.95 (95% CI: 0.90–0.98)	NB without NS1: 0.93; NB with NS1: 0.96	NB without NS1: 0.85; NB with NS1: 0.90	NB without NS1: 0.90; NB with NS1: 0.94	NB without NS1: 0.91; NB with NS1: 0.94
Suhaeri, S. (2018)	ANN	Deep Learning	98	NA	NA	NA	NA	NA	0.97	NA
Tanner, L. (2008)	DT	Classical ML	1200	0.88	0.71	0.90	NA	NA	0.84	NA
Vu, D.M. (2023)	Boosted LR; DT; RF; SVM; NB; MLP	Multiple AI/ML approaches	6,208 (train: 4,347; test: 1,861)	NA	Boosted LR: 0.06; DT: 0.00; RF: 0.00; SVM: 0.00; NB: 0.00; MLP: 0.00	Boosted LR: 0.98; DT: 1.00; RF: 1.00; SVM: 1.00; NB: 1.00; MLP: 1.00	NA	Boosted LR: 0.92; DT: 0.92; RF: 0.92; SVM: 0.92; NB: 0.92; MLP: 0.92	Boosted LR: 0.91; DT: 0.92; RF: 0.92; SVM: 0.92; NB: 0.92; MLP: 0.92	NA
Waiwijit, U. (2025)	LDA; PCA-LDA; MLP; SVM; DT; RF	Multiple AI/ML approaches	60 (49 spectra per patient)	LDA: 0.99	LDA: 0.95; PCA-LDA: 0.93; MLP: 0.93; SVM: 0.93; DT: 0.90; RF: 0.97	NA	LDA: 1.00; PCA-LDA: 0.93; MLP: 0.90; SVM: 0.88; DT: 0.88; RF: 0.76	NA	LDA: 0.97; PCA-LDA: 0.90; MLP: 0.88; SVM: 0.87; DT: 0.85; RF: 0.78	LDA: 0.97; PCA-LDA: 0.93; MLP: 0.91; SVM: 0.93; DT: 0.90; RF: 0.97
Williams, R.J. (2024)	RF	Tree Ensemble	12,833 (5,731 RT-PCR positive)	0.87 (95% CI: 0.86–0.88)	0.82	0.79	0.74	0.85	0.80	0.78

ADT = Alternative Decision Tree; ANN = Artificial Neural Network; ANN-MCS = ANN with Modified Cuckoo Search; Bi-LSTM = Bidirectional Long Short-Term Memory; BRBES = Belief Rule Based Expert System; CART = Classification and Regression Tree; CatBoost = Categorical Boosting; ChOA/PSO/GA = Chimp Optimization Algorithm/Particle Swarm Optimization/Genetic Algorithm; CNN = Convolutional Neural Network; DENV-TLDNN = Dengue Virus Transfer Learning Deep Neural Network; DNN = Deep Neural Network; DT = Decision Tree; ElasticNet = Elastic Net Regression; ELM = Extreme Learning Machine; EWSORA = Entropy Weighted Score-based Optimal Ranking Algorithm; ETC = Extra Trees Classifier; GAN = Generative Adversarial Network; GBC = Gradient Boosting Classifier; GBM = Gradient Boosting Machine; GPT-3.5 = Generative Pretrained Transformer 3.5; GRU = Gated Recurrent Unit; KNN = K-Nearest Neighbor; LDA = Linear Discriminant Analysis; LightGBM = Light Gradient Boosting Machine; LR = Logistic Regression; MARS = Multivariate Adaptive Regression Splines; MFNN = Multilayer Feed-Forward Neural Network; MLP = Multilayer Perceptron; NA = Not available; NB = Naïve Bayes; PAHO = Pan American Health Organization; PCA = Principal Component Analysis; PCA-LDA = Principal Component Analysis–Linear Discriminant Analysis; PLS/PLSR/PLS-DA = Partial Least Squares/Partial Least Squares Regression/Partial Least Squares Discriminant Analysis; Poly = Polynomial kernel; RBF = Radial Basis Function; ResNet = Residual Neural Network; RF = Random Forest; SVM = Support Vector Machine; TabPFN = Tabular Prior-Data Fitted Network; UChOA-ANN = Updated Chimp Optimization Algorithm–Artificial Neural Network; XGB = eXtreme Gradient Boosting.

#### 3.5.1 Classical ML.

Classical ML models included decision-tree methods, SVMs, Bayesian networks, KNN, LDA, Naive Bayes, logistic-regression approaches, MARS, stacking, and dimensionality-reduction/classification pipelines. Reported model-level performance varied substantially across studies. AUC values ranged from 0.58 in the logistic-regression model [44] to 1.00 [32,34]. Sensitivity ranged from 0.00 [69] to 1.00 [34,45]. Specificity ranged from 0.59 in the Naive Bayes model reported by Riya et al. [53] to 1.00 [45,50]. PPV ranged from 0.22 in the logistic-regression model [69] to 1.00 [34,45,50]. NPV ranged from 0.59 [53] to 1.00 [45]. Accuracy ranged from 0.65 in an MLR scenario [60] to 1.00 [34,45]. Reported F1-scores ranged from 0.70 [58] to 0.99 [32,54]. The broad ranges reflect marked variation in populations, class balance, predictors, validation strategies, and individual algorithm behaviour (Table 2).

#### 3.5.2 Tree ensemble.

Tree-ensemble models included RF, GBM/GBC, XGBoost, LightGBM, AdaBoost, CatBoost, ETC, and CART. Reported model-level performance was highly variable across datasets. AUC values ranged from 0.43 for XGBoost [44] to 1.00 for RF in Das et al. [32]. Sensitivity ranged from 0.00 for RF [69] to 1.00 for RF [32]. Specificity ranged from 0.40 for RF [50] to 1.00 in RF models [32,35,69]. PPV ranged from 0.40 [70] to 1.00 [32,35]. NPV ranged from 0.72 in an RF scenario evaluated by Daniels et al. [60] to 1.00 [32]. Accuracy ranged from 0.50 for XGBoost [44] to 1.00 for RF [32]. F1-scores ranged from 0.70 for CART [46] to 1.00 for RF [32]. Overall, the wide range of reported values highlights substantial model- and dataset-level variability within the tree-ensemble family (Table 2).

#### 3.5.3 Deep learning.

Deep-learning models included ANN/MLP architectures, CNN- and transfer-learning models, recurrent neural networks, GANs, transformer-based approaches, and related neural-network architectures. These models were evaluated both as standalone approaches and within studies comparing multiple AI/ML algorithms. Reported AUC values ranged from 0.76 for ANN models [67] to 1.00 [32]. Sensitivity ranged from 0.00 for the MLP model [69] to 1.00 [32,38,55,65]. Specificity ranged from 0.51 for ANN [39] to 1.00 [32,33,38,55,65,69]. PPV ranged from 0.54 for ANN [60] to 1.00 [32,33,38,55,65]. NPV ranged from 0.55 for ANN [53] to 1.00 [32,38,55,65]. Accuracy ranged from 0.70 in ANN scenarios [60] to 1.00 [32,38,55,65]. F1-scores ranged from 0.73 for the Deep/LBP MLP model [37] to 1.00 [32,38]. Although several deep-learning models achieved very high performance, the wide range of estimates does not support uniformly superior performance for this model family (Table 2).

#### 3.5.4 Heuristic approaches.

Only one study evaluated a heuristic AI approach. The BRBES evidential-reasoning system reported an AUC of 0.92 in a dataset of 250 participants, with no additional diagnostic performance metrics available [52].

### 3.6 Regression metrics results

Regression-type metrics were rarely reported across studies. Despite extracting all available performance indicators, only one study [45] provided a true regression metric, reporting a PLSR blind-prediction RMSE of 0.017, indicating excellent agreement between predicted and observed spectral values. No other included study reported RMSE, MAE, MSE, SMAPE, MAPE, R^2^, r or related regression indicators.

### 3.7 Assessment of risk of bias using PROBAST

Using the Prediction Model Risk of Bias Assessment Tool (PROBAST tool) across four domains (participants, predictors, outcome, analysis), most included studies were judged at high overall risk of bias (Fig 5). Out of 43 studies, 30 (69.8%) [30–34,36–38,40–43,45–50,52,54,55,57,58,60,62–64,67,68,71] had high overall risk, 7 (16.3%) [35,44,51,56,59,66,70] were rated low, and 6 (14%) [29,39,53,61,65,69] were unclear. Risk of bias in the predictors domain was generally low, whereas judgements for the participants and outcome domains were more mixed, with several studies classified as high or unclear. The analysis domain represented the main source of concern, with many studies rated at high risk because of issues such as small sample sizes, limited or absent external validation, and suboptimal handling of model overfitting and performance evaluation. Regarding applicability, concerns were generally limited: 34 studies (79.1%) had low overall applicability concern, 6 (14.0%) were rated as unclear, and 3 (7.0%) had high applicability concern (S1 Appendix, Table 4).

**Fig 5:**
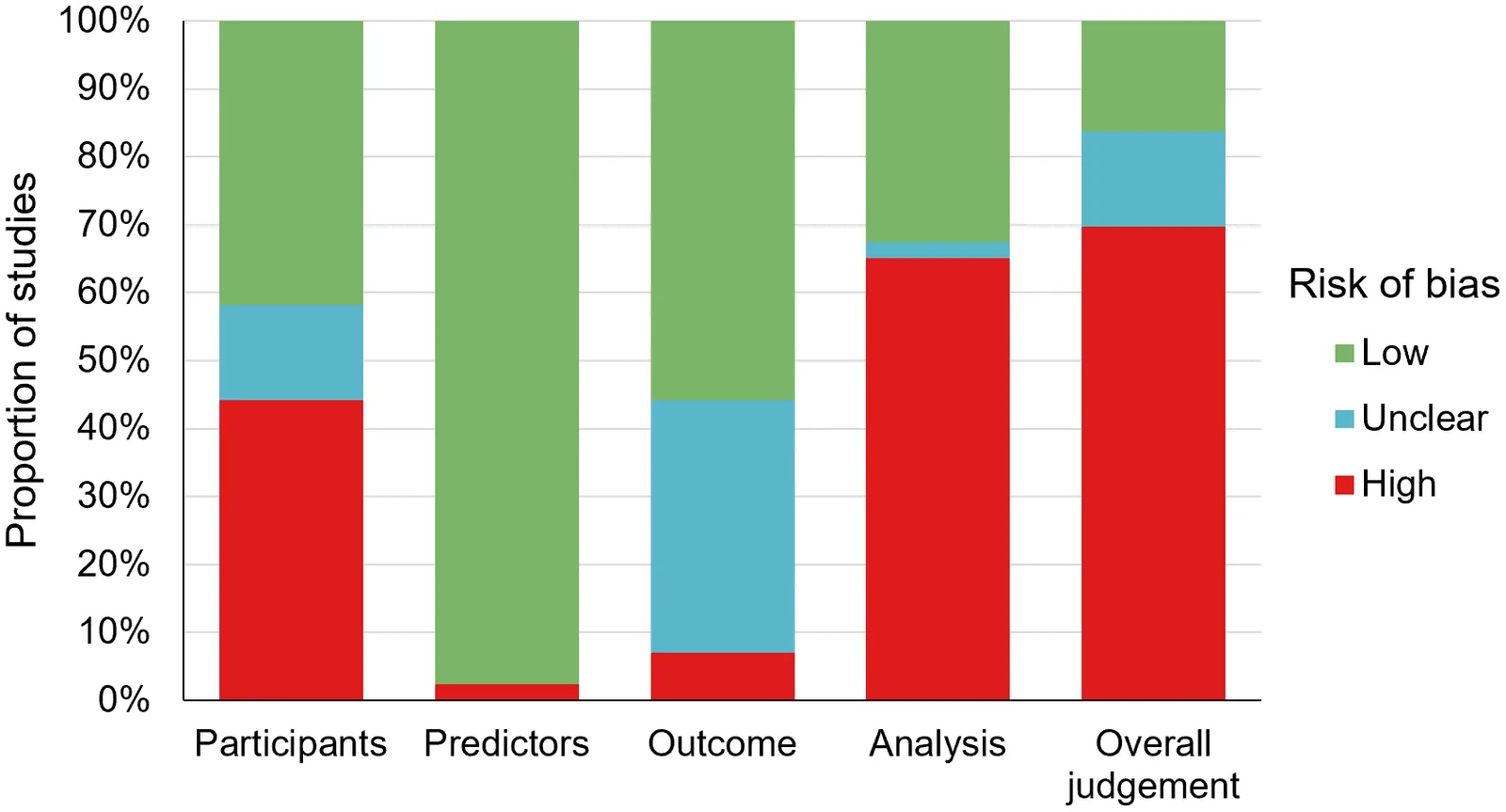
Risk-of-bias assessment across the four PROBAST domains and the overall risk-of-bias judgement. Summary of risk-of-bias judgements for participants, predictors, outcome, and analysis, together with the overall risk-of-bias judgement, across the included diagnostic prediction model studies.

### 3.8 Statistical analysis

#### 3.8.1 Meta-analysis.

Across the revised quantitative synthesis, pooled diagnostic performance estimates were generally high, although substantial heterogeneity was observed across outcomes (Table 3; Fig 6). Because several included studies evaluated more than one AI/ML model, the number of observations contributing to each synthesis refers to model-level estimates rather than to independent studies.

**Table 3:** Summary of meta-analytic estimates, heterogeneity statistics, and small-study effect assessments for overall analyses and prespecified subgroup analyses.

Metric		Overall	Model			Dengue only
			Classical ML	DL	Tree-ensemble	
**Accuracy**	**N**	104	53	25	26	103
	**Random-effects estimate (95% CI)**	0.904 (0.888-0.918)	0.897 (0.878-0.913)	0.912 (0.871-0.941)	0.910 (0.868-0.939)	0.903 (0.887-0.918)
	**I² (%)**	97.2%	92.0%	95.6%	99.1%	97.3%
	**τ²**	0.6730	0.3843	0.9328	1.1301	0.6756
	**Cochran’s Q test p-value**	<0.0001	<0.0001	<0.0001	<0.0001	<0.0001
	**Kendall’s τ**	0.079	0.192	0.447	0.189	0.080
	**Kendall’s τ p-value**	0.2347	0.0443	0.0014	0.1784	0.2357
**PPV**	**N**	81	39	22	20	80
	**Random-effects estimate (95% CI)**	0.903 (0.876-0.925)	0.901 (0.862-0.931)	0.910 (0.861-0.942)	0.894 (0.812-0.943)	0.903 (0.875-0.925)
	**I² (%)**	96.3%	89.8%	85.6%	99.0%	96.4%
	**τ²**	1.3490	1.1555	0.9024	2.1109	1.3610
	**Cochran’s Q test p-value**	<0.0001	<0.0001	<0.0001	<0.0001	<0.0001
	**Kendall’s τ**	0.296	0.379	0.437	0.323	0.308
	**Kendall’s τ p-value**	0.0001	0.0007	0.0039	0.0476	0.0001
**NPV**	**N**	86	42	23	21	85
	**Random-effects estimate (95% CI)**	0.882 (0.859-0.902)	0.869 (0.840-0.894)	0.871 (0.808-0.915)	0.913 (0.866-0.944)	0.881 (0.858-0.902)
	**I² (%)**	95.8%	89.0%	89.5%	98.6%	95.8%
	**τ²**	0.7082	0.4075	0.9041	1.0590	0.7093
	**Cochran’s Q test p-value**	<0.0001	<0.0001	<0.0001	<0.0001	<0.0001
	**Kendall’s τ**	0.144	0.268	0.399	0.072	0.136
	**Kendall’s τ p-value**	0.0514	0.0130	0.0072	0.6503	0.0661
**AUC**	**N**	31	18	5	7	30
	**Random-effects estimate (95% CI)**	0.954 (0.936-0.968)	0.953 (0.930-0.969)	0.943 (0.909-0.965)	0.963 (0.897-0.987)	0.955 (0.937-0.969)
	**I² (%)**	97.7%	88.7%	79.5%	99.7%	97.8%
	**τ²**	0.8832	0.6231	0.2311	2.1308	0.9070
	**Cochran’s Q test p-value**	<0.0001	<0.0001	<0.0001	<0.0001	<0.0001
	**Kendall’s τ**	0.131	0.262	0.000	0.556	0.131
	**Kendall’s τ p-value**	0.3133	0.1370	1.0000	0.1022	0.3235
**Sensitivity**	**N**	86	42	23	21	85
	**Univariate random-effects estimate (95% CI)**	0.873 (0.825-0.909)	0.838 (0.745-0.902)	0.904 (0.833-0.947)	0.892 (0.797-0.946)	0.872 (0.823-0.909)
	**I² (%)**	98.1%	96.4%	91.8%	99.2%	98.2%
	**τ²**	2.7575	3.2295	1.8678	2.6986	2.7996
	**Cochran’s Q test p-value**	<0.0001	<0.0001	<0.0001	<0.0001	<0.0001
	**Kendall’s τ**	0.037	0.183	0.357	−0.206	0.045
	**Kendall’s τ p-value**	0.6143	0.0923	0.0174	0.1937	0.5484
	**Bivariate random-effects estimate (95% CI)**	0.861 (0.809-0.900)	0.817 (0.710-0.891)	0.909 (0.861-0.941)	0.889 (0.803-0.940)	0.860 (0.808-0.900)
	**Deeks’ test statistic**	0.264	−1.380	−1.348	0.767	0.251
	**Deeks’ test p-value**	0.7926	0.1753	0.1919	0.4525	0.8027
**Specificity**	**N**	86	42	23	21	85
	**Univariate random-effects estimate (95% CI)**	0.908 (0.879-0.931)	0.912 (0.876-0.938)	0.896 (0.819-0.942)	0.909 (0.831-0.953)	0.908 (0.878-0.931)
	**I² (%)**	97.2%	90.5%	91.9%	99.2%	97.2%
	**τ²**	1.6920	1.1810	1.8511	2.4798	1.7070
	**Cochran’s Q test p-value**	<0.0001	<0.0001	<0.0001	<0.0001	<0.0001
	**Kendall’s τ**	0.277	0.379	0.462	0.238	0.274
	**Kendall’s τ p-value**	0.0002	0.0005	0.0023	0.1433	0.0002
	**Bivariate random-effects estimate (95% CI)**	0.902 (0.871-0.927)	0.908 (0.865-0.938)	0.871 (0.810-0.914)	0.902 (0.825-0.947)	0.902 (0.870-0.927)
	**Deeks’ test statistic**	0.264	−1.380	−1.348	0.767	0.251
	**Deeks’ test p-value**	0.7926	0.1753	0.1919	0.4525	0.8027

Random-effects pooled estimates are reported for accuracy, AUC, PPV, and NPV, together with heterogeneity statistics. Sensitivity and specificity were jointly synthesised using a bivariate random-effects model. Results are presented for the overall analyses, AI/ML model categories, and dengue-only analyses. Kendall’s τ was used to assess funnel plot asymmetry for univariate outcomes, whereas Deeks’ test was used for the bivariate diagnostic accuracy analysis. For sensitivity and specificity, univariate random-effects estimates and heterogeneity statistics are reported descriptively; bivariate random-effects estimates represent the primary pooled estimates. N denotes the number of model-level estimates contributing to each synthesis, not the number of independent studies.

**Fig 6:**
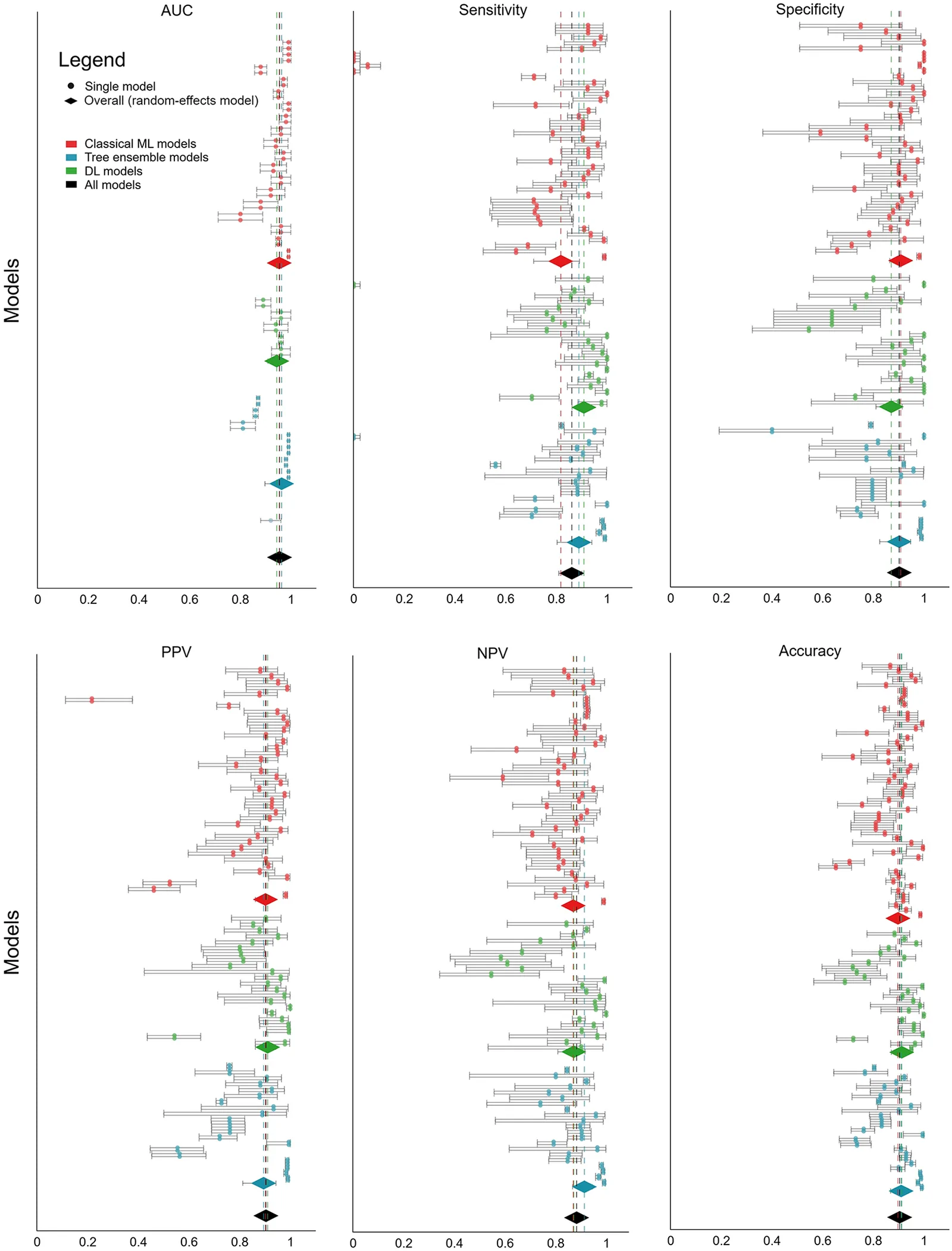
Meta-analytic summary of diagnostic performance of AI models for mosquito-borne viral infections. Forest plots displaying model-level estimates and pooled random-effects estimates of area under the curve (AUC), accuracy, sensitivity, specificity, positive predictive value (PPV), and negative predictive value (NPV). Individual points represent model-level estimates with 95% confidence intervals. Pooled random-effects estimates are shown as diamonds. Sensitivity and specificity pooled estimates were obtained from the bivariate random-effects model.

For discrimination, the overall random-effects pooled area under the curve (AUC) was 0.954 (95% CI 0.936–0.968), based on 31 model-level estimates. Accuracy was available for 104 model-level estimates, yielding a pooled estimate of 0.904 (95% CI 0.888–0.918). Positive predictive value (PPV) and negative predictive value (NPV) were also high, with pooled estimates of 0.903 (95% CI 0.876–0.925; N = 81) and 0.882 (95% CI 0.859–0.902; N = 86), respectively.

Between-model heterogeneity remained substantial for all univariate outcomes. The I^2^ values were 97.7% for AUC, 97.2% for accuracy, 96.3% for PPV, and 95.8% for NPV, with all Cochran’s Q tests indicating statistically significant heterogeneity (p < 0.0001). The corresponding τ^2^ estimates and complete heterogeneity statistics are reported in Table 3. These findings indicate considerable variability in reported model performance across the included evidence base and support a cautious interpretation of the pooled estimates as descriptive summaries.

Sensitivity and specificity were jointly synthesised using a bivariate random-effects model to account for their correlation. Based on 86 paired model-level estimates, pooled sensitivity was 0.861 (95% CI 0.809–0.900) and pooled specificity was 0.902 (95% CI 0.871–0.927). The corresponding summary receiver operating characteristic (sROC) curves are presented in Fig 7. Univariate heterogeneity statistics for sensitivity and specificity were also high (I^2^ = 98.1% and 97.2%, respectively) and are reported descriptively in Table 3.

**Fig 7:**
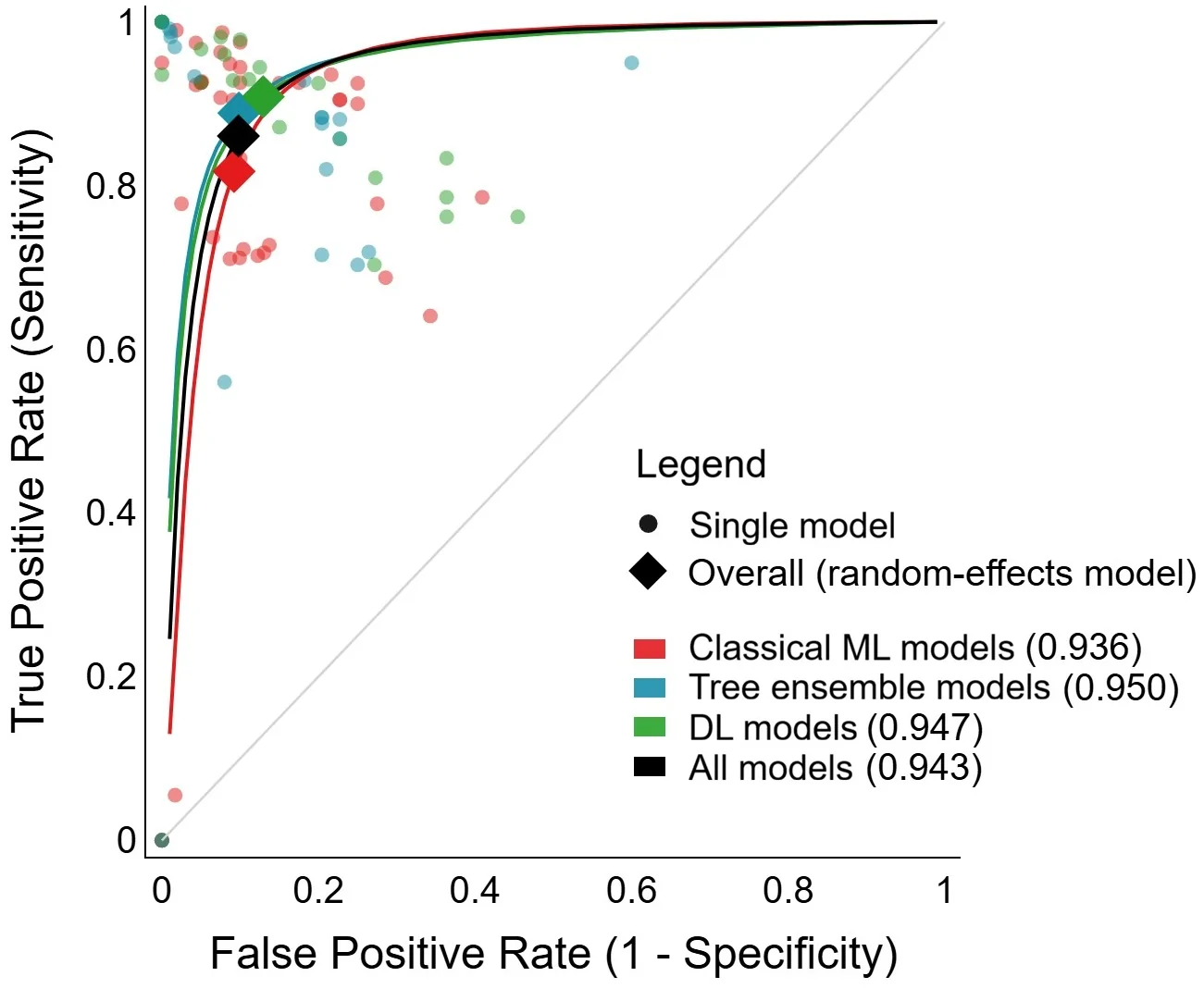
TPR versus FPR scatter plot with summary receiver operating characteristic (sROC) curves. The scatter plot shows the true positive rate (TPR, sensitivity) versus the false positive rate (FPR, 1 − specificity) for individual model-level estimates and pooled results (diamonds). Summary ROC curves were estimated using the bivariate random-effects model, with the corresponding summary area under the curve (sAUC) reported in brackets.

Assessment of small-study effects showed no statistically significant funnel-plot asymmetry for AUC (Kendall’s τ = 0.131, p = 0.3133), accuracy (τ = 0.079, p = 0.2347), or NPV (τ = 0.144, p = 0.0514). In contrast, evidence of asymmetry was observed for PPV (τ = 0.296, p = 0.0001). For the paired sensitivity–specificity analysis, Deeks’ funnel plot asymmetry test did not indicate significant small-study effects (test statistic = 0.264, p = 0.7926). The corresponding funnel plots are provided in S1 Appendix, Figs 13 and 14, while the overall forest plots for AUC, accuracy, sensitivity, specificity, NPV, and PPV are reported in S1 Appendix, Figs 1-6.

#### 3.8.2 Subgroup analysis.

Subgroup analyses by AI/ML model family showed broadly overlapping pooled performance estimates across classical machine learning, deep learning, and tree-ensemble approaches (Table 3; S1 Appendix, Figs 7-12). For accuracy, pooled estimates were 0.897 (95% CI 0.878–0.913) for classical ML, 0.912 (95% CI 0.871–0.941) for deep learning, and 0.910 (95% CI 0.868–0.939) for tree-ensemble models. Corresponding pooled AUC estimates were 0.953 (95% CI 0.930–0.969), 0.943 (95% CI 0.909–0.965), and 0.963 (95% CI 0.897–0.987), respectively.

In the bivariate analysis, pooled sensitivity was 0.817 (95% CI 0.710–0.891) for classical ML, 0.909 (95% CI 0.861–0.941) for deep learning, and 0.889 (95% CI 0.803–0.940) for tree-ensemble models. Pooled specificity was 0.908 (95% CI 0.865–0.938), 0.871 (95% CI 0.810–0.914), and 0.902 (95% CI 0.825–0.947), respectively. Given the overlapping confidence intervals and substantial heterogeneity within model categories, these findings do not provide evidence that any single model family consistently outperformed the others.

Because the evidence base was overwhelmingly focused on dengue, a dengue-only analysis was also performed. Estimates were very similar to the overall synthesis: AUC 0.955 (95% CI 0.937–0.969), accuracy 0.903 (95% CI 0.887–0.918), PPV 0.903 (95% CI 0.875–0.925), NPV 0.881 (95% CI 0.858–0.902), bivariate sensitivity 0.860 (95% CI 0.808–0.900), and bivariate specificity 0.902 (95% CI 0.870–0.927).

Additional exploratory subgroup analyses by geographical location and study design are reported in S1 Appendix, Tables 5 and 6. Because several strata contained relatively few model-level estimates and substantial residual heterogeneity, these analyses were interpreted descriptively and not as evidence of geographical or design-specific superiority.

## 4 Discussion

### 4.1 Summary of the main findings

This systematic review and meta-analysis included 43 studies evaluating AI–based diagnostic models for arboviral infections, predominantly dengue, representing, to our knowledge, the first large-scale quantitative synthesis jointly comparing diagnostic performance across the full spectrum of AI approaches in this field. Unlike previous narrative or scoping reviews [15,72], this work provides a formal meta-analytic evaluation across multiple clinically relevant performance metrics, enabling a robust assessment of both model performance and variability. Most included studies were conducted in Asia, relied on retrospective designs, and used individual-level clinical and laboratory data from hospital-based settings. Furthermore, the available evidence was overwhelmingly dominated by dengue studies, with only limited evidence for Zika and chikungunya and no eligible studies identified for West Nile virus, yellow fever, or Rift Valley fever. Consequently, the pooled estimates presented in this review primarily reflect the performance of AI models for dengue diagnosis and should not be interpreted as directly applicable to other mosquito-borne viral infections. A wide range of AI models was identified, including classical machine-learning algorithms, tree-ensemble methods, deep-learning architectures, and heuristic approaches, with several studies evaluating multiple standalone AI/ML methods. At the individual study level, most models demonstrated moderate to excellent diagnostic performance, with reported AUC values frequently exceeding 0.85. Classical machine-learning and tree-ensemble approaches were the most frequently represented model families, whereas deep-learning approaches were less common. Heuristic approaches were represented by a single study and were therefore not included in quantitative subgroup comparisons. The meta-analysis provided a quantitative descriptive synthesis of these findings, while also demonstrating substantial heterogeneity across the available evidence. Under random-effects models, pooled estimates suggested generally high diagnostic performance, although these summary estimates should be interpreted in light of the substantial clinical and methodological heterogeneity across the included studies. Four methodological model families were used for model-level classification: classical machine learning, tree-ensemble methods, deep learning, and heuristic approaches. Studies evaluating models from more than one family were additionally identified as “Multiple AI/ML approaches” in the descriptive study-level tables. The remaining categories were represented by a limited number of studies and displayed substantial methodological variability, precluding meaningful comparative interpretation. Subgroup analysis stratified by AI model type showed broadly comparable pooled performance across classical ML, tree ensemble, and deep learning approaches, with no single model consistently outperforming others.

### 4.2 Interpretation of the results

The results of this systematic review and meta-analysis provide a detailed and quantitatively grounded overview of the current performance, methodological characteristics, and limitations of AI–based diagnostic models for dengue and other arboviral infections. At the individual study level, the observation that most models achieved moderate to excellent performance with AUC values frequently exceeding conventional thresholds for satisfactory discrimination aligns with prior work emphasizing the promise of ML in this domain. Similar conclusions were drawn by da Silva Neto et al., who identified a predominance of tree-based ML algorithms showing favourable performance in classifying dengue and other arboviruses based on clinical features, albeit within a limited set of studies and without meta-analytic synthesis [14]. The majority of diagnostic models for dengue fever utilized peripheral blood parameters (e.g., platelet count, white blood cell count) as key features, achieving high predictive accuracy (e.g., high sensitivity and specificity), which could significantly impact early clinical management [73]. The meta-analysis extends the existing literature by providing pooled descriptive estimates across multiple clinically relevant performance metrics under random-effects assumptions. This quantitative consolidation extends previous systematic reviews and aggregate syntheses [15,74,75]. It also complements a recent comparative meta-analysis restricted to head-to-head evaluations of AI/ML versus conventional diagnostic approaches, which reported favourable comparative performance for sensitivity, specificity, accuracy, and NPV, but also substantial heterogeneity and limited external validation [76]. The present review extends that evidence by considering the broader body of AI/ML diagnostic studies irrespective of whether a conventional comparator was available.

Despite this overall favourable picture, the pervasive and extreme heterogeneity observed across all pooled outcomes strongly suggests that performance estimates vary substantially according to study-specific contexts. The consistently high heterogeneity observed across the random-effects syntheses underscores that pooled diagnostic performance is strongly influenced by study-specific populations, data sources, modelling strategies, and validation procedures. Accordingly, the pooled estimates presented in this review should be interpreted as descriptive summaries of a highly heterogeneous evidence base rather than precise or universally generalizable measures of expected model performance. Methodological variability (e.g., retrospective versus prospective designs), case-mix differences, prevalence variation, predictor selection, and differing validation strategies likely contribute to this observed heterogeneity. Such variability has also been highlighted in broader infectious disease AI reviews, which note that model performance can differ widely according to data source characteristics and intended use cases [77].

Although pooled performance estimates were generally high, they should be interpreted as descriptive summaries of the currently available literature rather than as definitive estimates of expected real-world diagnostic performance. The included studies differed substantially in terms of populations, predictors, reference standards, modelling strategies, and validation approaches, and most were judged to be at high risk of bias.

Subgroup analysis stratified by AI model type further contextualize these nuances. While deep learning models sometimes achieved high point estimates, their small study numbers and narrower application domains limit confidence in their comparative advantage. Tree ensemble and classical ML models demonstrated broadly similar performance once random-effects considerations were applied, suggesting that algorithmic choice per se may be secondary to data quality, feature representation, and validation rigor. These patterns resonate with findings from other systematic appraisals of AI in tropical disease diagnosis, which similarly observed that ensemble methods often dominate the literature and that neural network–based approaches remain under-explored or applied in limited settings [14,78].

The interpretation of near-perfect performance in some individual studies must be tempered by the recognition of common methodological limitations. High reported metrics in small or homogeneous datasets likely reflect overfitting and lack of external validation rather than true generalizable accuracy, a concern repeatedly emphasized in AI applications in infectious disease contexts [79,80]. This is corroborated by the risk-of-bias assessment, which identified prevalent analytical weaknesses, including limited handling of missing data, absence of calibration assessment, and reliance on internal validation, issues known to inflate performance estimates. Given that most included studies were judged to be at high overall risk of bias, the frequently reported high AUCs and near-perfect accuracies should be interpreted with caution. These performance estimates may partly reflect methodological limitations, including retrospective study designs, small sample sizes, reliance on internal validation, incomplete reporting of missing data handling, and the absence of prospective evaluation, rather than true model generalizability.

Calibration was formally assessed in only 4 of 43 studies (9.3%), despite its central role in determining whether predicted probabilities correspond to observed event rates. High discrimination alone does not ensure reliable probability estimates, particularly when models are transferred to populations with different disease prevalence or case-mix. Future studies should routinely report calibration plots, calibration intercept and slope, and Brier scores, and should consider decision-curve analysis when evaluating clinical utility.

Geographical imbalance is another critical dimension that shapes result interpretation. The concentration of studies in Asia mirrors the epidemiological burden of dengue in tropical and subtropical regions, but simultaneously limits insights into global model applicability. Although multi-country datasets were hypothesized to facilitate more generalizable models, their rarity in this corpus prevents robust evaluation of geographical transportability. Importantly, this limitation extends beyond representativeness alone. AI models developed and validated predominantly in Asian hospital-based dengue cohorts may not perform similarly in underrepresented regions, such as Africa and Latin America, where disease prevalence, co-infections, healthcare infrastructure, laboratory availability, population characteristics, and clinical case-mix may differ substantially [81,82]. Consequently, the pooled performance estimates reported in this review should not be interpreted as evidence of global applicability, as they may conceal clinically important reductions in performance in settings that remain underrepresented in the current literature. This research gap aligns with concerns expressed in broader reviews of AI for infectious disease management, which call for more geographically diverse and externally validated models before clinical integration can be confidently recommended [83,84].

### 4.3 Public health implications and future directions

The findings of this review and meta-analysis have important implications for public health practice, research prioritization, and the strategic integration of AI into arboviral disease surveillance and clinical workflows. The consistently high pooled diagnostic performance of AI models suggests that, if appropriately validated and implemented, these tools could enhance early detection and triage of dengue and other arboviral infections. Early and accurate identification of cases is critical for patient management and for timely public health response [85], particularly in resource-constrained settings where laboratory confirmation may be delayed or unavailable [86]. Prior studies have emphasized the potential of AI for real-time disease detection and outbreak prediction, suggesting that ML models can complement traditional epidemiological surveillance to accelerate response times and improve allocation of limited healthcare resources [87,88].

However, translating the high diagnostic performance reported in the included studies into public health impact requires attention to generalizability, equity, and validity across diverse epidemiological contexts. The geographical imbalance identified in this corpus, raises concerns about the applicability of AI models in under-represented regions, particularly in Africa and parts of the Americas, where arboviral infections are increasingly recognized as major public health burdens. This limitation mirrors broader critiques in the global health AI literature, which highlight the risk that models trained in data-rich environments may not generalize to settings with different population structures, disease prevalence, or healthcare infrastructures [89–92].

Incorporating interpretability and explainability techniques can serve as an additional layer of model validation, providing insights into potential sources of bias, supporting assessment of model reliability across contexts, and promoting transparency. Our findings suggest that, to date, the adoption of such techniques remains suboptimal, a gap that could become increasingly critical given the growing complexity of AI models, including deep learning and large language models. Such approaches are critical for fostering trust among clinicians and public health practitioners, which may ultimately be decisive for the responsible adoption and integration of AI tools into surveillance and decision-making workflows [93].

Moreover, the scarcity of externally validated and prospectively tested models further constrains the ability to translate AI performance into actionable public health tools. Most models remain at the proof-of-concept stage, with limited evidence that their reported discrimination translates into clinically meaningful improvements in patient management or public health decision-making. Importantly, strong discrimination in retrospective datasets should not be equated with clinical utility. Before AI models can support routine clinical triage or diagnostic decision-making, they should demonstrate adequate calibration, clinically meaningful decision thresholds, prospective and external validation, seamless integration into existing clinical workflows, and reliance on predictor variables that are routinely available at the point of care. Furthermore, implementation studies evaluating turnaround time, cost-effectiveness, and the potential consequences of false-positive and false-negative predictions are essential to determine whether these models provide meaningful benefits in real-world healthcare settings. This challenge is not unique to AI applications for arboviral infections but has also been highlighted in other areas of medical AI, where excellent performance on benchmark datasets is considered insufficient to support clinical implementation without rigorous external validation, methodological transparency, and model interpretability [94]. To bridge this gap, future work should prioritize prospective cohort studies, multi-country validation cohorts, and implementation science frameworks that explicitly evaluate clinical utility, workflow integration, cost-effectiveness, and model calibration in operational settings. Such designs are increasingly advocated as essential steps toward responsible AI deployment in health systems [95].

Another critical direction is the development of AI models that can integrate multi-modal data sources, such as clinical metadata, laboratory results, imaging data, entomological surveillance, and environmental variables, to improve robustness and contextual sensitivity. Emerging research suggests that multimodal AI systems may offer superior performance and resilience to shifts in data distribution compared with models relying on a single data type [96–98].

Ethical, legal, and equity considerations must also guide future development and deployment. AI models have the potential to amplify existing disparities if deployed without safeguards to ensure fairness and accessibility. For example, models that perform well in urban referral hospitals may underperform in rural or under-resourced environments, potentially exacerbating inequities in diagnostic access and outcomes. Research into bias detection, fairness-aware algorithms, and participatory evaluation with local stakeholders is essential to ensure that AI enhances rather than undermines global health equity [99–101].

Finally, building regional and international data-sharing consortia will be essential to overcome current limitations in data diversity and volume. Multi-institutional collaborations can facilitate the generation of more heterogeneous datasets, support external validation efforts, and enable benchmarking of AI tools across populations. Initiatives such as the WHO Global Arbovirus Initiative reflect emerging global commitments to strengthen arboviral surveillance and response, and AI has the potential to be a core analytic component of such frameworks, provided that models are rigorously evaluated and contextualized [102,103].

### 4.4 Strengths and limitations

This review has several important strengths. First, it represents the most comprehensive synthesis to date of AI–based diagnostic models for dengue and other arboviral infections, combining a systematic review with a formal meta-analysis across multiple clinically relevant performance metrics. By quantitatively pooling AUC, accuracy, sensitivity, specificity, PPV, and NPV, this study moves beyond narrative descriptions and provides a quantitative descriptive synthesis of the diagnostic performance reported in the available literature. Second, the inclusion of subgroup analyses stratified by AI model type offers additional insight into sources of heterogeneity and allows a more nuanced interpretation of algorithmic performance. Third, the structured assessment of methodological quality using PROBAST strengthens the interpretability of the findings by explicitly addressing the risk of bias across key domains.

Several limitations must also be acknowledged. Most included studies were retrospective, single-country investigations, predominantly conducted in Asia, which limits the geographical representativeness and external validity of the findings. Surprisingly, Latin America, where several major outbreaks occurred in the last decades, was under-represented. European representation was limited to the Belgian component of one multi-country study [70], while Africa and the Americas remained substantially under-represented. As a limitation of the study, most data derive from hospital-based series, limiting the contribution of data obtained from outbreak investigation. In addition, several included studies evaluated multiple AI/ML models using the same or overlapping datasets, meaning that some model-level performance estimates were not statistically independent. The pooled estimates should therefore be interpreted as descriptive summaries of reported model performance rather than as independent study-level effects. Extreme heterogeneity was observed across all pooled analyses, reflecting substantial variability in study design, populations, predictors, reference standards, modelling strategies, and validation approaches. Consequently, the pooled estimates should be interpreted as descriptive summary indicators of the available evidence rather than as precise estimates of real-world diagnostic performance. Furthermore, PPV and NPV are inherently dependent on disease prevalence. Because prevalence and case balance varied across the included datasets, and several studies used case-control sampling or class-balancing procedures, pooled PPV and NPV should be interpreted with particular caution and should not be assumed to transfer directly to populations with different disease prevalence. These differences also limit direct comparability of predictive values across studies. In addition, external validation, calibration assessment, and prospective evaluation were rarely reported, increasing the likelihood of optimistic performance estimates. Evidence of potential publication bias for selected outcomes further suggests that favourable results may be over-represented in the published literature. Heterogeneity and incomplete reporting of performance metrics constrained direct comparability across studies. Moreover, the comparative performance of AI models may be influenced not only by the modelling approach itself but also by differences in dataset characteristics, including size, feature composition, and data format, which limits the attribution of observed differences solely to the model class. Finally, the analysis relied on reported aggregate measures rather than individual participant data. Despite these limitations, the review provides a rigorous and transparent synthesis of the available evidence and highlights critical methodological and contextual gaps that should guide future research in this field. Future studies should also adhere to reporting standards such as TRIPOD+AI [104] to improve transparency, reproducibility, and completeness of AI prediction model reporting.

## 5 Conclusions

This systematic review and meta-analysis synthesises the current evidence on AI-based diagnostic models for arboviral infections and shows that the included studies generally reported high diagnostic performance across multiple clinically relevant metrics.

No single AI paradigm consistently outperformed the others; given the overlapping confidence intervals and substantial heterogeneity, comparative differences between model families remain uncertain. Taken together, this work suggests that AI has the potential to support the early diagnosis of arboviral infections. However, because the available evidence is highly heterogeneous, frequently at high risk of bias, and rarely externally validated, the pooled estimates should be interpreted as descriptive summaries of the published literature rather than definitive estimates of real-world diagnostic performance. Overall robust multi-country validation, prospective evaluation, calibration assessment, workflow integration and equitable data representation are essential before reliable estimates of real-world performance can be established and widespread clinical or public health implementation can be recommended. Future research should extend AI-based diagnostic model development and validation to other clinically important arboviral infections, including West Nile virus, yellow fever, and Rift Valley fever, for which evidence remains absent.

## Supporting information

S1 Checklist PRISMA checklist. Preferred reporting items for systematic reviews and meta-analyses (PRISMA 2020) checklist. (DOCX)

S1 Appendix Supplementary materials. Search strategies, study characteristics, and additional meta-analytic results.
(DOCX)
