# Supplementary material for: Diagnostic performance of artificial intelligence in early diagnosis of mosquito-borne viral infections: Systematic review and meta-analysis
Source: PLOS Digit Health. 2026 Oct 27;5(10):e0001796. doi: 10.1371/journal.pdig.0001796 (PMC13712693; doi:10.1371/journal.pdig.0001796)
Supplement: Supplementary file 2 [file pdig.0001796.s002.docx]

**S1 Appendix. Supplementary materials**

**Table 1.** Full search strings used in PubMed, Embase, and Scopus, including all controlled vocabulary terms and free-text keywords, reported exactly as executed.

| **Database** | **Search strategy** | **Number of identified records** |
| --- | --- | --- |
| PUBMED | ( "dengue"[ti] OR "dengue fever"[ti] OR DENV[tiab] OR zika[ti] OR ZIKV[tiab] OR chikungunya[ti] OR CHIKV[tiab] OR "west nile"[ti] OR WNV[tiab] OR "yellow fever"[ti] OR YFV[tiab] OR "rift valley fever"[ti] OR RVF[tiab] OR RVFV[tiab] OR arbovirus*[ti] OR mosquito*[ti] OR "mosquito-borne"[ti] OR "vector-borne"[ti] OR "mosquito transmitted"[ti] OR "mosquito vector"[ti] OR aedes[ti] OR culex[ti] )  AND ( "artificial intelligence"[tiab] OR "machine learning"[tiab] OR "deep learning"[tiab] OR "neural network*"[tiab] OR "natural language processing"[tiab] OR NLP[tiab] OR AI[tiab] OR "AI-driven"[tiab] OR "support vector machine*"[tiab] OR "random forest*"[tiab] OR "gradient boosting"[tiab] OR XGBoost[tiab] OR LightGBM[tiab] OR CatBoost[tiab] OR "decision tree*"[tiab] OR "bayesian network*"[tiab] OR "naive bayes"[tiab] OR "k-nearest neighbor*"[tiab] OR "nearest neighbor*"[tiab] OR "ensemble learning"[tiab] OR "transfer learning"[tiab] OR "time series"[tiab] OR autoregressive[tiab] OR ARIMA[tiab] OR LSTM[tiab] OR "convolutional neural network*"[tiab] OR CNN[tiab] OR transformer*[tiab] OR "graph neural network*"[tiab] OR GNN[tiab] OR spatiotemporal[tiab] OR "spatio-temporal"[tiab] OR "spatial temporal"[tiab] OR AutoML[tiab] OR "risk prediction model*"[tiab] OR algorithm*[ti] OR "large language model*"[tiab] OR LLM[tiab] OR ChatGPT[tiab] OR "GPT-3"[tiab] OR "GPT-4"[tiab] ) AND ( predict*[tiab] OR "predictive value*"[tiab] OR forecast*[tiab] OR "outbreak detection"[tiab] OR "epidemic prediction"[tiab] OR projection*[tiab] OR "early diagnos*"[tiab] OR "early detection"[tiab] OR "timely diagnos*"[tiab] OR "presymptomatic detection"[tiab] OR diagnos*[tiab] OR "case detection"[tiab] OR classification[ti] OR screening[ti] ) NOT ( review[pt] OR "systematic review"[pt] OR "meta-analysis"[pt] OR editorial[pt] OR letter[pt] OR comment[pt] OR "case reports"[pt] OR guideline[pt] OR "practice guideline"[pt] OR "clinical trial protocol"[pt] ) | 801 |
| SCOPUS | ( ( TITLE("dengue" OR "dengue fever" OR zika OR chikungunya OR "west nile" OR "yellow fever" OR "rift valley fever" OR arbovirus* OR mosquito* OR "mosquito-borne" OR "vector-borne" OR "mosquito transmitted" OR "mosquito vector" OR aedes OR culex OR culicidae OR "insect vectors") ) OR ( TITLE-ABS-KEY(DENV OR ZIKV OR CHIKV OR WNV OR YFV OR RVF OR RVFV) ) )  AND TITLE-ABS-KEY("artificial intelligence" OR "machine learning" OR "deep learning" OR "neural network*" OR "natural language processing" OR AI OR "AI-driven" OR "support vector machine*" OR "random forest*" OR "gradient boosting" OR XGBoost OR LightGBM OR CatBoost OR "decision tree*" OR "bayesian network*" OR "naive bayes" OR "k-nearest neighbor*" OR "nearest neighbor*" OR "ensemble learning" OR "transfer learning" OR "time series" OR autoregressive OR ARIMA OR LSTM OR "convolutional neural network*" OR CNN OR transformer* OR "graph neural network*" OR GNN OR spatiotemporal OR "spatio-temporal" OR "spatial temporal" OR AutoML OR "risk prediction model*" OR "large language model*" OR LLM OR ChatGPT OR "GPT-3" OR "GPT-4") AND ( TITLE-ABS-KEY(predict* OR "predictive value*" OR forecast* OR "outbreak detection" OR "epidemic prediction" OR projection* OR "early diagnos*" OR "early detection" OR "timely diagnos*" OR "presymptomatic detection" OR diagnos* OR "case detection") OR TITLE(classification OR screening) ) | 1,768 |
| EMBASE | ( 'dengue'/exp OR 'dengue virus'/exp OR "dengue":ti OR "dengue fever":ti OR DENV:ti,ab OR 'zika virus infection'/exp OR 'zika virus'/exp OR zika:ti OR ZIKV:ti,ab OR 'chikungunya virus'/exp OR 'chikungunya fever'/exp OR chikungunya:ti OR CHIKV:ti,ab OR 'west nile fever'/exp OR 'west nile virus'/exp OR "west nile":ti OR WNV:ti,ab OR 'yellow fever'/exp OR 'yellow fever virus'/exp OR "yellow fever":ti OR YFV:ti,ab OR 'rift valley fever'/exp OR 'rift valley fever virus'/exp OR "rift valley fever":ti OR RVF:ti,ab OR RVFV:ti,ab OR 'arbovirus'/exp OR 'arbovirus infection'/exp OR arbovirus*:ti OR 'culicidae'/exp OR 'insect vector'/exp OR mosquito*:ti OR 'mosquito-borne':ti OR 'vector-borne':ti OR 'mosquito transmitted':ti OR 'mosquito vector':ti OR 'aedes'/exp OR 'culex'/exp OR aedes:ti OR culex:ti ) AND ( 'artificial intelligence'/exp OR 'machine learning'/exp OR 'deep learning'/exp OR 'neural network'/exp OR 'natural language processing'/exp OR 'artificial intelligence':ti,ab OR 'machine learning':ti,ab OR 'deep learning':ti,ab OR 'natural language processing':ti,ab OR NLP:ti,ab OR AI:ti,ab OR 'AI-driven':ti,ab OR 'neural network*':ti,ab OR 'support vector machine*':ti,ab OR 'random forest*':ti,ab OR 'gradient boosting':ti,ab OR XGBoost:ti,ab OR LightGBM:ti,ab OR CatBoost:ti,ab OR 'decision tree*':ti,ab OR 'bayesian network*':ti,ab OR 'naive bayes':ti,ab OR 'k-nearest neighbor*':ti,ab OR 'nearest neighbor*':ti,ab OR 'ensemble learning':ti,ab OR 'transfer learning':ti,ab OR 'time series':ti,ab OR autoregressive:ti,ab OR ARIMA:ti,ab OR LSTM:ti,ab OR 'convolutional neural network*':ti,ab OR CNN:ti,ab OR transformer*:ti,ab OR 'graph neural network*':ti,ab OR GNN:ti,ab OR spatiotemporal:ti,ab OR 'spatio-temporal':ti,ab OR 'spatial temporal':ti,ab OR AutoML:ti,ab OR 'risk prediction model*':ti,ab OR algorithm*:ti OR 'large language model*':ti,ab OR LLM:ti,ab OR ChatGPT:ti,ab OR 'GPT-3':ti,ab OR 'GPT-4':ti,ab ) AND ( predict*:ti,ab OR 'predictive value*':ti,ab OR forecast*:ti,ab OR 'outbreak detection':ti,ab OR 'epidemic prediction':ti,ab OR projection*:ti,ab OR 'early diagnos*':ti,ab OR 'early detection':ti,ab OR 'timely diagnos*':ti,ab OR 'presymptomatic detection':ti,ab OR diagnos*:ti,ab OR 'case detection':ti,ab OR classification:ti OR screening:ti ) NOT ( 'systematic review'/exp OR 'meta analysis'/exp OR editorial:it OR letter:it OR comment:it OR 'case report'/exp OR guideline/exp OR 'practice guideline'/exp OR 'clinical trial protocol'/exp ) | 1,962 |

**Table 2.** Detailed characteristics of the included studies.

| **First author (Year)** | **Study period** | **Temporal stage of data** | **Missing/imbalanced data handling** | **Calibration** | **Data split** | **Implementation readiness** |
| --- | --- | --- | --- | --- | --- | --- |
| Abdualgalil, B. (2022) | 2017-2019 | Data available at time of diagnosis | Imputed + SMOTE + ENN | NA | 70/30 train/test + 10-fold CV | Research only |
| Balamurugan, S.A.A. (2020) | Jan 2018-Jan 2019 | Follow-up | SMOTE | NA | 10-fold CV (Weka) | Research only |
| Balasaravanan, K. (2018) | NA | Data available at time of diagnosis | Imputed missing values (elimination/refilling) | NA | 10-fold CV (Weka) | Research only |
| Bohm, B.C. (2024) | 2016-2019 | Data available at time of diagnosis | NA | NA | 70/30 train/test + 10-fold CV | Research only |
| Bria, Y.P. (2024) | 2017-2023 | Data available at time of diagnosis | SMOTE (SVM-SMOTE) | NA | 10-fold CV | Research only |
| Bria, Y.P. (2025) | 2017-2023 | Data available at time of diagnosis | NA | NA | k-fold CV | Research only |
| Chatterjee, S. (2018) | NA | Data available at time of diagnosis | NA | NA | 10-fold CV | Research only |
| Daniels, B.C. (2024) | NA | Data available at time of diagnosis | NA | NA | 90/10 train/test; also 80/20 and 70/30 explored | Research only |
| Das, S. (2025) | NA | Data available at time of diagnosis | NA | NA | 70/30 train/test + 10-fold CV | Research only |
| Dasgupta, S. (2025) | 2022-2023 | Data available at time of diagnosis | NA | NA | 80/20 train/test | Research only |
| Deepika, R. (2025) | NA | Follow-up | NA | NA | 10-fold CV (Weka) | Research only |
| Falconi-Agapito, F. (2022) | 2018-2019 | Data available at time of diagnosis | RF resampling (50,000 stratified splits) | NA | Stratified 2/3–1/3 train/test ×50,000 RF repeats | Research only |
| Goh, B. (2025) | NA | Data available at time of diagnosis | NA | NA | 70/30 train/test | Research only |
| Hasanah, I. (2020) | Feb-Jun 2017 | Data available at time of diagnosis | NA | NA | 100/20 train/test | Research only |
| Hassan, M. (2022) | 2019 | Data available at time of diagnosis | Balanced dataset (60:40); no advanced handling | NA | 70/15/15 train/val/test | Research only |
| Ho, T.S. (2020) | May-Dec 2015 | Data available at time of diagnosis | Imputed missing labs (median) | Hosmer–Lemeshow test for LR; ML models evaluated with ROC | 70/30 train/test | Research only |
| Hossain, M.S. (2019) | 2017-2018 | Data available at time of diagnosis | Belief degree update (uncertainty handling) | NA | Internal train/test (n=250) | Pilot / Proof-of-concept |
| Husin, N.A. (2020) | 2015 | Data available at time of diagnosis | NA | NA | 70/15/15 train/val/test | Research only |
| Ibrahim, F. (2010) | Aug 2005-Aug 2006 | Data available at time of diagnosis | Excluded | NA | 70/30 train/test | Research only |
| Jáuregui-Velarde, R. (2024) | NA | Data available at time of diagnosis | NA | NA | NA | Pilot / Proof-of-concept |
| Kaur, S. (2022) | 2018-2020 | Data available at time of diagnosis | Excluded | NA | 8-fold CV | Research only |
| Khan, S. (2016) | 2015 | Data available at time of diagnosis | NA | NA | 10-fold CV | Research only |
| Khan, S. (2017) | NA | Data available at time of diagnosis | NA | NA | 5-fold CV | Research only |
| Lu, B. (2025) | 1994-2019 | Data available at time of diagnosis | Excluded | Calibration plots | Cross-dataset evaluation | Research only |
| Madewell, Z.J. (2024) | 2012–2017 | Data available at time of diagnosis | NA | NA | NA | Research only |
| Mayrose, H. (2023)a | NA | Data available at time of diagnosis | NA | NA | 10-fold CV | Research only |
| Mayrose, H. (2023)b | NA | Data available at time of diagnosis | NA | NA | 10-fold CV | Research only |
| Melo, C.F.O.R. (2018) | 2016–2017 | Data available at time of diagnosis | NA | NA | 80/20 train/test + 10-round internal val | Research only |
| Ming, D.K. (2022) | 2010-2014 | Data available at time of diagnosis | <1% missing; XGB native handling | Brier score 0.13 | 80/20 train/test | Research only |
| Obot, O. (2023) | 2017–2021 | Data available at time of diagnosis | SMOTE for class imbalance | NA | 80/20 train/test | Research only |
| Puthenkaleekkal Thankappan, R. (2025) | NA | Data available at time of diagnosis | NA | NA | 66.7/33.3 train/test + 5-fold CV | Research only |
| Qaiser, A. (2024) | Aug-Oct 2023 | Data available at time of diagnosis | Excluded | NA | 70/30 train/test + cross-validation; additional validation on a synthetic external dataset (n = 200) | Research only |
| Ramakrishna, M.M. (2023) | Jan-Aug 2022 | Data available at time of diagnosis | NA | NA | 70/30 train/test | Research only |
| Rao, V.S. (2012) | NA | Data available at time of diagnosis | Imputed missing values | NA | 10-fold CV | Research only |
| Riya, N.J. (2024) | May-Oct 2023 | Data available at time of diagnosis | Imputed missing values + SMOTE | NA | NA | Research only |
| Ruiz Valdez, C.A. (2024) | Training 2006–2015; Testing/validation Jan–Dec 2022 | Data available at time of diagnosis | Balanced test set (1:1) via random sampling; missing excluded | NA | National surveillance 2006–2015 train + 2022 external test | Pilot / Proof-of-concept |
| Saleem, M. (2022) | 2019 | Data available at time of diagnosis | NA | NA | 51/49 train/test | Research only |
| Sa-ngamuang, C. (2018) | Jan 2013-Dec 2015 | Data available at time of diagnosis | NA | NA | 5-fold CV | Research only |
| Suhaeri, S. (2018) | 2016–2017 | Data available at time of diagnosis | Imputed + outlier removal + normalization + feature selection (GCV, RSS) | NA | k-fold CV | Research only |
| Tanner, L. (2008) | NA | Data available at time of diagnosis | Excluded | NA | 10-fold CV | Research only |
| Vu, D.M. (2023) | 2014-2019 | Data available at time of diagnosis | Imputed or excluded when outcome missing | NA | 70/30 train/test + 10-fold CV | Research only |
| Waiwijit, U. (2025) | NA | Data available at time of diagnosis | NA | NA | LOOCV (leave-one-out CV) | Research only |
| Williams, R.J. (2024) | Jan 2007-Dec 2021 | Data available at time of diagnosis | RF internal imputation (proximity) | Brier score 0.15 | 10-fold CV ×100 repeats | Research only |

CHIKV=Chikungunya Virus; CV=Cross-Validation; DENV=Dengue Virus; ENN=Edited Nearest Neighbors; ELISA=Enzyme-Linked Immunosorbent Assay; GCV=Generalized Cross Validation; LOOCV=Leave-One-Out Cross-Validation; NA=Not available; PRNT=Plaque Reduction Neutralization Test; RF=Random Forest; RSS=Residual Sum of Squares; SMOTE=Synthetic Minority Over-sampling Technique; SVM=Support Vector Machine; Weka=Waikato Environment for Knowledge Analysis; XGB=eXtreme Gradient Boosting

**Table 3.** Characteristics of data sources, number of variables, additional performance metrics, and model interpretability/explainability reporting across included AI/ML diagnostic studies.

| **First author (Year)** | **Data Source** | **Number of variables included/considered** | **Other Metrics** | **Model interpretability/ explainability reported** |
| --- | --- | --- | --- | --- |
| Abdualgalil, B. (2022) | Hospital data | 19/21 | NA | Yes |
| Balamurugan, S.A.A. (2020) | Hospital data | 4/20 | NA | Yes |
| Balasaravanan, K. (2018) | Medical agency data | 10/10 | NA | Yes |
| Bohm, B.C. (2024) | Surveillance data (SINAN) | 10/23 | NA | Yes |
| Bria, Y.P. (2024) | Hospital data | 10/13 | NA | No |
| Bria, Y.P. (2025) | Hospital data | 13-19/38 | NA | Yes |
| Chatterjee, S. (2018) | Public microarray dataset | NA | NA | No |
| Daniels, B.C. (2024) | Multicountry serological cohorts (PRNT titres) | Scenario A: 16; Scenario B: 19; Scenario C: 5; Scenario D: 16 | NA | No |
| Das, S. (2025) | Raman spectra (human serum) | NA | MCC: SVM 0.98; NN 1.00; RF 1.00 | No |
| Dasgupta, S. (2025) | Hospital data | 5/5 | NA | Yes |
| Deepika, R. (2025) | Hospital data | 8/NA | NA | No |
| Falconi-Agapito, F. (2022) | Serum samples (n=323; Luminex multiplex immunoassay, IgM/IgG to dengue peptides) | RFM3: 6/9; RFG3: 6/10 | NA | No |
| Goh, B. (2025) | Hospital dataset | 26/43 | NA | No |
| Hasanah, I. (2020) | Hospital data | 10/10 | NA | Yes |
| Hassan, M. (2022) | Hospital data | NA | MCC 0.92; κ 0.92 | No |
| Ho, T.S. (2020) | Electronic medical records (ED visits, 3 hospitals) | 6/6 | NA | No |
| Hossain, M.S. (2019) | Hospital data | 5/5 | NA | Yes |
| Husin, N.A. (2020) | Hospital data | 15/15 | NA | No |
| Ibrahim, F. (2010) | Daily BIA readings (dengue patients, admission to defervescence) | NA | NA | No |
| Jáuregui-Velarde, R. (2024) | NA | NA | NA | No |
| Kaur, S. (2022) | Hospital data | 19/~50 | NA | No |
| Khan, S. (2016) | Raman spectra | NA | NA | No |
| Khan, S. (2017) | Raman spectra (human serum) | NA | NA | Yes |
| Lu, B. (2025) | Publicly available datasets | 3/NA | NA | No |
| Madewell, Z.J. (2024) | Surveillance system dataset | 8, 32, 48 | MCC: LR 0.71; SVM 0.71; RF 0.73; ANN 0.71; AdaBoost 0.75; LightGBM 0.75; XGB 0.76 | No |
| Mayrose, H. (2023)a | Hospital data (PBS images) | 100/1177 | NA | No |
| Mayrose, H. (2023)b | Microscope images | GLSDM: 10; Deep + LBP: 100/1177 | NA | No |
| Melo, C.F.O.R. (2018) | HRMS metabolomic spectra (203 serum samples) | 42/~1000 | NA | Yes |
| Ming, D.K. (2022) | Prospective multicenter AFI cohort | 7/7 | Rolling-window dynamic threshold: NPV 0.90 | No |
| Obot, O. (2023) | Hospital dataset | 18/40 | NA | No |
| Puthenkaleekkal Thankappan, R. (2025) | Raman spectra (human serum) | NA | NA | No |
| Qaiser, A. (2024) | Hospital data | 8/8 | NA | No |
| Ramakrishna, M.M. (2023) | Hospital data | 15/15 | NA | No |
| Rao, V.S. (2012) | Hospital data | 2-6/9-16 | NA | No |
| Riya, N.J. (2024) | Hospital data (CBC lab reports) | 14/14 | NA | Yes |
| Ruiz Valdez, C.A. (2024) | National surveillance data (Mexico, 2006–2015) + 2022 hospital ED cohort | 46/46 | NA | No |
| Saleem, M. (2022) | Raman spectra (PeakSeeker Pro-785 system) | 4-7/1290 | NA | No |
| Sa-ngamuang, C. (2018) | Prospective ED/ward cohort | 16/21 | NA | Yes |
| Suhaeri, S. (2018) | Hospital data | 7/54 | NA | Yes |
| Tanner, L. (2008) | Hospital data | 6/NA | NA | Yes |
| Vu, D.M. (2023) | Hospital data | NA/113 | κ: Boosted LR 0.06 | No |
| Waiwijit, U. (2025) | Raman spectra (human serum) | NA | NA | No |
| Williams, R.J. (2024) | Prospective febrile-illness surveillance cohort | 41/52 | Cohen’s κ: 0.60 | Yes |

ANN = Artificial Neural Network; BIA = Bioelectrical Impedance Analysis; CBC = Complete Blood Count; GLSDM = Gray-Level Spatial Dependence Matrix; HRMS = High-Resolution Mass Spectrometry; IgG = Immunoglobulin G; IgM = Immunoglobulin M; LBP = Local Binary Pattern; LR = Logistic Regression; MCC = Matthews Correlation Coefficient; NA = Not available/not reported; NN = Neural Network; PBS = Peripheral Blood Smear; PRNT = Plaque Reduction Neutralization Test; RF = Random Forest; SINAN = Sistema Nacional de Agravos de Notificação; SVM = Support Vector Machine; XGB = eXtreme Gradient Boosting; κ = Cohen’s kappa.

**Table 4.** Risk of bias and applicability assessment of the included studies according to PROBAST.
Risk of bias was assessed across the four PROBAST domains (participants, predictors, outcome, and analysis), together with the overall risk-of-bias judgement. Applicability concerns were evaluated for the participants, predictors, and outcome domains, with an overall applicability judgement derived accordingly. Judgements were classified as low, high, or unclear.

| **First author (Year)** | **Participants** | **Predictors** | **Outcome** | **Analysis** | **Overall** | **Participants Applicability** | **Predictors Applicability** | **Outcome Applicability** | **Overall Applicability** |
| --- | --- | --- | --- | --- | --- | --- | --- | --- | --- |
| Abdualgalil, B. (2022) | Unclear | High | High | Low | High | Low | Low | Low | Low |
| Balamurugan, S.A.A. (2020) | Low | Low | Unclear | Low | Unclear | Low | High | Low | High |
| Balasaravanan, K. (2018) | High | Low | Low | High | High | Low | Low | Low | Low |
| Bohm, B.C. (2024) | High | Low | Unclear | Low | High | Low | Low | Low | Low |
| Bria, Y.P. (2024) | High | Low | Unclear | High | High | Low | Low | Unclear | Unclear |
| Bria, Y.P. (2025) | High | Low | Unclear | High | High | Low | Low | Unclear | Unclear |
| Chatterjee, S. (2018) | High | Low | Low | High | High | Low | Low | Low | Low |
| Daniels, B.C. (2024) | Low | Low | Low | High | High | Low | Low | High | High |
| Das, S. (2025) | High | Low | Unclear | High | High | Low | Low | Low | Low |
| Dasgupta, S. (2025) | Low | Low | Unclear | High | High | Low | Low | Low | Low |
| Deepika, R. (2025) | High | Low | Low | High | High | Low | High | Low | High |
| Falconi-Agapito, F. (2022) | Low | Low | Low | Low | Low | Low | Low | Low | Low |
| Goh, B. (2025) | Low | Low | Low | High | High | Low | Low | Low | Low |
| Hasanah, I. (2020) | High | Low | Unclear | High | High | Low | Low | Low | Low |
| Hassan, M. (2022) | High | Low | Unclear | High | High | Low | Low | Low | Low |
| Ho, T.S. (2020) | Low | Low | Low | Low | Low | Low | Low | Low | Low |
| Hossain, M.S. (2019) | Low | Low | Low | High | High | Low | Low | Low | Low |
| Husin, N.A. (2020) | High | Low | Unclear | High | High | Low | Low | Low | Low |
| Ibrahim, F. (2010) | High | Low | Low | High | High | Low | Low | Low | Low |
| Jáuregui-Velarde, R. (2024) | Unclear | Low | Low | Unclear | Unclear | Low | Low | Unclear | Unclear |
| Kaur, S. (2022) | Low | Low | Low | Low | Low | Low | Low | Low | Low |
| Khan, S. (2016) | High | Low | High | High | High | Low | Low | Low | Low |
| Khan, S. (2017) | High | Low | Low | High | High | Low | Low | Low | Low |
| Lu, B. (2025) | Unclear | Low | Low | Low | Unclear | Low | Low | Low | Low |
| Madewell, Z.J. (2024) | Low | Low | Low | High | High | Low | Low | Low | Low |
| Mayrose, H. (2023)a | Unclear | Low | Unclear | High | High | Low | Low | Unclear | Unclear |
| Mayrose, H. (2023)b | High | Low | High | High | High | Low | Low | Low | Low |
| Melo, C.F.O.R. (2018) | Low | Low | Low | High | High | Low | Low | Low | Low |
| Ming, D.K. (2022) | Low | Low | Low | Low | Low | Low | Low | Low | Low |
| Obot, O. (2023) | Low | Low | Low | High | High | Low | Low | Low | Low |
| Puthenkaleekkal Thankappan, R. (2025) | High | Low | Unclear | High | High | Low | Low | Low | Low |
| Qaiser, A. (2024) | Low | Low | Low | Low | Low | Low | Low | Low | Low |
| Ramakrishna, M.M. (2023) | Unclear | Low | Unclear | Low | Unclear | Low | Low | Low | Low |
| Rao, V.S. (2012) | High | Low | Unclear | High | High | Low | Low | Low | Low |
| Riya, N.J. (2024) | Unclear | Low | Low | Low | Unclear | Low | Low | Unclear | Unclear |
| Ruiz Valdez, C.A. (2024) | Low | Low | Low | Low | Low | Low | Low | Low | Low |
| Saleem, M. (2022) | High | Low | Low | High | High | Low | Low | Low | Low |
| Sa-ngamuang, C. (2018) | Low | Low | Low | High | High | Low | Low | Low | Low |
| Suhaeri, S. (2018) | High | Low | Low | High | High | Low | Low | Low | Low |
| Tanner, L. (2008) | Low | Low | Unclear | High | High | Low | Unclear | Low | Unclear |
| Vu, D.M. (2023) | Low | Low | Unclear | Low | Unclear | Low | Low | Low | Low |
| Waiwijit, U. (2025) | High | Low | Unclear | High | High | Low | Low | Low | Low |
| Williams, R.J. (2024) | Low | Low | Low | Low | Low | Low | Low | Low | Low |

**Tab 5.** Meta-analytic estimates, heterogeneity statistics, and small-study effect assessments stratified by geographical location.

| **Metric** | | **Continent** | | **Country** | | | | | | |
| --- | --- | --- | --- | --- | --- | --- | --- | --- | --- | --- |
|  |  | **Africa** | **Asia** | **Bangladesh** | **India** | **Indonesia** | **Kenya** | **Pakistan** | **Thailand** | **Yemen** |
| **Accuracy** | **N** | 6 | 91 | 15 | 22 | 16 | 6 | 12 | 10 | 6 |
|  | **Univariate random-effects estimate (95%)** | 0.920 (0.915-0.925) | 0.906 (0.888-0.921) | 0.817 (0.775-0.852) | 0.928 (0.905-0.946) | 0.915 (0.903-0.926) | 0.920 (0.915-0.925) | 0.880 (0.828-0.918) | 0.884 (0.840-0.917) | 0.986 (0.981-0.990) |
|  | **I² (%)** | 0.0% | 97.2% | 57.4% | 74.7% | 65.9% | 0.0% | 71.1% | 84.3% | 72.4% |
|  | **τ²** | 0.0000 | 0.7548 | 0.1461 | 0.3119 | 0.0568 | 0.0000 | 0.3497 | 0.2410 | 0.1194 |
|  | **Cochran's Q test p-value** | 0.7484 | <0.0001 | 0.0035 | <0.0001 | 0.0002 | 0.7484 | 0.0011 | <0.0001 | 0.0005 |
|  | **Kendall's τ** | 1.000 | 0.114 | 1.000 | 0.740 | 0.757 | 1.000 | 0.556 | 0.378 | 0.867 |
|  | **Kendall's τ p-value** | 0.0253 | 0.1124 | <0.0001 | <0.0001 | 0.0001 | 0.0253 | 0.0149 | 0.1557 | 0.0167 |
| **AUC** | **N** | 0 | 29 | 0 | 12 | 0 | 0 | 0 | 5 | 5 |
|  | **Univariate random-effects estimate (95%)** | | 0.959 (0.942-0.971) | | 0.938 (0.909-0.958) | |  |  | 0.958 (0.901-0.983) | 0.988 (0.984-0.991) |
|  | **I² (%)** |  | 97.5% |  | 62.3% |  |  |  | 93.5% | 63.9% |
|  | **τ²** |  | 0.8140 |  | 0.3125 |  |  |  | 0.8051 | 0.0799 |
|  | **Cochran's Q test p-value** | | <0.0001 |  | 0.0007 |  |  |  | <0.0001 | 0.0148 |
|  | **Kendall's τ** | | 0.046 |  | 1.000 |  |  |  | 0.111 | 1.000 |
|  | **Kendall's τ p-value** | | 0.7339 |  | <0.0001 |  |  |  | 0.7947 | 0.0455 |
| **NPV** | **N** | 0 | 73 | 15 | 20 | 0 | 6 | 12 | 10 | 6 |
|  | **Random-effects estimate (95% CI)** | 0.923 (0.917-0.927) | 0.876 (0.847-0.901) | 0.719 (0.660-0.772) | 0.886 (0.848-0.916) | | 0.923 (0.917-0.927) | 0.830 (0.778-0.871) | 0.844 (0.836-0.853) | 0.986 (0.977-0.992) |
|  | **I² (%)** | 0.0% | 95.2% | 21.6% | 39.0% |  | 0.0% | 28.1% | 0.0% | 75.2% |
|  | **τ²** | 0.0000 | 0.8394 | 0.0659 | 0.1994 |  | 0.0000 | 0.0870 | 0.0000 | 0.2623 |
|  | **Cochran's Q test p-value** | 0.9994 | <0.0001 | 0.2067 | 0.0413 |  | 0.9994 | 0.0882 | 0.8453 | 0.0004 |
|  | **Kendall's τ** | 1.000 | 0.248 | 0.806 | 0.473 |  | 1.000 | 0.619 | 0.156 | 0.867 |
|  | **Kendall's τ p-value** | 0.0253 | 0.0020 | <0.0001 | 0.0041 |  | 0.0253 | 0.0065 | 0.6007 | 0.0167 |
| **PPV** | **N** | 0 | 73 | 15 | 20 | 0 | 0 | 12 | 10 | 6 |
|  | **Random-effects estimate (95% CI)** | | 0.913 (0.887-0.933) | 0.853 (0.821-0.881) | 0.936 (0.910-0.956) | |  | 0.901 (0.849-0.937) | 0.907 (0.845-0.946) | 0.987 (0.983-0.990) |
|  | **I² (%)** |  | 95.9% | 4.2% | 43.6% |  |  | 40.7% | 86.3% | 14.6% |
|  | **τ²** |  | 1.2371 | 0.0093 | 0.2821 |  |  | 0.2801 | 0.6323 | 0.0167 |
|  | **Cochran's Q test p-value** | | <0.0001 | 0.3259 | 0.0147 |  |  | 0.0684 | <0.0001 | 0.1631 |
|  | **Kendall's τ** | | 0.288 | 0.767 | 0.699 |  |  | 0.662 | 0.333 | 0.867 |
|  | **Kendall's τ p-value** | | 0.0003 | 0.0001 | <0.0001 |  |  | 0.0031 | 0.2164 | 0.0167 |
| **Sensitivity** | **N** | 6 | 73 | 15 | 20 | 0 | 6 | 12 | 10 | 6 |
|  | **Univariate random-effects estimate (95%)** | 0.008 (0.002-0.031) | 0.904 (0.878-0.924) | 0.846 (0.812-0.875) | 0.926 (0.895-0.948) | | 0.008 (0.002-0.031) | 0.847 (0.754-0.910) | 0.908 (0.870-0.936) | 0.986 (0.977-0.992) |
|  | **I² (%)** | 57.6% | 95.6% | 12.5% | 55.4% |  | 57.6% | 75.8% | 71.1% | 75.7% |
|  | **τ²** | 1.6014 | 1.0519 | 0.0283 | 0.3732 |  | 1.6014 | 0.7397 | 0.1920 | 0.2691 |
|  | **Cochran's Q test p-value** | 0.0102 | <0.0001 | 0.3032 | 0.0006 |  | 0.0102 | 0.0005 | <0.0001 | 0.0003 |
|  | **Kendall's τ** | -1.000 | 0.239 | 1.000 | 0.659 |  | -1.000 | 0.576 | 0.317 | 0.867 |
|  | **Kendall's τ p-value** | 0.0253 | 0.0031 | <0.0001 | 0.0001 |  | 0.0253 | 0.0088 | 0.2275 | 0.0167 |
|  | **Bivariate random-effects estimate (95% CI)** | 0.006 (0.002-0.022) | 0.895 (0.869-0.917) | 0.854 (0.815-0.887) | 0.920 (0.889-0.942) | | 0.006 (0.002-0.022) | 0.838 (0.747-0.901) | 0.899 (0.862-0.927) | 0.986 (0.977-0.992) |
|  | **Deeks' test statistic** | NA | 0.617 | NA | 2.658 |  | NA | 0.628 | 2.725 | NA |
|  | **Deeks' test p-value** | NA | 0.5395 | NA | 0.0160 |  | NA | 0.5441 | 0.0260 | NA |
| **Specificity** | **N** | 6 | 73 | 15 | 20 | 0 | 6 | 12 | 10 | 6 |
|  | **Univariate random-effects estimate (95%)** | 0.999 (0.995-1.000) | 0.887 (0.857-0.912) | 0.731 (0.671-0.783) | 0.905 (0.867-0.932) | | 0.999 (0.995-1.000) | 0.905 (0.871-0.931) | 0.831 (0.723-0.903) | 0.987 (0.983-0.990) |
|  | **I² (%)** | 75.9% | 95.7% | 18.6% | 40.6% |  | 75.9% | 0.0% | 86.4% | 15.6% |
|  | **τ²** | 3.3153 | 1.0907 | 0.0582 | 0.2616 |  | 3.3153 | 0.0000 | 0.7536 | 0.0181 |
|  | **Cochran's Q test p-value** | <0.0001 | <0.0001 | 0.1846 | 0.0342 |  | <0.0001 | 0.8822 | <0.0001 | 0.1507 |
|  | **Kendall's τ** | 1.000 | 0.216 | 1.000 | 0.644 |  | 1.000 | 0.569 | 0.318 | 1.000 |
|  | **Kendall's τ p-value** | 0.0253 | 0.0076 | <0.0001 | 0.0001 |  | 0.0253 | 0.0108 | 0.2069 | 0.0064 |
|  | **Bivariate random-effects estimate (95% CI)** | 0.999 (0.996-1.000) | 0.884 (0.854-0.909) | 0.742 (0.672-0.801) | 0.893 (0.858-0.921) | | 0.999 (0.996-1.000) | 0.904 (0.868-0.931) | 0.825 (0.719-0.897) | 0.987 (0.982-0.990) |
|  | **Deeks' test statistic** | NA | 0.617 | NA | 2.658 |  | NA | 0.628 | 2.725 | NA |
|  | **Deeks' test p-value** | NA | 0.5395 | NA | 0.0160 |  | NA | 0.5441 | 0.0260 | NA |

**Table 6.** Meta-analytic estimates, heterogeneity statistics, and small-study effect assessments stratified by study design.

| **Metric** | | **Study design** | | |
| --- | --- | --- | --- | --- |
|  |  | **DTA** | **MD** | **rDTA** |
| **Accuracy** | **N** | 18 | 11 | 69 |
|  | **Univariate random-effects estimate (95%)** | 0.849 (0.820-0.874) | 0.915 (0.785-0.969) | 0.915 (0.898-0.929) |
|  | **I² (%)** | 63.7% | 98.9% | 94.7% |
|  | **τ²** | 0.0998 | 2.9301 | 0.6106 |
|  | **Cochran's Q test p-value** | 0.0001 | <0.0001 | <0.0001 |
|  | **Kendall's τ** | 0.396 | 0.455 | 0.203 |
|  | **Kendall's τ p-value** | 0.0246 | 0.0602 | 0.0146 |
| **AUC** | **N** | 0 | 0 | 23 |
|  | **Univariate random-effects estimate (95%)** | | | 0.967 (0.951-0.978) |
|  | **I² (%)** |  |  | 90.6% |
|  | **τ²** |  |  | 0.7473 |
|  | **Cochran's Q test p-value** | |  | <0.0001 |
|  | **Kendall's τ** | |  | 0.280 |
|  | **Kendall's τ p-value** | |  | 0.0729 |
| **NPV** | **N** | 18 | 7 | 55 |
|  | **Random-effects estimate (95% CI)** | 0.855 (0.819-0.885) | 0.841 (0.811-0.868) | 0.888 (0.856-0.914) |
|  | **I² (%)** | 53.5% | 0.0% | 94.9% |
|  | **τ²** | 0.1399 | 0.0000 | 0.9658 |
|  | **Cochran's Q test p-value** | 0.0031 | 0.0065 | <0.0001 |
|  | **Kendall's τ** | 0.181 | 0.905 | 0.078 |
|  | **Kendall's τ p-value** | 0.3032 | 0.0028 | 0.4032 |
| **PPV** | **N** | 18 | 7 | 50 |
|  | **Random-effects estimate (95% CI)** | 0.853 (0.803-0.892) | 0.756 (0.402-0.934) | 0.927 (0.901-0.947) |
|  | **I² (%)** | 72.7% | 98.2% | 88.2% |
|  | **τ²** | 0.2985 | 3.8756 | 1.1267 |
|  | **Cochran's Q test p-value** | 0.0001 | <0.0001 | <0.0001 |
|  | **Kendall's τ** | 0.444 | 1.000 | 0.409 |
|  | **Kendall's τ p-value** | 0.0109 | 0.0004 | <0.0001 |
| **Sensitivity** | **N** | 18 | 7 | 55 |
|  | **Univariate random-effects estimate (95%)** | 0.844 (0.789-0.887) | 0.705 (0.653-0.751) | 0.874 (0.795-0.926) |
|  | **I² (%)** | 75.4% | 0.0% | 96.7% |
|  | **τ²** | 0.3905 | 0.0000 | 4.4527 |
|  | **Cochran's Q test p-value** | <0.0001 | 0.0009 | <0.0001 |
|  | **Kendall's τ** | 0.197 | 1.000 | 0.122 |
|  | **Kendall's τ p-value** | 0.2551 | 0.0021 | 0.1975 |
|  | **Bivariate random-effects estimate (95% CI)** | 0.838 (0.784-0.881) | 0.852 (0.563-0.962) | 0.858 (0.767-0.917) |
|  | **Deeks' test statistic** | 2.494 | NA | -3.660 |
|  | **Deeks' test p-value** | 0.0240 | NA | 0.0006 |
| **Specificity** | **N** | 18 | 7 | 55 |
|  | **Univariate random-effects estimate (95%)** | 0.853 (0.820-0.881) | 0.723 (0.689-0.755) | 0.926 (0.886-0.952) |
|  | **I² (%)** | 45.1% | 0.0% | 94.2% |
|  | **τ²** | 0.0881 | 0.0000 | 2.6884 |
|  | **Cochran's Q test p-value** | 0.0604 | 0.0002 | <0.0001 |
|  | **Kendall's τ** | 0.479 | 1.000 | 0.373 |
|  | **Kendall's τ p-value** | 0.0072 | 0.0004 | 0.0001 |
|  | **Bivariate random-effects estimate (95% CI)** | 0.842 (0.813-0.868) | 0.866 (0.599-0.966) | 0.924 (0.879-0.953) |
|  | **Deeks' test statistic** | 2.494 | NA | -3.660 |
|  | **Deeks' test p-value** | 0.0240 | NA | 0.0006 |

**Fig 1.** Forest plot of the random effect model assessing the AUC.

**Fig 2.** Forest plot of the random-effects model assessing the accuracy.

**Fig 3.** Forest plot of the bivariate random-effects model assessing the sensitivity.

**Fig 4.** Forest plot of the bivariate random-effects model assessing the specificity.

**Fig 5.** Forest plot of the random-effects model assessing the NPV.

**Fig 6.** Forest plot of the random-effects model assessing the PPV.

**Fig 7.** Forest plots of the random-effects AUC meta-analysis stratified by AI/ML model family: (a) classical machine learning, (b) tree-ensemble models, and (c) deep-learning models.

**a)**

**b)**

**c)**

**Fig 8.** Forest plots of the random-effects accuracy meta-analysis stratified by AI/ML model family: (a) classical machine learning, (b) tree-ensemble models, and (c) deep-learning models.

**a)**

**b)**

**c)**

**Fig 9.** Forest plots of sensitivity estimates from the bivariate random-effects analysis stratified by AI/ML model family: (a) classical machine learning, (b) tree-ensemble models, and (c) deep-learning models.

**a)**

**b)**

**c)**

**Fig 10.** Forest plots of specificity estimates from the bivariate random-effects analysis stratified by AI/ML model family: (a) classical machine learning, (b) tree-ensemble models, and (c) deep-learning models.

**a)**

**b)**

**c)**

**Fig 11.** Forest plots of NPV estimates from random-effects meta-analyses stratified by AI/ML model family: (a) classical machine learning, (b) tree-ensemble models, and (c) deep-learning models.

**a)**

**b)**

**c)**

**Fig 12.** Forest plots of PPV estimates from random-effects meta-analyses stratified by AI/ML model family: (a) classical machine learning, (b) tree-ensemble models, and (c) deep-learning models.

**a)**

**b)**

**c)**

**Fig 13. Funnel plots for univariate diagnostic performance metrics.**
Funnel plots for accuracy, AUC, positive predictive value (PPV), and negative predictive value (NPV), presented for all models and stratified by AI/ML model category (classical machine learning, deep learning, and tree-ensemble models). Funnel plot asymmetry was assessed using Kendall’s rank correlation test.

**Fig 14. Deeks’ funnel plots for diagnostic accuracy analyses.**
Deeks’ funnel plots for the bivariate sensitivity–specificity analyses, presented for all models and stratified by AI/ML model category (classical machine learning, deep learning, and tree-ensemble models). The corresponding Deeks’ test p-values are reported within each panel.
