# Supplementary material for: Diagnostic performance of artificial intelligence in early diagnosis of mosquito-borne viral infections: Systematic review and meta-analysis
Source: PLOS Digit Health. 2026 Oct 27;5(10):e0001796. doi: 10.1371/journal.pdig.0001796 (PMC13712693; doi:10.1371/journal.pdig.0001796)
Supplement: Supplementary file 4 [file pdig.0001796.s004.docx]

Padua, 27 August 2026

Dear Editor and Reviewers,

We sincerely thank you for your careful and constructive evaluation of our manuscript. We greatly appreciate the detailed methodological comments and suggestions, which have helped us to substantially improve the clarity, transparency, and methodological rigor of the study.

In response to the comments received, we have undertaken a comprehensive revision of the manuscript and, in particular, a substantial re-evaluation of the quantitative synthesis. This included a detailed audit of the extracted data, correction of identified inconsistencies, revision of the statistical methodology, and reanalysis of the meta-analytic component using more appropriate methods for diagnostic accuracy outcomes. We also revised the study eligibility assessment, model classification, risk-of-bias assessment, subgroup analyses, and interpretation of the findings, and updated the corresponding tables, figures, and Supplementary Material throughout.

All analyses affected by these methodological revisions were rerun using the revised dataset and analytical framework, and the manuscript was carefully checked to ensure consistency across the Methods, Results, tables, figures, and Supplementary Material.

Below, we provide a point-by-point response to each comment and indicate the corresponding changes made in the revised manuscript.

**Reviewer #1:**

**1.**Dear Authors,
Thank you for the opportunity to review this manuscript. Overall, the study is well prepared, scientifically sound, and appears to follow the relevant methodological guidelines and reporting standards. The topic is timely and of potential interest to the readership.

I have only a few suggestions that may further strengthen the manuscript:

The authors may wish to consider citing and discussing the following recent publication, which could provide additional methodological context and support for the study design:
DOI: 10.1016/j.identj.2025.109322
Given the increasing importance of AI-specific reporting standards, I encourage the authors to review the PRISMA-AI framework and consider whether its principles can be incorporated into the methodology and reporting of the review.
The Introduction could be further enriched by citing relevant examples of successful machine learning and deep learning applications in healthcare and medical sciences. The following publication may provide useful background information and broader context:
DOI: 10.1186/s12911-026-03427-y

**R:** We sincerely thank the Reviewer for these valuable suggestions. We carefully reviewed the recommended publications and incorporated them where appropriate. Specifically, we have revised the Methods section to acknowledge the relevance of emerging AI-specific reporting recommendations, including the PRISMA-AI framework, as an important reference to improve the transparency and reporting of AI-based systematic reviews. In addition, we have expanded the Discussion by referring to recent evidence from other medical AI applications, highlighting that high predictive performance alone is insufficient to support clinical implementation without rigorous external validation, methodological transparency, and model interpretability. This broader perspective reinforces one of the key conclusions of our review regarding the current limitations of AI models for arboviral infection diagnosis.

**2.** Although the manuscript is generally well structured, the inclusion of a formal quality assessment and risk-of-bias evaluation would substantially strengthen the methodological rigor and reliability of the findings. If such assessments were not performed, the authors should provide a clear justification and acknowledge this as a limitation of the study.

Overall, I believe the manuscript has merit and can be further improved by addressing the above recommendations.

**R:** We thank the Reviewer for this valuable comment. A formal methodological quality and risk-of-bias assessment was conducted for all included studies using the Prediction Model Risk of Bias Assessment Tool (PROBAST), as described in the Methods section. To further improve the transparency and completeness of our assessment, we have revised the manuscript by including the applicability assessment, correcting the presentation of the PROBAST results, and providing the individual domain-level judgements for each included study in the Supplementary Material. We believe these additions further strengthen the methodological rigor and reliability of our systematic review.

**Reviewer #2:**

The authors performed a systematic review and meta-analysis to assess the diagnostic performance of artificial intelligence (AI) in early diagnosis of mosquito-borne viral infections. The topic is worth to investigate, but there are some things I would like to highlight:

**1.** Please define all abbreviations in their full terms at the first occurrence in the text. Check: AUC, PPV, NPV, PROBAST, RT-PCR, NS1, RMSE, MAE, MSE, BRBES, etc.

**R:** We thank the Reviewer for this helpful suggestion. We have carefully reviewed the entire manuscript and ensured that all abbreviations are defined in full at their first occurrence in the text. This includes, but is not limited to, AUC, PPV, NPV, PROBAST, RT-PCR, NS1, RMSE, MAE, MSE, and BRBES. We also performed a comprehensive check to ensure consistent use of abbreviations throughout the manuscript, tables, figures, and supplementary material.

**2.** In the Abstract, please add the information that 4,531 records are the identified records.

**R:** We thank the Reviewer for this helpful suggestion. The Abstract has been revised to clarify that 4,531 records were initially identified through the database search, thereby improving the transparency of the study selection process.

**3.** The authors classified AI models into 5 categories, but only 3 were further discussed and analysed in the paper. Please add justification.

**R:** We thank the Reviewer for this helpful observation. Following the revised classification framework, individual models are now assigned to four methodological families: classical machine learning, tree-ensemble methods, deep learning, and heuristic approaches. Quantitative subgroup comparisons were feasible for classical ML, tree-ensemble, and deep-learning models, whereas the heuristic family was represented by only one study and was therefore described narratively rather than pooled as a separate subgroup. Studies evaluating multiple standalone algorithms are labelled “Multiple AI/ML approaches” only at the descriptive study level. The Methods, Results, Table 2, and Discussion have been revised accordingly.

**4.** It seems that the ‘Hybrid’ model classification is not suitable to be used here. Included studies that were classified as hybrid are using different models from different groups (e.g., kNN, RF, ANN and assessed them separately), but they did not introduce/propose a new hybrid model itself. Please consider using a different term or simply put them into other available categories.

**R:** We thank the Reviewer for this important observation. We agree that the original use of the term “Hybrid” at the study level could conflate genuinely integrated architectures with studies that simply evaluated multiple standalone algorithms. We therefore revised the classification and removed “Hybrid” as a study-level category. Studies evaluating multiple standalone algorithms are now described as “Multiple AI/ML approaches” in the descriptive tables, while each individual model is assigned to its corresponding methodological family (classical ML, tree-ensemble, deep learning, or heuristic) in the quantitative analyses. The Methods, Table 2, Results, and Supplementary Material have been revised accordingly.

**5.** What do the authors mean by ‘When the SD was not directly provided, it was derived from the confusion matrix’ (lines 200-201)? How did the authors do that?

**R:** We thank the reviewer for pointing this out. We apologize for the lack of clarity in the original wording. When the SE was not directly reported, we extracted the numbers of true positives (TP), true negatives (TN), false positives (FP), and false negatives (FN) from the confusion matrix. For sensitivity/recall, specificity, PPV/precision, NPV, and accuracy, we calculated the corresponding performance measure and estimated its SE assuming a binomial distribution. For AUC, when the SE was not directly reported, we estimated it using the se_auc function from the R package auctestr, which implements the method of Hanley and McNeil, based on the numbers of positive (P) and negative (N) observations.

Following the reviewers' suggestions, we substantially revised our statistical approach (see our detailed responses to the related comments below). For sensitivity, specificity, accuracy, PPV, and NPV, no separate SD/SE derivation step is required, with pooling now performed directly on the raw counts of the confusion matrix. The only metric for which a standard error must still be derived when not directly reported is AUC. We have revised the Methods section accordingly, restricting this description to AUC alone and correcting the original wording (which erroneously referred to "SD" rather than "SE").

**6.** Please recheck statements in lines 224-226 for clarity. Make sure that the explanations conform to the PRISMA flow diagram (Fig.1).

**R:** We thank the Reviewer for this helpful observation. We have carefully revised the corresponding statements in the Results section to improve clarity and ensure complete consistency with the PRISMA flow diagram (Figure 1). The study selection process is now described using terminology and numbers that fully correspond to the PRISMA flow diagram.

**7**. Please make sure to add all abbreviations used in Table 1 (in the paper) and Tables 2-3 (in the supplementary file) under the tables. Check, NA for example.

**R:** We thank the Reviewer for this helpful suggestion. We carefully reviewed the abbreviations used in Table 1, Table 2, and in Tables 2–3 of the Supplementary Material and added or completed the corresponding definitions below each table, including the definition of “NA”. Abbreviations are now defined consistently across the manuscript and Supplementary Material.

**8.** Why did the authors include ‘entomological’ and ‘imaging’ as predictor feature categories across included studies when none actually used them (lines 335-336)? Similarly, Fig.3 shows no study used them but Fig.4 still shows them.

**R:** We thank the Reviewer for highlighting this point. We agree that the original terminology did not sufficiently distinguish conventional medical imaging from microscopy-based image inputs. We have therefore revised the coding and the corresponding text. The “imaging” category in Figure 3 originally referred specifically to conventional medical imaging modalities, such as CT, MRI, radiography, or ultrasound, none of which were used by the included studies; this category has therefore been removed from Figure 3. Conversely, digitised blood-smear and microscopy images used in some included studies are now explicitly described as image-based/microscopy inputs and remain represented in the conceptual overview in Figure 4. The entomological category was retained as an a priori predictor category and is explicitly reported as not represented in the included evidence, thereby highlighting a relevant evidence gap.

Figure 3 was revised by removing “imaging”, while “entomological” was retained.

**9.** Why did the authors not include the ‘deep learning’ model category in the forest plot analysis for NPV and PPV (Figs.11 and 12 supplementary file)?

**R:** We thank the reviewer for pointing this out. In the original analysis, only studies explicitly reporting PPV and NPV values were eligible for inclusion, which left no Deep Learning studies to estimate for these two metrics. Following the revised statistical approach (see our response to comments 1–2 and 5), PPV and NPV are now derived directly from the confusion matrix (TP, TN, FP, FN) whenever these counts are available, rather than relying solely on values explicitly reported by the original studies. This substantially increased the number of eligible studies for both metrics, and the Deep Learning category now meets the minimum threshold for inclusion. We have updated the Supplementary File.

**10.** What do the authors mean by ‘Sensitivity estimates covered a broad interval, …, reflecting differences in cross-validation versus hold-out evaluation, …’ (lines 392-393)? What is the relationship between sensitivity with CV vs hold-out evaluation?

**R:** We thank the Reviewer for this important observation. We agree that the original wording implied a direct relationship between sensitivity estimates and the use of cross-validation versus hold-out validation, which was not formally evaluated in our review. We have therefore revised the Discussion to avoid attributing the observed variability in sensitivity to specific validation strategies. The revised text now states that the broad range of sensitivity estimates likely reflects the overall methodological and clinical heterogeneity among the included studies, including differences in study populations, predictor variables, model development, validation approaches, and outcome definitions.

**11.** Please recheck the explanations in lines 479-480 (comparable ES), line 485 (improved ES), line 497 (slightly lower ES), and line 502 (higher ES) for correctness.

**R:** We thank the Reviewer for this careful observation. We have reviewed the corresponding statements throughout the Discussion and revised the wording to ensure that all comparisons accurately reflect the results of the subgroup analyses. Specifically, expressions implying superiority (e.g., “improved”, “higher”, “lower”, or “comparable” effect size) have been replaced with more descriptive language based on the observed pooled estimates, avoiding interpretations that are not directly supported by formal statistical comparisons.

**12**. It seems that the authors did not perform a sensitivity analysis as described in sub-section 3.8.2. It is more likely a ‘subgroup analysis’ rather than a ‘sensitivity analysis’. Please consider changing the term.

**R:** We thank the Reviewer for this important methodological observation. We agree that the analyses presented in Section 3.8.2 are more appropriately described as subgroup analyses rather than sensitivity analyses, as they compare pooled estimates across predefined AI model categories rather than evaluating the robustness of the overall findings under different analytical assumptions. Accordingly, we have revised the terminology throughout the manuscript, including the corresponding section heading and all related references. In addition, as recommended by other reviewers, we expanded the subgroup analysis to include further stratifications beyond AI model category (by virus, geographic location, and study design) and have reported the corresponding pooled estimates in the revised Results and Supplementary File.

**13.** Please recheck if the authors really mean the ‘decision tree-based’ models vs ‘classical ML’ in line 533.
Thank you.

**R:** We thank the Reviewer for identifying this inconsistency. After rechecking the predefined model classification and the corresponding subgroup analysis, we confirmed that the intended category was classical machine learning, rather than “decision tree-based models.” The wording has therefore been corrected. We also rechecked the assignment of individual algorithms to the predefined AI categories throughout the dataset, tables, subgroup analyses, and supplementary material to ensure consistent classification.

**Reviewer #3**:

Overall assessment
This manuscript presents a timely, well-structured, and highly relevant systematic review and meta-analysis on the use of artificial intelligence and machine learning models for the early diagnosis of mosquito-borne viral infections. The topic is important for both clinical medicine and public health,
particularly in settings where rapid laboratory confirmation is unavailable or delayed. The review addresses a clear evidence gap by synthesizing diagnostic performance across a growing but heterogeneous body of literature, and it offers a useful benchmark for future work in the field.
Overall, the manuscript is strong. The search strategy is broad, the eligibility criteria are clearly defined, the review follows PRISMA guidance, and the protocol registration strengthens methodological transparency. The authors should also be commended for using PROBAST to assess risk of bias and for attempting quantitative synthesis across multiple diagnostic performance metrics, including AUC, accuracy, sensitivity, specificity, PPV, and NPV. The paper’s major
contribution is that it moves the field beyond narrative claims about the promise of AI and provides a structured overview of where the evidence is currently strong, where it is fragile, and where future validation is needed.
The manuscript is therefore suitable for publication after revision. My comments below are intended to help the authors sharpen the interpretation of the findings and improve the clinical and translational value of the paper.

**R:** We sincerely thank the Reviewer for the thorough evaluation of our manuscript and for the positive assessment of its novelty, methodological rigor, and potential relevance to both clinical medicine and public health.

Major comments
1. The practical and clinical relevance of the findings should be examined more deeply. The paper reports high pooled diagnostic performance across several metrics, which is encouraging. However, the practical and clinical relevance of these findings remains somewhat underdeveloped.
Many included models appear to be retrospective, research-stage prototypes, and no study reports routine clinical deployment. This creates an important gap between laboratory-style performance and real-world clinical utility.
The authors acknowledge this limitation, but the discussion would benefit from a more explicit analysis of what is required before these models can meaningfully support clinical triage or diagnostic decision-making. For example, the manuscript could more clearly distinguish between discrimination in retrospective datasets and operational usefulness in real clinical workflows. A model with high AUC may still be clinically unsuitable if it is poorly calibrated, difficult to integrate into existing workflows, dependent on unavailable predictors, or not evaluated at clinically meaningful thresholds. I recommend that the authors expand the discussion of clinical utility by addressing issues such as intended use, decision thresholds, workflow integration, turnaround time, availability of required
input variables, prospective validation, cost-effectiveness, and potential consequences of false positives and false negatives.

**R:** We sincerely thank the Reviewer for this insightful comment. We agree that high diagnostic performance alone does not necessarily translate into clinical utility or readiness for implementation. In response, we have expanded the Discussion to better distinguish between retrospective model discrimination and real-world clinical applicability. Specifically, we now emphasize that successful implementation requires not only high predictive performance but also appropriate calibration, prospective and external validation, integration into existing clinical workflows, availability of routinely collected input variables, clinically meaningful decision thresholds, and evaluation of cost-effectiveness and implementation feasibility. We also discuss the potential clinical implications of false-positive and false-negative predictions, highlighting the need for rigorous evaluation before widespread adoption in clinical or public health settings.

**2.** The geographic skew of the evidence base has important implications that should be discussed more substantively. The manuscript shows that 38 of 47 studies, corresponding to 80.9%, were conducted in Asia, while
Africa and Latin America are much less represented. In addition, dengue accounts for 42 of 47 studies, corresponding to 89.3% of the included evidence. This imbalance is important and should be interpreted more strongly.
The current discussion correctly identifies geographic imbalance as a limitation, but it does not fully explore what this means for model transportability and clinical deployment. AI models trained or validated primarily on Asian hospital-based dengue datasets may not perform similarly in African or Latin American settings, where disease prevalence, co-infections, healthcare access, laboratory
availability, population structure, vector ecology, prior immunity, and clinical case-mix may differ substantially. This is not simply a matter of representativeness. It directly affects whether pooled estimates can be
interpreted as evidence of global applicability. I suggest that the authors explicitly discuss the possibility that the pooled performance estimates may conceal clinically important failures in under- represented regions. This is especially relevant for Africa and Latin America, where arboviral burden is substantial but the available AI diagnostic evidence remains limited.

**R:** We sincerely thank the Reviewer for this thoughtful and important comment. We agree that the marked geographical concentration of the available evidence has important implications beyond representativeness alone and may substantially affect the transportability and real-world applicability of AI-based diagnostic models. In response, we have expanded the Discussion to emphasize that the predominance of studies conducted in Asia and focused on dengue limits the generalizability of the pooled estimates to underrepresented regions, such as Africa and Latin America, where differences in disease epidemiology, population characteristics, healthcare infrastructure, laboratory availability, co-infections, and clinical case-mix may influence model performance. We now explicitly acknowledge that the pooled estimates should not be interpreted as evidence of global applicability and that prospective external validation across diverse geographical settings is essential before widespread implementation can be recommended. In addition, we performed exploratory subgroup analyses by geographical location (continent and country), which are reported in the S1 Appendix. These analyses were interpreted cautiously because several geographical strata contained relatively few model-level estimates.

**3.** Calibration deserves stronger emphasis
Calibration is reported in only 5 of 47 included studies. This is one of the most important findings of the review and should receive greater emphasis. Calibration is not a secondary technical detail. For diagnostic and triage models, it is central to whether predicted probabilities correspond to actual event rates.
A model with excellent AUC but poor calibration may rank patients correctly on average while still producing misleading absolute risk estimates. This is particularly problematic if the model is deployed in populations with different disease prevalence from the training or validation datasets. In
triage contexts, miscalibrated probabilities may lead to inappropriate reassurance, unnecessary testing, missed cases, or inefficient use of scarce resources. The manuscript currently notes the scarcity of calibration reporting, but I recommend making this a more prominent interpretive point in the discussion and conclusion. The authors could also recommend that future studies report calibration plots, calibration intercept and slope, Brier score, and decision-curve analysis where appropriate.

**R:** We sincerely thank the Reviewer for this insightful comment. We agree that calibration is a fundamental aspect of prediction model performance and deserves greater emphasis. In response, we have expanded both the Discussion and the Conclusion to highlight that the limited reporting of calibration substantially restricts the interpretation and clinical applicability of the identified AI models. We now explicitly discuss the implications of deploying poorly calibrated models in different clinical settings and recommend that future studies routinely report calibration measures, including calibration plots, calibration intercept and slope, Brier score, and, where appropriate, decision-curve analysis, alongside conventional discrimination metrics.

**4.** The scope of the title and abstract should be aligned with the actual evidence base. The eligibility criteria include dengue, Zika, chikungunya, West Nile virus, yellow fever, and Rift Valley fever. However, the included evidence is overwhelmingly about dengue, with limited evidence for Zika and chikungunya and no included studies addressing West Nile virus, yellow fever, or Rift Valley fever.
This absence is important and should be discussed more explicitly. As currently framed, the title and abstract may suggest broader evidence across mosquito-borne viral infections than the included studies actually support. The authors should either narrow the language in the title and abstract or clearly state that the quantitative findings are driven predominantly by dengue studies and cannot be
generalized to West Nile virus, yellow fever, or Rift Valley fever. A sentence in the abstract noting that no eligible studies addressed West Nile virus, yellow fever, or
Rift Valley fever would improve transparency and help readers interpret the scope of the conclusions appropriately.

**R:** We sincerely thank the Reviewer for this important observation. We agree that the available evidence is heavily dominated by studies on dengue, with only a limited number of studies addressing Zika and chikungunya and no eligible studies identified for West Nile virus, yellow fever, or Rift Valley fever. In response, we have revised the Abstract and Discussion to explicitly clarify that the quantitative findings are driven predominantly by dengue studies and should not be generalized to arboviral infections for which no eligible evidence was available. These revisions improve the transparency and interpretation of the review’s scope.

**5.** The interpretation of pooled estimates should be more cautious given extreme
heterogeneity. The manuscript reports very high heterogeneity across pooled analyses, often with I² values of 100%. This substantially limits the interpretability of the pooled estimates. Although the authors acknowledge heterogeneity, the results and discussion sometimes still present the pooled estimates
as if they provide a stable summary of diagnostic performance. I recommend adopting more cautious language throughout. The pooled estimates are useful as
broad descriptive benchmarks, but they should not be interpreted as precise or generalizable estimates of expected model performance. The authors could make clearer that the meta-analysis synthesizes a highly heterogeneous set of model types, study designs, populations, reference standards, predictors, validation strategies, and target conditions.

**R:** We thank the Reviewer for this important observation. We agree that the extreme heterogeneity observed across all pooled analyses requires cautious interpretation of the summary estimates. In response, we have revised the Results and Discussion to clarify that the pooled estimates should be regarded as descriptive indicators summarizing a highly heterogeneous body of evidence rather than precise or universally generalizable estimates of diagnostic performance. We also emphasize that differences in study design, populations, predictors, reference standards, validation strategies, and target conditions contribute substantially to the observed heterogeneity.

**6.** Risk of bias should be more clearly linked to interpretation of performance
The PROBAST assessment is a strength of the manuscript. However, the implications of the risk- of-bias findings could be integrated more strongly into the interpretation of diagnostic performance. Since 32 of 47 studies were judged at high overall risk of bias, high reported performance should be
interpreted with caution. The discussion could explicitly connect high AUCs and near-perfect accuracies to common methodological weaknesses such as small sample sizes, retrospective designs, internal-only validation, possible overfitting, incomplete reporting of missing data handling, and lack of prospective evaluation.

**R:** We thank the Reviewer for this valuable comment. We agree that the implications of the PROBAST findings should be more explicitly linked to the interpretation of the reported diagnostic performance. Accordingly, we have expanded the Discussion to emphasize that the high prevalence of studies at high risk of bias warrants cautious interpretation of the reported AUC and accuracy estimates. We now explicitly highlight that methodological limitations, including retrospective study designs, small sample sizes, reliance on internal validation, limited reporting of missing data handling, and the absence of prospective evaluation, may have contributed to overly optimistic performance estimates.

**REVIEWER 4**

**Overall assessment**

I would like to begin by congratulating the authors on a substantial and timely piece of work. Synthesising 47 studies on AI/ML models for the early diagnosis of mosquito-borne viral infections is no small undertaking, and the effort shows: the search strategy is comprehensive and fully reproducible, the screening and extraction process is sound, the narrative synthesis by model category is clear and well organised, and the use of PROBAST to appraise risk of bias is a genuine strength. The breadth of evidence assembled here has real descriptive value and addresses a question of clear public health relevance.

After a detailed reading of the manuscript, the supplementary material, and all of the figures (including the forest plots and funnel plots in the S1 Appendix), my main reservations concentrate on the meta-analytic component, which is also the manuscript’s principal claim to novelty. I believe the pooled estimates, as currently presented, are affected by several issues of scale, variance estimation, unit of analysis, and publication-bias assessment that need to be addressed before the quantitative synthesis can be considered reliable. I want to stress that these are addressable, and that the underlying dataset and the rest of the manuscript provide a solid foundation to build on. For these reasons I recommend a major revision, centered on reworking the meta-analysis. Below I set out the main points first, with concrete examples drawn from the authors’ own figures, followed by minor points. I hope these comments are read in the constructive spirit in which they are intended.

**Major points**

1. **Mismatch between the statistical method described and the one applied.** Section 2.5 states that the effect size was computed as a standardised mean difference and reported as Cohen’s d. However, the forest plots in the S1 Appendix make clear that what is being pooled are the diagnostic metrics expressed as percentages (the “ES” column shows, for example, AUC values of 99.00, 87.00, 95.00…), and the axes of Figures 6–8 are on a 0–100 scale rather than in units of Cohen’s d. There is therefore an inconsistency between the Methods and what was actually analysed. Pooling proportions bounded in [0,1] requires an appropriate transformation (logit or Freeman-Tukey double-arcsine) before random-effects pooling. In addition, for diagnostic tests reporting paired sensitivity and specificity, the current methodological standard is the bivariate or HSROC model (Reitsma; Rutter-Gatsonis), which preserves the correlation between the two metrics and the threshold dependency; pooling them separately, as done here, is not ideal. I would ask the authors to clarify precisely what was computed and, where needed, to rerun the meta-analysis using methods appropriate for proportions and diagnostic accuracy.

**R:** We thank the Reviewer for this detailed and constructive assessment. We agree with all points raised and have substantially revised our statistical approach accordingly. In line with the Reviewer's recommendation, accuracy, PPV, NPV, and AUC were pooled on the logit scale using appropriate variance estimation for bounded proportions (R package metafor), rather than as standardized mean differences. Accuracy, PPV, and NPV variances were derived from exact binomial counts extracted from the confusion matrix; AUC variance was derived from the reported or estimated standard error (R package auctestr, based on the method of Hanley and McNeil). Random-effects (REML) models were fitted for the univariate metrics, and pooled estimates were back-transformed to the original 0–1 scale for reporting.

For sensitivity and specificity, we adopted a bivariate random-effects model (Reitsma/Rutter-Gatsonis framework; R package mada), which jointly models the logit-transformed sensitivity and false positive rate (1–specificity) and explicitly accounts for their correlation and threshold dependency across studies. Summary ROC (sROC) curves and the corresponding summary AUC (sAUC) were derived from this model. Pooled sensitivity and specificity estimates reported in the revised manuscript are those obtained from this bivariate model; univariate logit-transformed estimates are reported only as descriptive heterogeneity indicators and are not used as the primary pooled estimate.

All figures, Methods and Results sections have been revised accordingly to accurately reflect these analyses.

2. A demonstrable unit error contaminating the AUC pool. In the random-effects AUC forest plot (S1 Appendix, Fig 1b), the study Tanner L DT 2008 is entered with an effect size of 0.88 (CI 0.81–0.95), whereas every other study is on the 0–100 scale (e.g., 99.00, 95.00, 81.00). In other words, an AUC of 0.88 was not rescaled to 88.00. This single value is the extreme point that appears next to 0 in Figure 6 (AUC panel) and in the funnel plots (Figures 7–8), and it distorts the pooled estimate, the funnel shape, and the Egger test. Notably, in the accuracy forest plot (Fig 2b) the same Tanner study appears correctly as 84.00, which confirms this is a scale error specific to the AUC metric. I would encourage a cell-by-cell audit of the extraction sheet to identify and correct any remaining unit inconsistencies before re-pooling.

**R:** We thank the reviewer for identifying this error. We confirm that the AUC (and CI) values for Tanner L DT (2008) were incorrectly scaled/imported into the meta-analysis software. We also performed a cell-by-cell audit of the extraction and analysis datasets to identify potential inconsistencies in the scale or units of the extracted performance measures. No further discrepancies were identified. We have repeated the meta-analysis with the correct input. All affected analyses and figures, including the AUC forest plot, pooled estimates, funnel plots, and tests, have been updated accordingly.

3. **Implausible per-study confidence intervals, pointing to an error in variance estimation.** The supplementary forest plots show degenerate or near-zero intervals: for example, Das S NN 2025 with ES 99.90 (CI 99.90–99.90) and N=90, or Abdualgalil B 2022 with ES 99.00 (CI 98.99–99.01) and N=6694. An AUC or accuracy around 0.99, with sample sizes of tens or a few thousand, cannot have a confidence interval of essentially zero width. This explains the zero-width intervals seen in the fixed-effect models in the main text (e.g., AUC 98.46, CI 98.46–98.46; accuracy 99.59, CI 99.59–99.59) and indicates that the issue is not limited to model choice: the within-study precision (standard error) appears to have been computed incorrectly. I would ask that the variance computation be reviewed.

**R:** We thank the Reviewer for identifying this issue. We confirm that this arose from an error in how the standard errors were entered into the meta-analysis software used in the original analysis, rather than in their calculation. With the revised methodology, confidence intervals are now appropriately wide and consistent with the underlying sample sizes, confirming that the standard errors themselves had been correctly estimated.

4. **Inapplicable fixed-effect models and a non-interpretable publication-bias assessment.** With I²=100% across all outcomes, the fixed-effect model should not be presented as a valid estimate, and the zero-width intervals it produces confirm this. The funnel plots (Figures 7–8), for their part, have the “effect size” axis on a percentage scale (0–120) and include points at zero and negative values, which are impossible for AUC, sensitivity, or PPV; this explains the implausibly large Egger intercepts reported in the text (e.g., −276.89 for AUC, −189.57 for accuracy). As it stands, the publication-bias analysis cannot be interpreted. I would recommend removing the fixed-effect estimates, recomputing on the correct transformed scale, and reconsidering the suitability of the Egger test (which, in any case, has limited power with fewer than ten studies per outcome).

**R:** We thank the Reviewer for this observation. We confirm that this stemmed from the same underlying issues described in our responses to the previous comments. This has been resolved with the revised data entry and methodology. Funnel plots are now correctly bounded and consistent with the underlying data; we display them on the logit scale but verified consistency with the back-transformed values on the original 0–1 range.
Regarding publication bias, we agree that Egger's test has important limitations for diagnostic accuracy outcomes and, as also raised by another reviewer, is not the recommended approach for paired sensitivity/specificity data. We therefore replaced Egger's test with Deeks' funnel plot asymmetry test for sensitivity and specificity (R package meta), and used Kendall's rank correlation test as a more robust alternative to Egger's for accuracy, PPV, NPV, and AUC.
In addition, as recommended by the Reviewer, fixed-effect pooled estimates have been removed from the revised manuscript and Supplementary summary tables. Given the substantial between-study heterogeneity, random-effects estimates are now used throughout as the primary pooled estimates.

5. **Calibrating the conclusions to the strength of the evidence.** The analysis itself documents that 68% of studies are at high overall risk of bias (PROBAST), that heterogeneity is extreme (I²=100%) across all outcomes, that external validation and calibration are infrequent, and that there is possible publication bias for accuracy and sensitivity. Given this, the tone of the abstract and discussion (“high diagnostic performance”, “promising”, and the presentation of the pooled value as a “model-agnostic quantitative benchmark”) reads as somewhat more optimistic than the evidence supports. Once the issues above are corrected, I would suggest presenting the pooled estimates explicitly as exploratory descriptive indicators of a highly heterogeneous and potentially biased literature, rather than as a reliable measure of real-world model performance. A measured softening of the claims throughout would, in my view, strengthen the paper.

**R:** We thank the Reviewer for this thoughtful comment. We agree that, given the substantial methodological limitations of the available evidence, including the high overall risk of bias, extreme between-study heterogeneity, limited external validation and calibration, and potential publication bias for some outcomes, our conclusions should be expressed more cautiously. We have therefore revised the Abstract, Discussion, and Conclusions to better reflect the exploratory nature of the pooled estimates. Specifically, we now emphasise that these summary estimates should be interpreted as descriptive indicators of the currently available evidence rather than as definitive estimates of real-world diagnostic performance. We have also softened statements regarding the clinical readiness of current AI models and further highlighted the need for robust external validation, calibration, and higher-quality prospective studies.

6. **Scope of the review question.** The title and objective focus on early diagnosis, but several included studies have prognosis, severity, or serotyping as their outcome: severity prediction (Potts 2010), hospitalisation prediction via the SISA index (Sippy 2020), and infecting-serotype prediction (Daniels 2024). In addition, at least one study is based on a fully synthetic dataset (Mahalakshmi 2019), whose weight in a synthesis of real diagnostic performance is debatable. Mixing early diagnosis with prognosis, severity, and serotyping blurs the PICO question and contributes to the observed heterogeneity. I would ask the authors to justify or reconsider the inclusion of these studies and to define the review question more precisely.

**R:** We sincerely thank the Reviewer for this insightful comment. We agree that maintaining a clear and consistent review question is essential, particularly in a systematic review of AI-based diagnostic models.

Following the Reviewer’s suggestion, we carefully re-evaluated the eligibility of the studies identified as potentially outside the scope of our review. As a result, we excluded the studies by Potts et al., which focused on the early prediction of dengue disease severity, and Sippy et al., whose primary outcome was hospitalization prediction. Although both studies employed AI-based approaches using data collected at the initial patient presentation, their primary objective was prognostic rather than diagnostic, and we therefore agreed that they were not fully aligned with the aim of our review. We also excluded the study by Mahalakshmi et al., as the predictive model was developed and evaluated using a fully synthetic dataset rather than real patient data, making it unsuitable for inclusion in a quantitative synthesis of diagnostic performance in clinical settings. We additionally excluded Bharucha et al. (2023), which focused on Japanese encephalitis and therefore fell outside the predefined target mosquito-borne viral infections of the review.

In contrast, we retained the study by Daniels et al. because its objective was to predict the infecting dengue serotype. Although this represents a diagnostic classification task beyond the initial identification of infection, it remains within the broader diagnostic domain, as the model aims to classify the infecting pathogen rather than predict future clinical outcomes. Therefore, we considered this study to be consistent with the diagnostic scope of the review.

Accordingly, the eligibility criteria have been clarified throughout the manuscript to explicitly distinguish diagnostic models from prognostic models and studies based on synthetic datasets. The study selection flow diagram, study characteristics, and all quantitative analyses have been updated following these changes.

**Minor points**

8. **PRISMA flow inconsistencies between text and figure.** Section 3.1 states “115 articles for full-text examination”, but the arithmetic (1,969 − 1,861 = 108) and Figure 1 itself (“Reports sought for retrieval, n=108”) confirm that the correct figure is 108. The sentence “61 articles were excluded, including 47 records” is also inconsistent: per Figure 1, the eligibility-stage exclusions sum to 54 (32+14+6+2), leaving 47 included. The figure is correct; the text figures should be corrected (“115”→108, and the exclusions sentence).

**R:** We thank the Reviewer for this careful observation. We agree that the original manuscript contained inconsistencies between the text and the PRISMA flow diagram. The number of reports sought for retrieval has been corrected from 115 to 108. In addition, following the reassessment of study eligibility described in our response to Major Comment 6, four studies were excluded from the final evidence base because they did not meet the refined diagnostic eligibility criteria. Consequently, 101 full-text articles were assessed for eligibility, of which 58 were excluded (36 not relevant to the review topic, 14 conference abstracts, 6 review articles, and 2 non-English articles), resulting in 43 studies included in the systematic review. The Results section and PRISMA flow diagram have been updated accordingly and are now fully consistent.

9. **Possible errors in study metadata.** (a) Husin, N.A. (2020) is assigned to Yemen in Table 1, but reference [50] (J Theor Appl Inf Technol) and the authors’ affiliations suggest Malaysia; the country should be verified, as it affects the country count, the phrasing “Yemen [49,50]”, and the map in Figure 2 (where Yemen is shown with 2 studies). (b) The year for Goh, B. differs between Table 1 (2025) and Table 3 of the appendix (2023); please reconcile. (c) The two “Mayrose, H. (2023)” entries correspond to two distinct articles ([29] and [30]); distinguishing them with suffixes (2023a/2023b) would avoid the appearance of duplication. (d) Please verify the spelling of the surname “Sippyid”, likely “Sippy”.

   **R:** We thank the Reviewer for carefully identifying these potential inconsistencies. We rechecked the original publications and harmonized the study metadata throughout the manuscript, tables, figures, and Supplementary Material. Regarding Husin et al. (2020), we verified the original study methods and confirmed that the study population/data were from Yemen; therefore, the country assignment in Table 1 was retained. Although the authors’ affiliations may suggest Malaysia, these do not correspond to the geographical setting of the study population. Regarding Goh et al., the correct publication year is 2025, and this has been standardized throughout the manuscript and Supplementary Material. The two distinct publications by Mayrose et al. published in 2023 have now been consistently distinguished as 2023a and 2023b to avoid any appearance of duplication. Finally, we confirmed that the correct surname is “Sippy”; however, this study was subsequently excluded following reassessment of eligibility because its primary outcome was hospitalization prediction rather than diagnosis. We thank the Reviewer for prompting this additional consistency check.
10. **Contradiction regarding the use of imaging.** The text (lines 335-336) and Figure 3 state that no study incorporated imaging data, yet there are studies based on blood-smear images (Mayrose), and Figure 4 itself highlights “Blood smear images” and “Image- and Signal-based Diagnostics” as a category. The coding of input variables should be reconciled so that the text, Figure 3, and Figure 4 are consistent.

**R:** We thank the reviewer for highlighting this point. The imaging category in Figure 3 originally referred specifically to medical imaging modalities, such as CT, MRI, radiography, or ultrasound. Following the reviewers’ comments, since none of the included studies used these types of data, we removed this category from Figure 3 and from the corresponding text in the manuscript. The “image” category in Figure 4 refers instead to the type of input data provided to the AI model, such as digitised microscopy slides reported in the included studies, rather than to medical imaging. The corresponding statement in the Results section was revised accordingly to remove the potentially misleading reference to imaging-derived features.

11. **Reporting of heterogeneity and presentation of forest plots.** (a) I²=100% is reported identically across all outcomes; it would help to provide the exact I² and τ² (or τ) values for each synthesis, along with prediction intervals, which are more informative than the CI of the pooled estimate when heterogeneity is high. (b) The forest plots include a per-study “Sig. 0.000” column whose meaning (the null hypothesis being tested) is unclear; I would suggest removing or explaining it.

**R:** We thank the Reviewer for this observation, we improved results reporting as suggested. We now report the exact I², τ², and corresponding Q-test p-value for each synthesis in a summary results table, together with the statistic and p-value of the recommended publication-bias test for each metric. I² remains consistently high (>80%) across analyses but now varies between syntheses. We also updated and simplified the forest plots.

12. **Reporting and appraisal frameworks.** As this is a review of the diagnostic accuracy of prediction models, it would be more appropriate to align the reporting with PRISMA-DTA (in addition to PRISMA 2020) and to consider TRIPOD+AI as a reference framework for appraising the primary studies. The use of PROBAST is appropriate, but I would recommend: (a) correcting the Figure 5 legend, which mentions “five domains” whereas PROBAST has four domains (participants, predictors, outcome, analysis) plus the overall judgement; (b) also including the PROBAST applicability component; and (c) providing the per-study, per-domain judgements in supplementary material, not only the aggregate plot.

**R:** We sincerely thank the Reviewer for these valuable suggestions.

We agree that reporting guidance specifically developed for diagnostic accuracy reviews provides useful recommendations for improving the transparency of systematic reviews in this field. Accordingly, we have revised the manuscript to further align the reporting with the principles of PRISMA-DTA, while maintaining compliance with PRISMA 2020.

We also acknowledge the relevance of TRIPOD+AI as an important reporting framework for AI-based prediction models. However, as TRIPOD+AI is primarily intended to guide the reporting of primary prediction model studies rather than the conduct or appraisal of systematic reviews, we have cited it in the Discussion as a recommended framework for future model development and reporting, rather than using it as a formal quality assessment tool in the present review.

Regarding the PROBAST assessment, we thank the Reviewer for identifying the inconsistency in the figure legend. The legend has been corrected to reflect the four PROBAST domains (participants, predictors, outcome, and analysis) together with the overall risk-of-bias judgement. In addition, we have expanded the Supplementary Material to include the individual PROBAST domain assessments for each included study.
The study-level PROBAST assessments, including the corresponding applicability judgements for participants, predictors, and outcome, are now reported in the Supplementary Material to improve transparency and reproducibility.

13. **Data availability.** The current statement (“the extracted dataset and generated code are available from the corresponding author upon reasonable request”) does not align with the PLOS data policy, which requires unrestricted availability except where there is a justified legal or ethical restriction. As this is a systematic review without individual patient data, there appears to be no obstacle to depositing the extraction sheet, the full dataset, and the analysis code in a public repository (e.g., OSF, Zenodo, or figshare) with a corresponding DOI. I would warmly encourage public deposition before acceptance; it would also allow the corrections in points 1–5 to be verified.

**R:** We thank the Reviewer for this valuable suggestion. We agree that unrestricted access to the data underlying this systematic review enhances transparency, reproducibility, and compliance with the PLOS Data Availability Policy. Accordingly, we have deposited the extracted dataset and study-level data used for the meta-analysis in figshare https://doi.org/10.6084/m9.figshare.33354231). The Data Availability Statement has been updated to include the repository link and DOI, allowing unrestricted access to all materials supporting the findings of this study.

14. **Minor stylistic points.** Some p-values are written without an equals sign (e.g., “p 0.015”, “p 0.038”); please standardise to “p = ”. The appendix figures are cited as “Figure 1 in S1 Appendix”, which can be confused with Figure 1 of the manuscript; the PLOS supplementary-figure numbering (S1 Fig, S2 Fig, etc.) would be clearer. Please also check the consistent spelling of “Sa-Ngamuang/Sa-ngamuang”.

**R:** We thank the Reviewer for these helpful editorial suggestions. We have carefully reviewed the manuscript and implemented all the recommended changes. Specifically, p-values have been reported consistently using the format p = x.xxx throughout the manuscript. References to supplementary figures have been revised to follow the PLOS convention (e.g., S1 Fig, S2 Fig), thereby avoiding ambiguity with figures in the main manuscript. In addition, we verified the spelling of author names throughout the manuscript and corrected the spelling of Sa-Ngamuang to ensure consistency across the text, tables, supplementary material, and reference list.

**Recommendation**

This manuscript addresses a relevant question and contributes a valuable body of evidence, and the authors are to be commended for the scope and care of the work to date. My principal concern is that the meta-analysis, in its current form, is affected by demonstrable errors of scale, variance estimation, and unit of analysis, together with a statistical model that is not well suited to the nature of the metrics and a publication-bias assessment that cannot currently be interpreted.

Addressing these will require reworking the quantitative synthesis with appropriate methods, auditing the extraction sheet, tempering the conclusions, and resolving the numerical and reporting inconsistencies noted above. I am confident these improvements are achievable, and that once implemented the work will make a worthwhile contribution to the literature. I therefore recommend a major revision, and I would be glad to review a revised version.

**Reviewer #5:**

 Many thanks for your invitation. This study conducted to Diagnostic performance of Artificial Intelligence in early diagnosis of mosquito borne viral infections: systematic review and meta-analysis, this is a good manuscript. Minor revision to improve the quality of the article before publication, is helpful.

- All sections must be prepared according to the journal format.

**R:** We thank the Reviewer for this suggestion. We performed a comprehensive formatting and consistency review of the revised manuscript in accordance with the PLOS Digital Health submission guidelines. We checked the manuscript structure, headings, abbreviations, tables and figures, Supporting Information labelling and citations, reference formatting, and consistency of all table and figure numbering and cross-references throughout the main manuscript and Supplementary Material. The PRISMA 2020 checklist was also updated to reflect the final revised manuscript and corresponding line numbers.

- The statistical approach used for pooling diagnostic accuracy outcomes appears inappropriate. Sensitivity, specificity, PPV, NPV, and AUC should not be synthesized using standardized mean differences (Cohen's d). Diagnostic accuracy meta-analyses generally require hierarchical models such as the bivariate random-effects model or HSROC model to account for the correlation between sensitivity and specificity. The authors should justify their analytical approach or reanalyse the data using accepted DTA meta-analysis methodology.

**R:** We thank the Reviewer for this important observation, which aligns with comments raised by another reviewer (see our detailed response above). We agree and have revised our statistical approach accordingly: sensitivity, specificity, accuracy, PPV, and NPV are no longer pooled using Cohen's d. Sensitivity and specificity are now synthesized using a bivariate random-effects model (Reitsma/HSROC framework, R package mada), which accounts for their correlation and threshold dependency; accuracy, PPV, and NPV are pooled using a logit transformation appropriate for bounded proportions; and AUC is pooled on the logit scale. Please see our detailed response to the related comment above for the full description of the revised methodology.

- It would be better if they explained exactly why they chose PROBAST and whether another tool would not be more appropriate for purely diagnostic studies.

**R:** We thank the Reviewer for this important methodological question. PROBAST was selected because the studies included in our review primarily evaluated multivariable diagnostic prediction models, including model development and validation studies, rather than conventional evaluations of a single index test against a reference standard. PROBAST was specifically developed to assess risk of bias in prediction model studies and evaluates four domains that are particularly relevant to AI/ML modelling: participants, predictors, outcome, and analysis. In particular, the analysis domain captures methodological issues such as sample size, overfitting, predictor handling, model development, and validation, which are central to the assessment of AI-based prediction models. By contrast, tools such as QUADAS-2 are primarily designed for conventional diagnostic test accuracy studies and are less specifically tailored to prediction-model development. We have clarified the rationale for selecting PROBAST in the Methods section and have expanded the Supplementary Material to provide study-level domain assessments.

- It is expected that analyses such as: Type of disease (Dengue vs Zika vs Chikungunya), Type of AI algorithm, Type of data (clinical/laboratory), Type of validation, Country, Study design, will be performed as meta-regression or subgroup analysis.

**R:** We thank the Reviewer for this helpful suggestion. We expanded the quantitative synthesis by performing subgroup analyses by AI/ML model family, pathogen, geographical location, and study design where sufficient data were available. Because the evidence base was overwhelmingly focused on dengue, pathogen-specific quantitative synthesis was restricted to a dengue-only analysis; the number of studies addressing Zika and chikungunya was insufficient for meaningful pooling. Additional subgroup analyses by data-source type and validation strategy were not performed because these categories were frequently non-mutually exclusive and/or incompletely reported, which would have produced sparse and poorly interpretable strata. These characteristics are instead summarised descriptively in the manuscript and Supplementary Material. The new subgroup results are presented in Table 3 and in the S1 Appendix.

- For meta-analysis of diagnostic accuracy, the Egger test is not usually recommended.
The standard method is: Deeks' funnel plot asymmetry test.

**R:** We thank the Reviewer for this suggestion. We replaced Egger's test with Deeks' funnel plot asymmetry test for sensitivity and specificity (R package meta), and used Kendall's rank correlation test as a more robust alternative to Egger's for the remaining outcomes (accuracy, PPV, NPV, AUC).

We hope that the revised manuscript now adequately addresses all comments and provides a more rigorous and appropriately cautious synthesis of the available evidence.

Sincerely,
Claudia Cozzolino Cangiano
*on behalf of all authors*
