# Supplementary material for: Diagnostic performance of artificial intelligence in early diagnosis of mosquito-borne viral infections: Systematic review and meta-analysis
Source: PLOS Digit Health. 2026 Oct 27;5(10):e0001796. doi: 10.1371/journal.pdig.0001796 (PMC13712693; doi:10.1371/journal.pdig.0001796)
Supplement: Supplementary file 5 [file pdig.0001796.s003.docx]

**REVIEW COMMENTS TO THE AUTHOR**

Manuscript PDIG-D-26-01019

*Diagnostic performance of Artificial Intelligence in early diagnosis of mosquito-borne viral infections: systematic review and meta-analysis*

**Overall assessment**

I would like to begin by congratulating the authors on a substantial and timely piece of work. Synthesising 47 studies on AI/ML models for the early diagnosis of mosquito-borne viral infections is no small undertaking, and the effort shows: the search strategy is comprehensive and fully reproducible, the screening and extraction process is sound, the narrative synthesis by model category is clear and well organised, and the use of PROBAST to appraise risk of bias is a genuine strength. The breadth of evidence assembled here has real descriptive value and addresses a question of clear public health relevance.

After a detailed reading of the manuscript, the supplementary material, and all of the figures (including the forest plots and funnel plots in the S1 Appendix), my main reservations concentrate on the meta-analytic component, which is also the manuscript’s principal claim to novelty. I believe the pooled estimates, as currently presented, are affected by several issues of scale, variance estimation, unit of analysis, and publication-bias assessment that need to be addressed before the quantitative synthesis can be considered reliable. I want to stress that these are addressable, and that the underlying dataset and the rest of the manuscript provide a solid foundation to build on. For these reasons I recommend a major revision, centered on reworking the meta-analysis. Below I set out the main points first, with concrete examples drawn from the authors’ own figures, followed by minor points. I hope these comments are read in the constructive spirit in which they are intended.

**Major points**

1. **Mismatch between the statistical method described and the one applied.** Section 2.5 states that the effect size was computed as a standardised mean difference and reported as Cohen’s d. However, the forest plots in the S1 Appendix make clear that what is being pooled are the diagnostic metrics expressed as percentages (the “ES” column shows, for example, AUC values of 99.00, 87.00, 95.00…), and the axes of Figures 6–8 are on a 0–100 scale rather than in units of Cohen’s d. There is therefore an inconsistency between the Methods and what was actually analysed. Pooling proportions bounded in [0,1] requires an appropriate transformation (logit or Freeman-Tukey double-arcsine) before random-effects pooling. In addition, for diagnostic tests reporting paired sensitivity and specificity, the current methodological standard is the bivariate or HSROC model (Reitsma; Rutter-Gatsonis), which preserves the correlation between the two metrics and the threshold dependency; pooling them separately, as done here, is not ideal. I would ask the authors to clarify precisely what was computed and, where needed, to rerun the meta-analysis using methods appropriate for proportions and diagnostic accuracy.
2. **A demonstrable unit error contaminating the AUC pool.** In the random-effects AUC forest plot (S1 Appendix, Fig 1b), the study Tanner L DT 2008 is entered with an effect size of 0.88 (CI 0.81–0.95), whereas every other study is on the 0–100 scale (e.g., 99.00, 95.00, 81.00). In other words, an AUC of 0.88 was not rescaled to 88.00. This single value is the extreme point that appears next to 0 in Figure 6 (AUC panel) and in the funnel plots (Figures 7–8), and it distorts the pooled estimate, the funnel shape, and the Egger test. Notably, in the accuracy forest plot (Fig 2b) the same Tanner study appears correctly as 84.00, which confirms this is a scale error specific to the AUC metric. I would encourage a cell-by-cell audit of the extraction sheet to identify and correct any remaining unit inconsistencies before re-pooling.
3. **Implausible per-study confidence intervals, pointing to an error in variance estimation.** The supplementary forest plots show degenerate or near-zero intervals: for example, Das S NN 2025 with ES 99.90 (CI 99.90–99.90) and N=90, or Abdualgalil B 2022 with ES 99.00 (CI 98.99–99.01) and N=6694. An AUC or accuracy around 0.99, with sample sizes of tens or a few thousand, cannot have a confidence interval of essentially zero width. This explains the zero-width intervals seen in the fixed-effect models in the main text (e.g., AUC 98.46, CI 98.46–98.46; accuracy 99.59, CI 99.59–99.59) and indicates that the issue is not limited to model choice: the within-study precision (standard error) appears to have been computed incorrectly. I would ask that the variance computation be reviewed.
4. **Inapplicable fixed-effect models and a non-interpretable publication-bias assessment.** With I²=100% across all outcomes, the fixed-effect model should not be presented as a valid estimate, and the zero-width intervals it produces confirm this. The funnel plots (Figures 7–8), for their part, have the “effect size” axis on a percentage scale (0–120) and include points at zero and negative values, which are impossible for AUC, sensitivity, or PPV; this explains the implausibly large Egger intercepts reported in the text (e.g., −276.89 for AUC, −189.57 for accuracy). As it stands, the publication-bias analysis cannot be interpreted. I would recommend removing the fixed-effect estimates, recomputing on the correct transformed scale, and reconsidering the suitability of the Egger test (which, in any case, has limited power with fewer than ten studies per outcome).
5. **Calibrating the conclusions to the strength of the evidence.** The analysis itself documents that 68% of studies are at high overall risk of bias (PROBAST), that heterogeneity is extreme (I²=100%) across all outcomes, that external validation and calibration are infrequent, and that there is possible publication bias for accuracy and sensitivity. Given this, the tone of the abstract and discussion (“high diagnostic performance”, “promising”, and the presentation of the pooled value as a “model-agnostic quantitative benchmark”) reads as somewhat more optimistic than the evidence supports. Once the issues above are corrected, I would suggest presenting the pooled estimates explicitly as exploratory descriptive indicators of a highly heterogeneous and potentially biased literature, rather than as a reliable measure of real-world model performance. A measured softening of the claims throughout would, in my view, strengthen the paper.
6. **Scope of the review question.** The title and objective focus on early diagnosis, but several included studies have prognosis, severity, or serotyping as their outcome: severity prediction (Potts 2010), hospitalisation prediction via the SISA index (Sippy 2020), and infecting-serotype prediction (Daniels 2024). In addition, at least one study is based on a fully synthetic dataset (Mahalakshmi 2019), whose weight in a synthesis of real diagnostic performance is debatable. Mixing early diagnosis with prognosis, severity, and serotyping blurs the PICO question and contributes to the observed heterogeneity. I would ask the authors to justify or reconsider the inclusion of these studies and to define the review question more precisely.

**Minor points**

8. **PRISMA flow inconsistencies between text and figure.** Section 3.1 states “115 articles for full-text examination”, but the arithmetic (1,969 − 1,861 = 108) and Figure 1 itself (“Reports sought for retrieval, n=108”) confirm that the correct figure is 108. The sentence “61 articles were excluded, including 47 records” is also inconsistent: per Figure 1, the eligibility-stage exclusions sum to 54 (32+14+6+2), leaving 47 included. The figure is correct; the text figures should be corrected (“115”→108, and the exclusions sentence).
9. **Possible errors in study metadata.** (a) Husin, N.A. (2020) is assigned to Yemen in Table 1, but reference [50] (J Theor Appl Inf Technol) and the authors’ affiliations suggest Malaysia; the country should be verified, as it affects the country count, the phrasing “Yemen [49,50]”, and the map in Figure 2 (where Yemen is shown with 2 studies). (b) The year for Goh, B. differs between Table 1 (2025) and Table 3 of the appendix (2023); please reconcile. (c) The two “Mayrose, H. (2023)” entries correspond to two distinct articles ([29] and [30]); distinguishing them with suffixes (2023a/2023b) would avoid the appearance of duplication. (d) Please verify the spelling of the surname “Sippyid”, likely “Sippy”.
10. **Contradiction regarding the use of imaging.** The text (lines 335-336) and Figure 3 state that no study incorporated imaging data, yet there are studies based on blood-smear images (Mayrose), and Figure 4 itself highlights “Blood smear images” and “Image- and Signal-based Diagnostics” as a category. The coding of input variables should be reconciled so that the text, Figure 3, and Figure 4 are consistent.
11. **Reporting of heterogeneity and presentation of forest plots.** (a) I²=100% is reported identically across all outcomes; it would help to provide the exact I² and τ² (or τ) values for each synthesis, along with prediction intervals, which are more informative than the CI of the pooled estimate when heterogeneity is high. (b) The forest plots include a per-study “Sig. 0.000” column whose meaning (the null hypothesis being tested) is unclear; I would suggest removing or explaining it.
12. **Reporting and appraisal frameworks.** As this is a review of the diagnostic accuracy of prediction models, it would be more appropriate to align the reporting with PRISMA-DTA (in addition to PRISMA 2020) and to consider TRIPOD+AI as a reference framework for appraising the primary studies. The use of PROBAST is appropriate, but I would recommend: (a) correcting the Figure 5 legend, which mentions “five domains” whereas PROBAST has four domains (participants, predictors, outcome, analysis) plus the overall judgement; (b) also including the PROBAST applicability component; and (c) providing the per-study, per-domain judgements in supplementary material, not only the aggregate plot.
13. **Data availability.** The current statement (“the extracted dataset and generated code are available from the corresponding author upon reasonable request”) does not align with the PLOS data policy, which requires unrestricted availability except where there is a justified legal or ethical restriction. As this is a systematic review without individual patient data, there appears to be no obstacle to depositing the extraction sheet, the full dataset, and the analysis code in a public repository (e.g., OSF, Zenodo, or figshare) with a corresponding DOI. I would warmly encourage public deposition before acceptance; it would also allow the corrections in points 1–5 to be verified.
14. **Minor stylistic points.** Some p-values are written without an equals sign (e.g., “p 0.015”, “p 0.038”); please standardise to “p = ”. The appendix figures are cited as “Figure 1 in S1 Appendix”, which can be confused with Figure 1 of the manuscript; the PLOS supplementary-figure numbering (S1 Fig, S2 Fig, etc.) would be clearer. Please also check the consistent spelling of “Sa-Ngamuang/Sa-ngamuang”.

**Recommendation**

This manuscript addresses a relevant question and contributes a valuable body of evidence, and the authors are to be commended for the scope and care of the work to date. My principal concern is that the meta-analysis, in its current form, is affected by demonstrable errors of scale, variance estimation, and unit of analysis, together with a statistical model that is not well suited to the nature of the metrics and a publication-bias assessment that cannot currently be interpreted.

Addressing these will require reworking the quantitative synthesis with appropriate methods, auditing the extraction sheet, tempering the conclusions, and resolving the numerical and reporting inconsistencies noted above. I am confident these improvements are achievable, and that once implemented the work will make a worthwhile contribution to the literature. I therefore recommend a major revision, and I would be glad to review a revised version.
