# Supplementary material for: Diagnostic performance of artificial intelligence in early diagnosis of mosquito-borne viral infections: Systematic review and meta-analysis
Source: PLOS Digit Health. 2026 Oct 27;5(10):e0001796. doi: 10.1371/journal.pdig.0001796 (PMC13712693; doi:10.1371/journal.pdig.0001796)
Supplement: Peer review history

# Peer review: Decision Letter 0

**DOI:** 10.1371/journal.pdig.0001796.r001

**Authors:** Nadav Rappoport, Kaisaierjiang Kadier

2 Jul 2026

PDIG-D-26-01019Diagnostic performance of Artificial Intelligence in early diagnosis of mosquito-borne viral infections: systematic review and meta-analysisPLOS Digital Health Dear Dr. Cozzolino Cangiano, Thank you for submitting your manuscript to PLOS Digital Health. After careful consideration, we feel that it has merit but does not fully meet PLOS Digital Health's publication criteria as it currently stands. Therefore, we invite you to submit a revised version of the manuscript that addresses the points raised during the review process. Please submit your revised manuscript by Aug 31 2026 11:59PM. If you will need more time than this to complete your revisions, please reply to this message or contact the journal office at digitalhealth@plos.org. When you're ready to submit your revision, log on to https://www.editorialmanager.com/pdig/ and select the 'Submissions Needing Revision' folder to locate your manuscript file. Please include the following items when submitting your revised manuscript:* A letter that responds to each point raised by the editor and reviewer(s). You should upload this letter as a separate file labeled 'Response to Reviewers'. This file does not need to include responses to any formatting updates and technical items listed in the 'Journal Requirements' section below.* A marked-up copy of your manuscript that highlights changes made to the original version. You should upload this as a separate file labeled 'Revised Manuscript with Track Changes'.* An unmarked version of your revised paper without tracked changes. You should upload this as a separate file labeled 'Manuscript'. If you would like to make changes to your financial disclosure, competing interests statement, or data availability statement, please make these updates within the submission form at the time of resubmission. Guidelines for resubmitting your figure files are available below the reviewer comments at the end of this letter. As the corresponding author, your ORCID iD is verified in the submission system and will appear in the published article. PLOS supports the use of ORCID, and we encourage all coauthors to register for an ORCID iD and use it as well. Please encourage your coauthors to verify their ORCID iD within the submission system before final acceptance, as unverified ORCID iDs will not appear in the published article. Only the individual author can complete the verification step; PLOS staff cannot verify ORCID iDs on behalf of authors.We look forward to receiving your revised manuscript. Kind regards, Kaisaierjiang KadierGuest EditorPLOS Digital Health Nadav RappoportSection EditorPLOS Digital Health Leo Anthony CeliEditor-in-ChiefPLOS Digital Healthorcid.org/0000-0001-6712-6626 **Journal Requirements:**

1. In the online submission form, you indicated that “All data were obtained from previously published sources cited in the manuscript. The extracted dataset and generated code are available from the corresponding author upon reasonable request.”.

All PLOS journals now require all data underlying the findings described in their manuscript to be freely available to other researchers, either

1. In a public repository,

2. Within the manuscript itself, or

3. Uploaded as supplementary information.

This policy applies to all data except where public deposition would breach compliance with the protocol approved by your research ethics board. If your data cannot be made publicly available for ethical or legal reasons (e.g., public availability would compromise patient privacy), please explain your reasons by return email and your exemption request will be escalated to the editor for approval. Your exemption request will be handled independently and will not hold up the peer review process, but will need to be resolved should your manuscript be accepted for publication. One of the Editorial team will then be in touch if there are any issues.

2. Some material included in your submission may be copyrighted. According to PLOS’s copyright policy, authors who use figures or other material (e.g., graphics, clipart, maps) from another author or copyright holder must demonstrate or obtain permission to publish this material under the Creative Commons Attribution 4.0 International (CC BY 4.0) License used by PLOS journals. Please closely review the details of PLOS’s copyright requirements here: PLOS Licenses and Copyright. If you need to request permissions from a copyright holder, you may use PLOS's Copyright Content Permission form.

Please respond directly to this email or email the journal office and provide any known details concerning your material's license terms and permissions required for reuse, even if you have not yet obtained copyright permissions or are unsure of your material's copyright compatibility.

Potential Copyright Issues:

a. Figure 2: please (a) provide a direct link to the base layer of the map (i.e., the country or region border shape) and ensure this is also included in the figure legend; and (b) provide a link to the terms of use / license information for the base layer image or shapefile. We cannot publish proprietary or copyrighted maps (e.g. Google Maps, Mapquest) and the terms of use for your map base layer must be compatible with our CC-BY 4.0 license.

Note: if you created the map in a software program like R or ArcGIS, please locate and indicate the source of the basemap shapefile onto which data has been plotted.

If your map was obtained from a copyrighted source please amend the figure so that the base map used is from an openly available source. Alternatively, please provide explicit written permission from the copyright holder granting you the right to publish the material under our CC-BY 4.0 license.

Please note that the following CC BY licenses are compatible with PLOS license: CC BY 4.0, CC BY 2.0 and CC BY 3.0, meanwhile such licenses as CC BY-ND 3.0 and others are not compatible due to additional restrictions.

If you are unsure whether you can use a map or not, please do reach out and we will be able to help you. The following websites are good examples of where you can source open access or public domain maps:

* U.S. Geological Survey (USGS) - All maps are in the public domain. (http://www.usgs.gov)

* PlaniGlobe - All maps are published under a Creative Commons license so please cite “PlaniGlobe, http://www.planiglobe.com, CC BY 2.0” in the image credit after the caption. (http://www.planiglobe.com/?lang=enl)

* Natural Earth - All maps are public domain. (http://www.naturalearthdata.com/about/terms-of-use/)

b. Figure 4: Please confirm whether you drew the images / clip-art within the figure panels by hand. If you did not draw the images, please provide (a) a link to the source of the images or icons and their license / terms of use; or (b) written permission from the copyright holder to publish the images or icons under our CC-BY 4.0 license. Alternatively, you may replace the images with open source alternatives. See these open source resources you may use to replace images / clip-art:

- https://commons.wikimedia.org

- https://openclipart.org/

3. As required by our policy on Data Availability, please ensure your manuscript or supplementary information includes the following:

A numbered table of all studies identified in the literature search, including those that were excluded from the analyses.

For every excluded study, the table should list the reason(s) for exclusion.

If any of the included studies are unpublished, include a link (URL) to the primary source or detailed information about how the content can be accessed.

A table of all data extracted from the primary research sources for the systematic review and/or meta-analysis. The table must include the following information for each study:

Name of data extractors and date of data extraction

Confirmation that the study was eligible to be included in the review.

All data extracted from each study for the reported systematic review and/or meta-analysis that would be needed to replicate your analyses.

If data or supporting information were obtained from another source (e.g. correspondence with the author of the original research article), please provide the source of data and dates on which the data/information were obtained by your research group.

If applicable for your analysis, a table showing the completed risk of bias and quality/certainty assessments for each study or outcome.  Please ensure this is provided for each domain or parameter assessed. For example, if you used the Cochrane risk-of-bias tool for randomized trials, provide answers to each of the signalling questions for each study. If you used GRADE to assess certainty of evidence, provide judgements about each of the quality of evidence factor. This should be provided for each outcome.

An explanation of how missing data were handled.

This information can be included in the main text, supplementary information, or relevant data repository. Please note that providing these underlying data is a requirement for publication in this journal, and if these data are not provided your manuscript might be rejected.

If the reviewer comments include a recommendation to cite specific previously published works, please review and evaluate these publications to determine whether they are relevant and should be cited. There is no requirement to cite these works unless the editor has indicated otherwise.  **Additional Editor Comments (if provided):**    [Note: HTML markup is below. Please do not edit.] **Reviewers' Comments:**  Reviewer's Responses to Questions

**Comments to the Author**

1. Does this manuscript meet PLOS Digital Health’s publication criteria? Is the manuscript technically sound, and do the data support the conclusions? The manuscript must describe methodologically and ethically rigorous research with conclusions that are appropriately drawn based on the data presented.

Reviewer #1: Yes

Reviewer #2: Yes

Reviewer #3: Yes

Reviewer #4: Partly

Reviewer #5: Yes

**********

2. Has the statistical analysis been performed appropriately and rigorously?

Reviewer #1: Yes

Reviewer #2: Yes

Reviewer #3: Yes

Reviewer #4: No

Reviewer #5: No

**********

3. Have the authors made all data underlying the findings in their manuscript fully available (please refer to the Data Availability Statement at the start of the manuscript PDF file)?

The PLOS Data policy requires authors to make all data underlying the findings described in their manuscript fully available without restriction, with rare exception. The data should be provided as part of the manuscript or its supporting information, or deposited to a public repository. For example, in addition to summary statistics, the data points behind means, medians and variance measures should be available. If there are restrictions on publicly sharing data—e.g. participant privacy or use of data from a third party—those must be specified.

Reviewer #1: Yes

Reviewer #2: Yes

Reviewer #3: Yes

Reviewer #4: No

Reviewer #5: Yes

**********

4. Is the manuscript presented in an intelligible fashion and written in standard English?

PLOS Digital Health does not copyedit accepted manuscripts, so the language in submitted articles must be clear, correct, and unambiguous. Any typographical or grammatical errors should be corrected at revision, so please note any specific errors here.

Reviewer #1: Yes

Reviewer #2: Yes

Reviewer #3: Yes

Reviewer #4: Yes

Reviewer #5: Yes

**********

5. Review Comments to the Author

Please use the space provided to explain your answers to the questions above. You may also include additional comments for the author, including concerns about dual publication, research ethics, or publication ethics. (Please upload your review as an attachment if it exceeds 20,000 characters)

**Reviewer #1:**  Dear Authors,

Thank you for the opportunity to review this manuscript. Overall, the study is well prepared, scientifically sound, and appears to follow the relevant methodological guidelines and reporting standards. The topic is timely and of potential interest to the readership.

I have only a few suggestions that may further strengthen the manuscript:

The authors may wish to consider citing and discussing the following recent publication, which could provide additional methodological context and support for the study design:

DOI: 10.1016/j.identj.2025.109322

Given the increasing importance of AI-specific reporting standards, I encourage the authors to review the PRISMA-AI framework and consider whether its principles can be incorporated into the methodology and reporting of the review.

The Introduction could be further enriched by citing relevant examples of successful machine learning and deep learning applications in healthcare and medical sciences. The following publication may provide useful background information and broader context:

DOI: 10.1186/s12911-026-03427-y

Although the manuscript is generally well structured, the inclusion of a formal quality assessment and risk-of-bias evaluation would substantially strengthen the methodological rigor and reliability of the findings. If such assessments were not performed, the authors should provide a clear justification and acknowledge this as a limitation of the study.

Overall, I believe the manuscript has merit and can be further improved by addressing the above recommendations.

**Reviewer #2:**  The authors performed a systematic review and meta-analysis to assess the diagnostic performance of artificial intelligence (AI) in early diagnosis of mosquito-borne viral infections. The topic is worth to investigate, but there are some things I would like to highlight:

1. Please define all abbreviations in their full terms at the first occurrence in the text. Check, AUC, PPV, NPV, PROBAST, RT-PCR, NS1, RMSE, MAE, MSE, BRBES, etc.

2. In the Abstract, please add the information that 4,531 records are the identified records.

3. The authors classified AI models into 5 categories, but only 3 were further discussed and analysed in the paper. Please add justification.

4. It seems that the ‘Hybrid’ model classification is not suitable to be used here. Included studies that were classified as hybrid are using different models from different groups (e.g., kNN, RF, ANN and assessed them separately), but they did not introduce/propose a new hybrid model itself. Please consider using a different term or simply put them into other available categories.

5. What do the authors mean by ‘When the SD was not directly provided, it was derived from the confusion matrix’ (lines 200-201)? How did the authors do that?

6. Please recheck statements in lines 224-226 for clarity. Make sure that the explanations conform to the PRISMA flow diagram (Fig.1).

7. Please make sure to add all abbreviations used in Table 1 (in the paper) and Tables 2-3 (in the supplementary file) under the tables. Check, NA for example.

8. Why did the authors include ‘entomological’ and ‘imaging’ as predictor feature categories across included studies when none actually used them (lines 335-336)? Similarly, Fig.3 shows no study used them but Fig.4 still shows them.

9. Why did the authors not include the ‘deep learning’ model category in the forest plot analysis for NPV and PPV (Figs.11 and 12 supplementary file)?

10. What do the authors mean by ‘Sensitivity estimates covered a broad interval, …, reflecting differences in cross-validation versus hold-out evaluation, …’ (lines 392-393)? What is the relationship between sensitivity with CV vs hold-out evaluation?

11. Please recheck the explanations in lines 479-480 (comparable ES), line 485 (improved ES), line 497 (slightly lower ES), and line 502 (higher ES) for correctness.

12. It seems that the authors did not perform a sensitivity analysis as described in sub-section 3.8.2. It is more likely a ‘subgroup analysis’ rather than a ‘sensitivity analysis’. Please consider changing the term.

13. Please recheck if the authors really mean the ‘decision tree-based’ models vs ‘classical ML’ in line 533.

Thank you.

**Reviewer #3** : Overall assessment

This manuscript presents a timely, well-structured, and highly relevant systematic review and meta-

analysis on the use of artificial intelligence and machine learning models for the early diagnosis of

mosquito-borne viral infections. The topic is important for both clinical medicine and public health,

particularly in settings where rapid laboratory confirmation is unavailable or delayed. The review

addresses a clear evidence gap by synthesizing diagnostic performance across a growing but

heterogeneous body of literature, and it offers a useful benchmark for future work in the field.

Overall, the manuscript is strong. The search strategy is broad, the eligibility criteria are clearly

defined, the review follows PRISMA guidance, and the protocol registration strengthens

methodological transparency. The authors should also be commended for using PROBAST to

assess risk of bias and for attempting quantitative synthesis across multiple diagnostic performance

metrics, including AUC, accuracy, sensitivity, specificity, PPV, and NPV. The paper’s major

contribution is that it moves the field beyond narrative claims about the promise of AI and provides

a structured overview of where the evidence is currently strong, where it is fragile, and where future

validation is needed.

The manuscript is therefore suitable for publication after revision. My comments below are

intended to help the authors sharpen the interpretation of the findings and improve the clinical and

translational value of the paper.

Major comments

1. The practical and clinical relevance of the findings should be examined more deeply

The paper reports high pooled diagnostic performance across several metrics, which is encouraging.

However, the practical and clinical relevance of these findings remains somewhat underdeveloped.

Many included models appear to be retrospective, research-stage prototypes, and no study reports

routine clinical deployment. This creates an important gap between laboratory-style performance

and real-world clinical utility.

The authors acknowledge this limitation, but the discussion would benefit from a more explicit

analysis of what is required before these models can meaningfully support clinical triage or

diagnostic decision-making. For example, the manuscript could more clearly distinguish between

discrimination in retrospective datasets and operational usefulness in real clinical workflows. A

model with high AUC may still be clinically unsuitable if it is poorly calibrated, difficult to

integrate into existing workflows, dependent on unavailable predictors, or not evaluated at clinically

meaningful thresholds.

I recommend that the authors expand the discussion of clinical utility by addressing issues such as

intended use, decision thresholds, workflow integration, turnaround time, availability of required

input variables, prospective validation, cost-effectiveness, and potential consequences of false

positives and false negatives.

2. The geographic skew of the evidence base has important implications that should be

discussed more substantively

The manuscript shows that 38 of 47 studies, corresponding to 80.9%, were conducted in Asia, while

Africa and Latin America are much less represented. In addition, dengue accounts for 42 of 47

studies, corresponding to 89.3% of the included evidence. This imbalance is important and should

be interpreted more strongly.

The current discussion correctly identifies geographic imbalance as a limitation, but it does not fully

explore what this means for model transportability and clinical deployment. AI models trained or

validated primarily on Asian hospital-based dengue datasets may not perform similarly in African

or Latin American settings, where disease prevalence, co-infections, healthcare access, laboratory

availability, population structure, vector ecology, prior immunity, and clinical case-mix may differ

substantially.

This is not simply a matter of representativeness. It directly affects whether pooled estimates can be

interpreted as evidence of global applicability. I suggest that the authors explicitly discuss the

possibility that the pooled performance estimates may conceal clinically important failures in under-

represented regions. This is especially relevant for Africa and Latin America, where arboviral

burden is substantial but the available AI diagnostic evidence remains limited.

3. Calibration deserves stronger emphasis

Calibration is reported in only 5 of 47 included studies. This is one of the most important findings

of the review and should receive greater emphasis. Calibration is not a secondary technical detail.

For diagnostic and triage models, it is central to whether predicted probabilities correspond to actual

event rates.

A model with excellent AUC but poor calibration may rank patients correctly on average while still

producing misleading absolute risk estimates. This is particularly problematic if the model is

deployed in populations with different disease prevalence from the training or validation datasets. In

triage contexts, miscalibrated probabilities may lead to inappropriate reassurance, unnecessary

testing, missed cases, or inefficient use of scarce resources.

The manuscript currently notes the scarcity of calibration reporting, but I recommend making this a

more prominent interpretive point in the discussion and conclusion. The authors could also

recommend that future studies report calibration plots, calibration intercept and slope, Brier score,

and decision-curve analysis where appropriate.

4. The scope of the title and abstract should be aligned with the actual evidence base

The eligibility criteria include dengue, Zika, chikungunya, West Nile virus, yellow fever, and Rift

Valley fever. However, the included evidence is overwhelmingly about dengue, with limited

evidence for Zika and chikungunya and no included studies addressing West Nile virus, yellow

fever, or Rift Valley fever.

This absence is important and should be discussed more explicitly. As currently framed, the title

and abstract may suggest broader evidence across mosquito-borne viral infections than the included

studies actually support. The authors should either narrow the language in the title and abstract or

clearly state that the quantitative findings are driven predominantly by dengue studies and cannot be

generalized to West Nile virus, yellow fever, or Rift Valley fever.

A sentence in the abstract noting that no eligible studies addressed West Nile virus, yellow fever, or

Rift Valley fever would improve transparency and help readers interpret the scope of the

conclusions appropriately.

5. The interpretation of pooled estimates should be more cautious given extreme

heterogeneity

The manuscript reports very high heterogeneity across pooled analyses, often with I² values of

100%. This substantially limits the interpretability of the pooled estimates. Although the authors

acknowledge heterogeneity, the results and discussion sometimes still present the pooled estimates

as if they provide a stable summary of diagnostic performance.

I recommend adopting more cautious language throughout. The pooled estimates are useful as

broad descriptive benchmarks, but they should not be interpreted as precise or generalizable

estimates of expected model performance. The authors could make clearer that the meta-analysis

synthesizes a highly heterogeneous set of model types, study designs, populations, reference

standards, predictors, validation strategies, and target conditions.

6. Risk of bias should be more clearly linked to interpretation of performance

The PROBAST assessment is a strength of the manuscript. However, the implications of the risk-

of-bias findings could be integrated more strongly into the interpretation of diagnostic performance.

Since 32 of 47 studies were judged at high overall risk of bias, high reported performance should be

interpreted with caution.

The discussion could explicitly connect high AUCs and near-perfect accuracies to common

methodological weaknesses such as small sample sizes, retrospective designs, internal-only

validation, possible overfitting, incomplete reporting of missing data handling, and lack of

prospective evaluation.

**Reviewer #4:**  Overall assessment

I would like to begin by congratulating the authors on a substantial and timely piece of work. Synthesising 47 studies on AI/ML models for the early diagnosis of mosquito-borne viral infections is no small undertaking, and the effort shows: the search strategy is comprehensive and fully reproducible, the screening and extraction process is sound, the narrative synthesis by model category is clear and well organised, and the use of PROBAST to appraise risk of bias is a genuine strength. The breadth of evidence assembled here has real descriptive value and addresses a question of clear public health relevance.

After a detailed reading of the manuscript, the supplementary material, and all of the figures (including the forest plots and funnel plots in the S1 Appendix), my main reservations concentrate on the meta-analytic component, which is also the manuscript’s principal claim to novelty. I believe the pooled estimates, as currently presented, are affected by several issues of scale, variance estimation, unit of analysis, and publication-bias assessment that need to be addressed before the quantitative synthesis can be considered reliable. I want to stress that these are addressable, and that the underlying dataset and the rest of the manuscript provide a solid foundation to build on. For these reasons I recommend a major revision, centered on reworking the meta-analysis. Below I set out the main points first, with concrete examples drawn from the authors’ own figures, followed by minor points. I hope these comments are read in the constructive spirit in which they are intended.

Major points

1. Mismatch between the statistical method described and the one applied. Section 2.5 states that the effect size was computed as a standardised mean difference and reported as Cohen’s d. However, the forest plots in the S1 Appendix make clear that what is being pooled are the diagnostic metrics expressed as percentages (the “ES” column shows, for example, AUC values of 99.00, 87.00, 95.00…), and the axes of Figures 6–8 are on a 0–100 scale rather than in units of Cohen’s d. There is therefore an inconsistency between the Methods and what was actually analysed. Pooling proportions bounded in [0,1] requires an appropriate transformation (logit or Freeman-Tukey double-arcsine) before random-effects pooling. In addition, for diagnostic tests reporting paired sensitivity and specificity, the current methodological standard is the bivariate or HSROC model (Reitsma; Rutter-Gatsonis), which preserves the correlation between the two metrics and the threshold dependency; pooling them separately, as done here, is not ideal. I would ask the authors to clarify precisely what was computed and, where needed, to rerun the meta-analysis using methods appropriate for proportions and diagnostic accuracy.

2. A demonstrable unit error contaminating the AUC pool. In the random-effects AUC forest plot (S1 Appendix, Fig 1b), the study Tanner L DT 2008 is entered with an effect size of 0.88 (CI 0.81–0.95), whereas every other study is on the 0–100 scale (e.g., 99.00, 95.00, 81.00). In other words, an AUC of 0.88 was not rescaled to 88.00. This single value is the extreme point that appears next to 0 in Figure 6 (AUC panel) and in the funnel plots (Figures 7–8), and it distorts the pooled estimate, the funnel shape, and the Egger test. Notably, in the accuracy forest plot (Fig 2b) the same Tanner study appears correctly as 84.00, which confirms this is a scale error specific to the AUC metric. I would encourage a cell-by-cell audit of the extraction sheet to identify and correct any remaining unit inconsistencies before re-pooling.

3. Implausible per-study confidence intervals, pointing to an error in variance estimation. The supplementary forest plots show degenerate or near-zero intervals: for example, Das S NN 2025 with ES 99.90 (CI 99.90–99.90) and N=90, or Abdualgalil B 2022 with ES 99.00 (CI 98.99–99.01) and N=6694. An AUC or accuracy around 0.99, with sample sizes of tens or a few thousand, cannot have a confidence interval of essentially zero width. This explains the zero-width intervals seen in the fixed-effect models in the main text (e.g., AUC 98.46, CI 98.46–98.46; accuracy 99.59, CI 99.59–99.59) and indicates that the issue is not limited to model choice: the within-study precision (standard error) appears to have been computed incorrectly. I would ask that the variance computation be reviewed.

4. Inapplicable fixed-effect models and a non-interpretable publication-bias assessment. With I²=100% across all outcomes, the fixed-effect model should not be presented as a valid estimate, and the zero-width intervals it produces confirm this. The funnel plots (Figures 7–8), for their part, have the “effect size” axis on a percentage scale (0–120) and include points at zero and negative values, which are impossible for AUC, sensitivity, or PPV; this explains the implausibly large Egger intercepts reported in the text (e.g., −276.89 for AUC, −189.57 for accuracy). As it stands, the publication-bias analysis cannot be interpreted. I would recommend removing the fixed-effect estimates, recomputing on the correct transformed scale, and reconsidering the suitability of the Egger test (which, in any case, has limited power with fewer than ten studies per outcome).

5. Calibrating the conclusions to the strength of the evidence. The analysis itself documents that 68% of studies are at high overall risk of bias (PROBAST), that heterogeneity is extreme (I²=100%) across all outcomes, that external validation and calibration are infrequent, and that there is possible publication bias for accuracy and sensitivity. Given this, the tone of the abstract and discussion (“high diagnostic performance”, “promising”, and the presentation of the pooled value as a “model-agnostic quantitative benchmark”) reads as somewhat more optimistic than the evidence supports. Once the issues above are corrected, I would suggest presenting the pooled estimates explicitly as exploratory descriptive indicators of a highly heterogeneous and potentially biased literature, rather than as a reliable measure of real-world model performance. A measured softening of the claims throughout would, in my view, strengthen the paper.

6. Scope of the review question. The title and objective focus on early diagnosis, but several included studies have prognosis, severity, or serotyping as their outcome: severity prediction (Potts 2010), hospitalisation prediction via the SISA index (Sippy 2020), and infecting-serotype prediction (Daniels 2024). In addition, at least one study is based on a fully synthetic dataset (Mahalakshmi 2019), whose weight in a synthesis of real diagnostic performance is debatable. Mixing early diagnosis with prognosis, severity, and serotyping blurs the PICO question and contributes to the observed heterogeneity. I would ask the authors to justify or reconsider the inclusion of these studies and to define the review question more precisely.

Minor points

8. PRISMA flow inconsistencies between text and figure. Section 3.1 states “115 articles for full-text examination”, but the arithmetic (1,969 − 1,861 = 108) and Figure 1 itself (“Reports sought for retrieval, n=108”) confirm that the correct figure is 108. The sentence “61 articles were excluded, including 47 records” is also inconsistent: per Figure 1, the eligibility-stage exclusions sum to 54 (32+14+6+2), leaving 47 included. The figure is correct; the text figures should be corrected (“115”→108, and the exclusions sentence).

9. Possible errors in study metadata. (a) Husin, N.A. (2020) is assigned to Yemen in Table 1, but reference [50] (J Theor Appl Inf Technol) and the authors’ affiliations suggest Malaysia; the country should be verified, as it affects the country count, the phrasing “Yemen [49,50]”, and the map in Figure 2 (where Yemen is shown with 2 studies). (b) The year for Goh, B. differs between Table 1 (2025) and Table 3 of the appendix (2023); please reconcile. (c) The two “Mayrose, H. (2023)” entries correspond to two distinct articles ([29] and [30]); distinguishing them with suffixes (2023a/2023b) would avoid the appearance of duplication. (d) Please verify the spelling of the surname “Sippyid”, likely “Sippy”.

10. Contradiction regarding the use of imaging. The text (lines 335-336) and Figure 3 state that no study incorporated imaging data, yet there are studies based on blood-smear images (Mayrose), and Figure 4 itself highlights “Blood smear images” and “Image- and Signal-based Diagnostics” as a category. The coding of input variables should be reconciled so that the text, Figure 3, and Figure 4 are consistent.

11. Reporting of heterogeneity and presentation of forest plots. (a) I²=100% is reported identically across all outcomes; it would help to provide the exact I² and τ² (or τ) values for each synthesis, along with prediction intervals, which are more informative than the CI of the pooled estimate when heterogeneity is high. (b) The forest plots include a per-study “Sig. 0.000” column whose meaning (the null hypothesis being tested) is unclear; I would suggest removing or explaining it.

12. Reporting and appraisal frameworks. As this is a review of the diagnostic accuracy of prediction models, it would be more appropriate to align the reporting with PRISMA-DTA (in addition to PRISMA 2020) and to consider TRIPOD+AI as a reference framework for appraising the primary studies. The use of PROBAST is appropriate, but I would recommend: (a) correcting the Figure 5 legend, which mentions “five domains” whereas PROBAST has four domains (participants, predictors, outcome, analysis) plus the overall judgement; (b) also including the PROBAST applicability component; and (c) providing the per-study, per-domain judgements in supplementary material, not only the aggregate plot.

13. Data availability. The current statement (“the extracted dataset and generated code are available from the corresponding author upon reasonable request”) does not align with the PLOS data policy, which requires unrestricted availability except where there is a justified legal or ethical restriction. As this is a systematic review without individual patient data, there appears to be no obstacle to depositing the extraction sheet, the full dataset, and the analysis code in a public repository (e.g., OSF, Zenodo, or figshare) with a corresponding DOI. I would warmly encourage public deposition before acceptance; it would also allow the corrections in points 1–5 to be verified.

14. Minor stylistic points. Some p-values are written without an equals sign (e.g., “p 0.015”, “p 0.038”); please standardise to “p = ”. The appendix figures are cited as “Figure 1 in S1 Appendix”, which can be confused with Figure 1 of the manuscript; the PLOS supplementary-figure numbering (S1 Fig, S2 Fig, etc.) would be clearer. Please also check the consistent spelling of “Sa-Ngamuang/Sa-ngamuang”.

Recommendation

This manuscript addresses a relevant question and contributes a valuable body of evidence, and the authors are to be commended for the scope and care of the work to date. My principal concern is that the meta-analysis, in its current form, is affected by demonstrable errors of scale, variance estimation, and unit of analysis, together with a statistical model that is not well suited to the nature of the metrics and a publication-bias assessment that cannot currently be interpreted.

Addressing these will require reworking the quantitative synthesis with appropriate methods, auditing the extraction sheet, tempering the conclusions, and resolving the numerical and reporting inconsistencies noted above. I am confident these improvements are achievable, and that once implemented the work will make a worthwhile contribution to the literature. I therefore recommend a major revision, and I would be glad to review a revised version.

**Reviewer #5:**  Many thanks for your invitation. This study conducted to Diagnostic performance of Artificial Intelligence in early diagnosis of mosquito borne viral infections: systematic review and meta-analysis, this is a good manuscript. Minor revision to improve the quality of the article before publication, is helpful.

- All sections must be prepared according to the journal format.

- The statistical approach used for pooling diagnostic accuracy outcomes appears inappropriate. Sensitivity, specificity, PPV, NPV, and AUC should not be synthesized using standardized mean differences (Cohen's d). Diagnostic accuracy meta-analyses generally require hierarchical models such as the bivariate random-effects model or HSROC model to account for the correlation between sensitivity and specificity. The authors should justify their analytical approach or reanalyse the data using accepted DTA meta-analysis methodology.

- It would be better if they explained exactly why they chose PROBAST and whether another tool would not be more appropriate for purely diagnostic studies.

- It is expected that analyses such as: Type of disease (Dengue vs Zika vs Chikungunya), Type of AI algorithm, Type of data (clinical/laboratory), Type of validation, Country, Study design, will be performed as meta-regression or subgroup analysis.

- For meta-analysis of diagnostic accuracy, the Egger test is not usually recommended.

The standard method is: Deeks' funnel plot asymmetry test.

**********

6. PLOS authors have the option to publish the peer review history of their article (what does this mean?). If published, this will include your full peer review and any attached files.

**Do you want your identity to be public for this peer review?**  If you choose “no”, your identity will remain anonymous but your review may still be made public.

For information about this choice, including consent withdrawal, please see our Privacy Policy.

Reviewer #1: No

Reviewer #2: **Yes:**  Seng Hansun

Reviewer #3: **Yes:**  Omar Enzo Santangelo

Reviewer #4: No

Reviewer #5: No

**********

[NOTE: If reviewer comments were submitted as an attachment file, they will be attached to this email and accessible via the submission site. Please log into your account, locate the manuscript record, and check for the action link "View Attachments". If this link does not appear, there are no attachment files.] **Figure resubmission:**  While revising your submission, we strongly recommend that you use PLOS’s NAAS tool (https://ngplosjournals.pagemajik.ai/artanalysis) to test your figure files. NAAS can convert your figure files to the TIFF file type and meet basic requirements (such as print size, resolution), or provide you with a report on issues that do not meet our requirements and that NAAS cannot fix.

After uploading your figures to PLOS’s NAAS tool - https://ngplosjournals.pagemajik.ai/artanalysis, NAAS will process the files provided and display the results in the "Uploaded Files" section of the page as the processing is complete. If the uploaded figures meet our requirements (or NAAS is able to fix the files to meet our requirements), the figure will be marked as "fixed" above. If NAAS is unable to fix the files, a red "failed" label will appear above. When NAAS has confirmed that the figure files meet our requirements, please download the file via the download option, and include these NAAS processed figure files when submitting your revised manuscript. **Reproducibility:**  To enhance the reproducibility of your results, we recommend that authors of applicable studies deposit laboratory protocols in protocols.io, where a protocol can be assigned its own identifier (DOI) such that it can be cited independently in the future. Additionally, PLOS ONE offers an option to publish peer-reviewed clinical study protocols. Read more information on sharing protocols at https://plos.org/protocols?utm_medium=editorial-email&utm_source=authorletters&utm_campaign=protocols

Attachment Submitted filename: REVIEW COMMENTS TO THE AUTHOR.docx

# Author comment: Author response to Decision Letter 1

**DOI:** 10.1371/journal.pdig.0001796.r002

28 Aug 2026

Attachment Submitted filename: REVIEW COMMENTS TO THE AUTHOR 27.08.2026.docx

# Peer review: Decision Letter 1

**DOI:** 10.1371/journal.pdig.0001796.r003

**Authors:** Kaisaierjiang Kadier, Tim M.H. Li

6 Oct 2026

Diagnostic performance of Artificial Intelligence in early diagnosis of mosquito-borne viral infections: systematic review and meta-analysis

PDIG-D-26-01019R1

Dear Dr Cozzolino Cangiano,

We are pleased to inform you that your manuscript 'Diagnostic performance of Artificial Intelligence in early diagnosis of mosquito-borne viral infections: systematic review and meta-analysis' has been provisionally accepted for publication in PLOS Digital Health.

Before your manuscript can be formally accepted you will need to complete some formatting changes, which you will receive in a follow-up email from a member of our team.

Please note that your manuscript will not be scheduled for publication until you have made the required changes, so a swift response is appreciated.

IMPORTANT: The editorial review process is now complete. PLOS will only permit corrections to spelling, formatting or significant scientific errors from this point onwards. Requests for major changes, or any which affect the scientific understanding of your work, will cause delays to the publication date of your manuscript.

If your institution or institutions have a press office, please notify them about your upcoming paper to help maximize its impact. If they'll be preparing press materials, please inform our press team as soon as possible -- no later than 48 hours after receiving the formal acceptance. Your manuscript will remain under strict press embargo until 2 pm Eastern Time on the date of publication. For more information, please contact digitalhealth@plos.org.

Thank you again for supporting Open Access publishing; we are looking forward to publishing your work in PLOS Digital Health.

Best regards,

Kaisaierjiang Kadier

Guest Editor

PLOS Digital Health

***********************************************************

**Additional Editor Comments (if provided):**

**Reviewer Comments (if any, and for reference):**

Reviewer's Responses to Questions

**Comments to the Author**

1. If the authors have adequately addressed your comments raised in a previous round of review and you feel that this manuscript is now acceptable for publication, you may indicate that here to bypass the “Comments to the Author” section, enter your conflict of interest statement in the “Confidential to Editor” section, and submit your "Accept" recommendation.

Reviewer #2: All comments have been addressed

Reviewer #3: All comments have been addressed

**********

2. Does this manuscript meet PLOS Digital Health’s publication criteria? Is the manuscript technically sound, and do the data support the conclusions? The manuscript must describe methodologically and ethically rigorous research with conclusions that are appropriately drawn based on the data presented.

Reviewer #2: (No Response)

Reviewer #3: Yes

**********

3. Has the statistical analysis been performed appropriately and rigorously?

Reviewer #2: (No Response)

Reviewer #3: Yes

**********

4. Have the authors made all data underlying the findings in their manuscript fully available (please refer to the Data Availability Statement at the start of the manuscript PDF file)?

The PLOS Data policy requires authors to make all data underlying the findings described in their manuscript fully available without restriction, with rare exception. The data should be provided as part of the manuscript or its supporting information, or deposited to a public repository. For example, in addition to summary statistics, the data points behind means, medians and variance measures should be available. If there are restrictions on publicly sharing data—e.g. participant privacy or use of data from a third party—those must be specified.

Reviewer #2: (No Response)

Reviewer #3: Yes

**********

5. Is the manuscript presented in an intelligible fashion and written in standard English?

PLOS Digital Health does not copyedit accepted manuscripts, so the language in submitted articles must be clear, correct, and unambiguous. Any typographical or grammatical errors should be corrected at revision, so please note any specific errors here.

Reviewer #2: (No Response)

Reviewer #3: Yes

**********

6. Review Comments to the Author

Please use the space provided to explain your answers to the questions above. You may also include additional comments for the author, including concerns about dual publication, research ethics, or publication ethics. (Please upload your review as an attachment if it exceeds 20,000 characters)

Reviewer #2: (No Response)

Reviewer #3: All comments have been addressed

**********

7. PLOS authors have the option to publish the peer review history of their article (what does this mean?). If published, this will include your full peer review and any attached files.

**Do you want your identity to be public for this peer review?**  If you choose “no”, your identity will remain anonymous but your review may still be made public.

For information about this choice, including consent withdrawal, please see our Privacy Policy.

Reviewer #2: None

Reviewer #3: **Yes:**  Omar Enzo Santangelo

**********
